# Early2 factor (E2F) deregulation is a prognostic and predictive biomarker in lung adenocarcinoma

**DOI:** 10.18632/oncotarget.12672

**Published:** 2016-10-14

**Authors:** Lu Chen, Courtney A Kurtyka, Eric A Welsh, Jason I Rivera, Brienne E Engel, Teresita Muñoz-Antonia, Sean J Yoder, Steven A Eschrich, Ben C Creelan, Alberto A Chiappori, Jhanelle E Gray, Jose Luis Ramirez, Rafael Rosell, Matthew B Schabath, Eric B Haura, Dung-Tsa Chen, Douglas W Cress

**Affiliations:** ^1^ Biostatistics and Bioinformatics, H. Lee Moffitt Cancer Center & Research Institute, Tampa, Florida, USA; ^2^ Cancer Biology and Evolution, H. Lee Moffitt Cancer Center & Research Institute, Tampa, Florida, USA; ^3^ Tumor Biology, H. Lee Moffitt Cancer Center & Research Institute, Tampa, Florida, USA; ^4^ Molecular Genomics Core Facility, H. Lee Moffitt Cancer Center & Research Institute, Tampa, Florida, USA; ^5^ Thoracic Oncology, H. Lee Moffitt Cancer Center & Research Institute, Tampa, Florida, USA; ^6^ Cancer Biology & Precision Medicine Program, Catalan Institute of Oncology, Badalona, Barcelona, Spain; ^7^ Cancer Epidemiology, H. Lee Moffitt Cancer Center & Research Institute, Tampa, Florida, USA

**Keywords:** lung adenocarcinoma, adjuvant chemotherapy, E2F, predictive biomarker, prognostic biomarker

## Abstract

Clinicians routinely prescribe adjuvant chemotherapy (ACT) for resected non-small cell lung cancer patients. However, ACT only improves five-year disease-free survival in stage I-III non-small cell lung cancer by 5-15%, with most patients deriving no benefit. Herein, deregulation of the E2F pathway was explored as a biomarker in lung adenocarcinoma patients. An E2F pathway scoring system, based on 74 E2F-regulated genes, was trained for RNA from two platforms: fresh-frozen (FF) or formalin-fixed paraffin-embedded (FFPE) tissues. The E2F score was tested as a prognostic biomarker in five FF-based cohorts and two FFPE-based cohorts. The E2F score was tested as a predictive biomarker in two randomized clinical trials; JBR10 and the NATCH (Neo-Adjuvant Taxol-Carboplatin Hope) trial. The E2F score was prognostic in untreated patients in all seven datasets examined (*p* < 0.05). Stage-specific analysis of combined cohorts demonstrated that the E2F score was prognostic in stage I patients (*p* = 0.0495 to <0.001; hazard ratio, HR, =2.04- 2.22) with a similar trend in other stages. The E2F score was strongly predictive in stage II patients from the two combined randomized clinical trials with a significant differential treatment effect (*p* = 0.015). Specifically, ACT improved survival in stage II patients with high E2F (*p* = 0.01; HR= 0.21). The 5-year survival increased from 18% to 81%. In contrast, in patients with low E2F, 5-year survival was 57% in untreated patients and 41% in ACT-treated patients with a HR of 1.55 (*p* = 0.47). In summary, the E2F score provides valuable prognostic information for Stage I and predictive information for Stage II lung adenocarcinoma patients and should be further explored as a decision support tool for their treatment.

## INTRODUCTION

Breast cancer patients have long received the benefit of prognostic gene expression-based classifiers such as Oncotype DX [1] (16 genes), Prosigna [2] (50 genes) and MammaPrint [3] (70 genes). The MammaPrint test, for example, divides early-stage breast cancer patients into two approximately equal groups; those patients that can forego ACT, without significant risk of decreased survival, and those that are likely to benefit from ACT. These tests save healthcare dollars, reduce the morbidity of ACT in low-risk patients, and assure high-risk patients that ACT is the right choice for them. In spite of the fact that lung cancer accounts for more deaths per year in the US, than breast, colon, prostate, and pancreatic cancer combined [4], lung cancer patients do not generally benefit from similar biomarkers [5]. Clinicians routinely prescribe adjuvant chemotherapy (ACT) for resected NSCLC patients; however, ACT only improves the proportion of five-year disease-free survivors in stage I-III non-small cell lung cancer by 5-15% when no markers are used to select patients for chemotherapy [6–12]. The remaining 85-95% of patients derive no measurable benefit and suffer the adverse effects and risks of treatment. While tumor stage [6] and histological subtype within adenocarcinomas [13] can guide the decision to treat with ACT, a robust, well-validated marker that could clearly identify which patients should receive adjuvant chemotherapy and which should be targeted for other strategies (observation, clinical trials, novel agents) would be of significant clinical value.

The majority of lung cancers (~85%) are classified as non-small cell lung cancer (NSCLC). The most common histological subtype of NSCLC is lung adenocarcinoma. We and others have demonstrated that proliferative capacity drives patient outcome and aggressiveness in lung adenocarcinomas, and gene expression signatures reflecting this capacity have been shown to be highly prognostic [14–17]. With proliferation tightly regulated by the E2F pathway [18], direct assessment of the E2F pathway deregulation in clinical samples should provide prognostic information. Furthermore, the E2F pathway is central to the cellular response to DNA damaging agents [19, 20] and other compounds [21] used in the treatment of lung adenocarcinoma. Based on these observations, we hypothesized that an accurate measurement of E2F pathway activation in lung adenocarcinoma could potentially serve as a prognostic biomarker as well as a predictive biomarker for the benefit of ACT.

To explore this hypothesis, a 74-gene E2F signature was developed and trained for application with RNA from both fresh-frozen (FF) and formalin-fixed paraffin-embedded (FFPE) tissues. This 74-gene E2F signature was cross-validated as prognostic in seven different survival datasets. Most importantly, data from two independent phase III, randomized clinical trials were used to validate the signature as predictive of benefit of ACT: the JBR10 trial [22] and the (Neo)Adjuvant Taxol/Carboplatin Hope (NATCH) trial [7].

## RESULTS

### E2F Signature development and optimization

**Figure 1:**
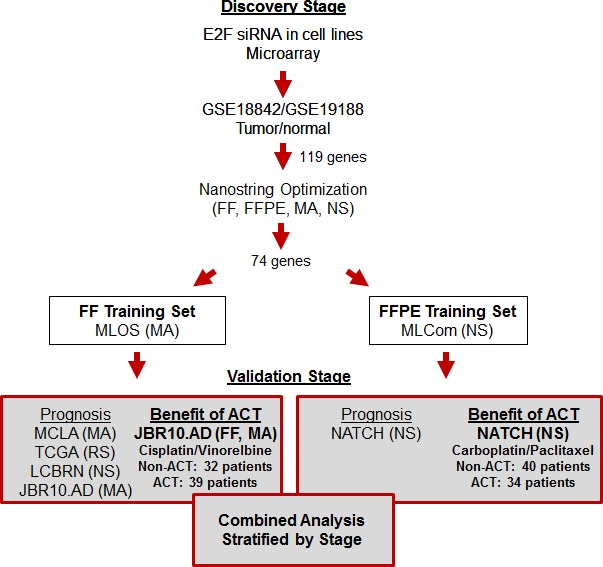
Study Overview This figure highlights the discovery steps used to define the 74 genes in the E2F scoring system and the subsequent steps and datasets used to validate the prognostic and predictive effects of the E2F score. Experiments that highlight the ability of the E2F assay to predict the benefit of ACT in lung adenocarcinoma surgical patients are in bold. Abbreviations: FF: Fresh Frozen; FFPE: Formalin-Fixed Paraffin-Embedded; MA: Microarray; RS: RNA sequencing; NS: NanoString^TM^; ACT: Adjuvant chemotherapy; MCLA: Molecular Classification of Lung Adenocarcinoma; TCGA: The Cancer Genome Atlas; JBR10: National Cancer Institute of Canada, Cancer Center Therapeutics Group; JBR10.AD: the adenocarcinoma subset of JBR10; LCBRN: Lung Cancer Bio-specimen Resource Network; NATCH: (Neo)Adjuvant Taxol/Carboplatin Hope.

The overall schema used to develop an E2F pathway scoring system is highlighted in Figure 1. Multiple datasets (Tables S1-S3) were used to develop and validate the final 74 gene E2F signature (Tables S4-5) as described in the Materials and Methods and in greater detail in the Supplementary Materials. Specifically, siRNAs were used to knock down E2F pathway components (E2F1, E2F3A, E2F3B, both 3A and 3B, E2F4, and Rb) in model cell lines. Gene-specific knock down was confirmed by Western blotting (see Figure S1). Microarray analysis compared siRNA-treated knockdown versus control A549 and H1299 cell lines and applied a number of filters to identify 119 coding genes (145 probesets). Biologically these were highly correlated with cell cycle and DNA damage response by GeneGo analysis (see Table S6). Among the 119 genes, 106 genes were found well-annotated and thus incorporated into a NanoString assay. Comparison of individual gene expression between FF and FFPE in the “MLTO” cohort revealed 32 genes with poor individual correlation (*r* < 0.5; Table S7 and Figure S2), and led to a 74-gene signature. The PC1 scores derived from the 74 gene signature and the original 106 genes had strong correlations (FF: *r* = 0·99, *p* < 0·001; FFPE: *r* = 0·98-0.99, *p* < 0·001; Figure S3; with a similar percentage of total variation; 29-30%; Figure S4), suggesting that the remaining 74 genes reflected the original biology of the larger list. Further correlation analysis (Figure S5) among FF in microarray and FF and FFPE in NanoString showed a weak to moderate reproducibility in PC1 score of the 74-gene signature (*r* = 0.3-0.78), indicating non-negligible variation by tissue type.

To adjust for variation due to tissue types, and therefore allow comparison of data from diverse cohorts, the E2F scoring system was developed in two platforms based on either FF or FFPE tissue. Both platforms used the PC1 loading coefficients (gene weights) to calculate the E2F score. The gene weights were derived using the MLOS cohort for the FF platform while the MLCom cohort was used to obtain the gene weights for the FFPE platform. The percentage of total variation for PC1 between the two platforms was comparable (24-26%; Figure S6). While the correlation of the two platforms was weak (*r* = 0.25-0.28; Figure S7 and Table S8), both platforms gave a similar range of gene weights (-0.165 to 0.223 in FF and -0.165 to 0.210 in FFPE).

Evaluation of the median threshold was performed in the two training cohorts: the MLOS cohort for the FF platform and the MLCom cohort for the FFPE platform. In the FF platform, the classification by the median E2F score was significantly associated with OS in non-ACT patients of the MLOS cohort with poor OS in high E2F group (*p* < 0.001). Interestingly, other cutoffs (25^th^-75^th^ percentiles) also had a significant association, indicating that the E2F score is generally robust in prognosis (Figure S8). Similarly, in the FFPE platform, the median-cutoff classification was able to significantly separate the low and high E2F groups in non-ACT patients of the MLCom cohort in terms of OS and PFS (*p* = 0.041 for OS and *p* = 0.044 for PFS). In comparison, other cutoffs were significant only in the range of 40^th^-60^th^ percentiles for OS and in the range of 25^th^-75^th^ percentiles for PFS (Figure S9). While the median-cutoff did not give the smallest p value, the median-cutoff E2F classification was associated with OS or PFS in both platforms, justifying the median threshold for risk classification. The post-hoc evaluation of training and validation cohorts also support the validity of a median-cutoff classification for the E2F score (Figure S8-9 and Table S9-12).

### The E2F Score is a prognostic marker

**Figure 2:**
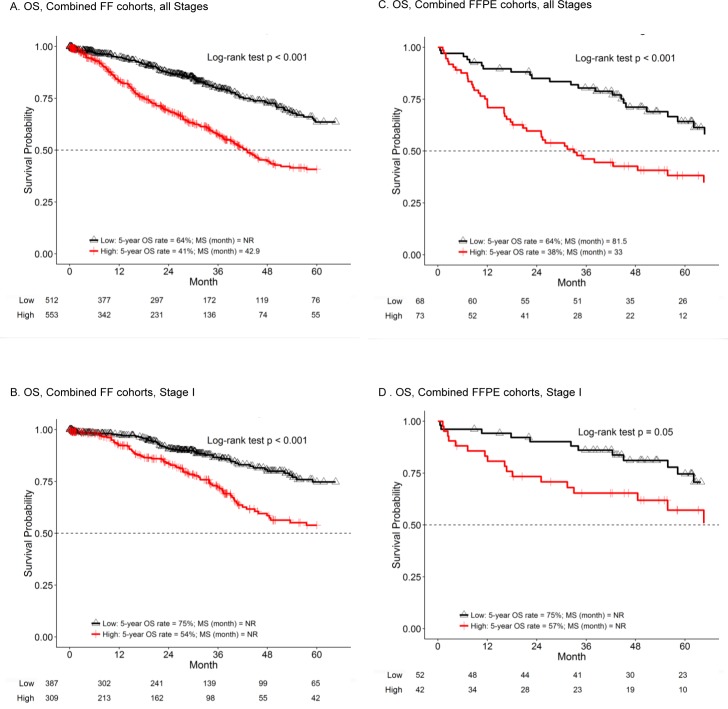
The E2F score is prognostic in multiple lung adenocarcinoma datasets K-M analysis of OS in the indicated combined cohorts was performed comparing patients with high (red line) or low E2F score (black line). Results for 5-year survival and the log-rank test p value are included in each panel. Numbers at the bottom of the graph indicate the number of patients in each group at risk at 12-month intervals. MS represents median survival time and NR means the MS was never reached. Graphs are truncated at 60 months. **A.** and **B.** represent combined FF cohorts. **C.** and **D.** represent FFPE cohorts. A and C include patients of all stages. B and D include only stage I patients.

The derived E2F score was first tested as a prognostic marker in seven cohorts of resected lung adenocarcinoma patients who did not receive adjuvant chemotherapy. These data, summarized in Table 1, reveal the prognostic value of the E2F score by the log-rank test (Table S13 presents detailed analysis within each cohort, including PFS). Specifically, in the FF platform, each of the five cohorts showed a significant prognostic effect with poor OS in all non-ACT patients with a high E2F score. For stage-specific analysis, the combined FF cohorts (MLOS, MCLA, TCGA, JBR10.AD and LCBRN) demonstrated a strong association between OS and E2F scores in all non-ACT patients and in Stage I non-ACT patients (*p* < 0.001; HR = 2.38 for all stages and HR = 2.22 for stage I; Table 1) and this significance continued even with covariate adjustment (*p* < 0.001; HR = 1.88 and 2.16 for all stages and stage I, respectively; Table S14). The median survival time (MST) was 42.9 months in high E2F group and never reached within the study in low E2F group for all stage. MST was never reached for stage I due to longer survival in this subpopulation. For this reason, we used the 5-year survival rate for comparison. The 5-year survival rate increased at least 20% in low-E2F patients (all stages: low E2F: 64% (95% CI: 57% - 71%) versus high E2F: 41% (95% CI: 35% - 48%); stage I: low E2F: 75% (95% CI: 68% - 82%) versus high E2F: 54% (95% CI: 46% - 63%); Table I, Figure 2A and 2B). A similar prognostic trend was observed in stage II and combined stage III/IV patients, but the results were not statistically significant.

**Table 1:** Prognostic effects of the E2F score in resected lung adenocarcinoma patients who did not receive adjuvant chemotherapy

	Low E2F				High E2F											
Overall survival	N	Median survival	5-year Survival rate (%)		N	Median survival	5-year Survival rate (%)	Log-rank test p-value				HR** (95% CI) High vs. Low		
Fresh Frozen: Individual cohorts															
MLOS[33] All stages (N=300)	156	NR*	73		144	40.4	33		**<0.001**		2.58 (1.59, 4.17)		
MCLA[34] All stages (N=233)	119	NR	72		114	NR	50		**<0.001**		2.32 (1.48, 3.64)		
TCGA[36] All stages (N=436)	185	53.3	35		251	35.8	29		**0.008**		1.74 (1.15, 2.64)		
JBR10.AD[22] All stages (N=32)	21	NR	71		11	36.4	22		**0.007**		3.9 (1.35, 11.32)		
LCBRN All stages (N=64)	31	NR	89***		33	40.8	69***		**0.028**		4.74 (1.02, 21.97)		
Combined FF cohorts (MLOS, MCLA, TCGA, LCBRN, JBR10.AD)															
All Stages (N=1065)	512	NR	64		553	42.9	41		**<0.001**		2.38 (1.86, 3.05)		
Stage I (N=696)	387	NR	75		309	NR	54		**<0.001**		2.22 (1.55, 3.17)		
Stage II (N=202)	70	50	31		132	28.8	29		0.061		1.58 (0.97, 2.55)		
Stage III/IV (N=159)	50	35.4	24		109	20.8	18		0.053		1.65 (0.99, 2.75)		
**FFPE:** Individual cohorts															
MLCom All stages (N=101)	55	75.5	62		46	42.6	50		**0.041**		1.87 (1.02, 3.44)		
NATCH All stages (N=40)	13	81.5	69		27	25.1	20		**0.014**		3.26 (1.21, 8.78)		
Combined FFPE cohorts (MLCom and NATCH)															
All Stages (N=141)	68	81.5	64		73	33	38		**<0.001**		2.29 (1.39, 3.76)		
Stage I (N=94)	52	NR	75		42	NR	57		**0.0495**		2.04 (0.99, 4.21)		
Stage II (N=19)	6	64.7	62		13	16.1	23		0.0501		3.54 (0.93, 13.4)		
Stage III/IV (N=28)	10	25	13		18	19.6	7		0.357		1.51 (0.63, 3.6)		

NR*: median survival time not reached within the study

HR**: hazard ratio

*** indicates 3-year survival cutoff since 5-year survival rate was not estimated due to short follow-up

Significant P values are in bold text

Both FFPE-based cohorts (MLCom and NATCH) had a significant association of the E2F score with OS (p = 0.01-0.04; HR = 1.87-3.26; Table 1) and PFS (*p* = 0.03-0.04; HR = 1.75-2.57; Table S13) in all non-ACT patients (stages I-IV). The combined cohort (MLCom + NATCH) also exhibited statistically significant association with OS (*p* < 0.001; HR = 2.29; Table 1) and PFS (*p* < 0.001; HR = 2.14; Table S13) in all patients. Patients with low E2F had a longer survival than the high E2F patients (MST = 81.5 months versus 33 months). For stage I patients, the significance level was borderline for OS (*p* = 0.0495; HR = 2.04; Table 1) and PFS (*p* = 0.046; HR = 1.84; Table S13). Covariate-adjusted association was significant (OS or PFS) in the NATCH cohort (*p* = 0.03-0.04; HR = 2.59-2.94; Table S14) and the combined cohort (*p* = 0.01-0.03; HR = 1.80-1.81; Table S14) in all stage patients. In terms of 5-year survival rate, it increased at least 15% in low-E2F patients (all stages: low E2F: 64% (95% CI: 53% - 78%) versus high E2F: 38% (95% CI: 28% - 52%); stage I: low E2F: 75% (95% CI: 62% - 89%) versus high E2F: 57% (95% CI: 43% - 77%); Table 1, Figure 2C and 2D). Additional analysis in the combined FF and FFPE cohorts (JBR10.AD + NATCH) from the two randomized clinical trials again exhibited statistically significant association with OS (with or without covariate adjustment) in all stages (*p* = 0.004 to < 0.001; HR = 2.87-3.75) and stage I (*p* = 0.01-0.008; HR = 4.29-4.41) for non-ACT patients (Table S15-16).

**Figure 3:**
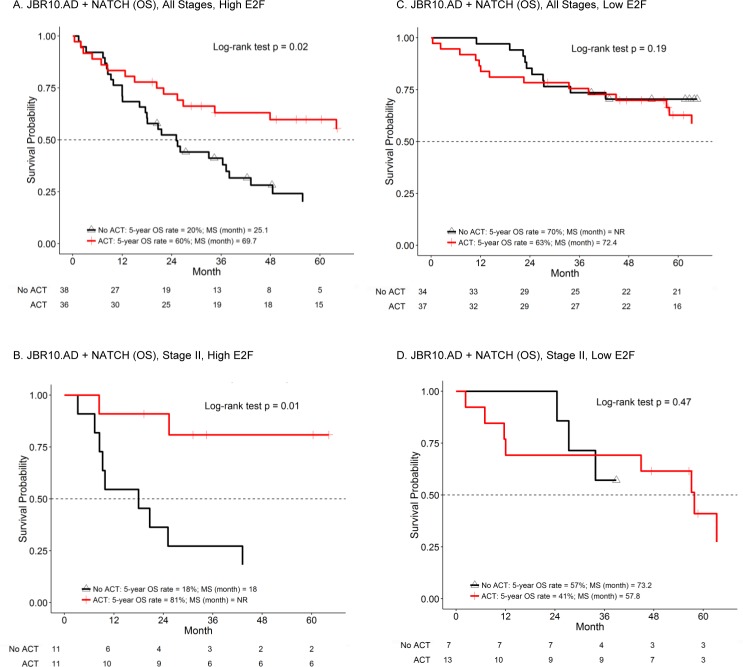
The E2F score predicts benefit of ACT in two randomized clinical trials K-M analysis of OS in the indicated combined cohorts was performed comparing patients with high E2F **A.** and **B.** with ACT (red line) or without ACT (black line) or low E2F **C.** and **D.** with ACT (red line) or without ACT (black line). Results for 5-year survival and the log-rank test p value are included in each panel. Numbers at the bottom of the graph indicate the number of patients in each group at risk at 12-month intervals. MS represents median survival time and NR means the MS was never reached. Graphs are truncated at 60 months. A and C represent patients of all stages. B and D represent stage II patients only.

### The E2F score predicts benefit of ACT

The E2F score was next tested as a predictive marker using two cohorts of lung adenocarcinoma patients, the JBR10 and NATCH trials, that were randomized to either surgery only or surgery followed by ACT. The predictive findings, summarized in Table 2, reveal the predictive value of the E2F score (see Tables S17-18 for additional details for each individual cohort and for PFS outcomes, as well as after adjustment for the effect of tissue type). Specifically, the combined cohort (JBR10.AD and NATCH) exhibited a significant differential ACT treatment effect in all stage patients (with or without covariate adjustment: *p* = 0.016-0.02 for the interaction effect; Table 2, Table S19, and Figure 3A). Subgroup analysis in the high E2F score group also showed a longer survival in patients with ACT (HR = 0.51 with *p* = 0.023-0.028; Table 2, Table S19, and Figure 3A). Moreover, the predictive effect was even stronger when considering only stage II patients (with or without covariate adjustment: *p* = 0.015-0.02 for the interaction effect and HR = 0.21-0.22 with *p* = 0.012-0.028 in the high E2F score group; Table 2, Table S19, and Figure 3B). Specifically, high-E2F patients demonstrated an increase in MST and 5-year survival (non-ACT: MST = 18 months and a 5-year survival rate of 18% (95% CI: 5% - 64%); ACT: MST = never reached and 5-year survival rate of 81% (95% CI: 60% - 100%); Table 2 and Figure 3B). In contrast, low-E2F patients demonstrated an opposite pattern: patients without ACT had a MST of 73.2 months and a 5-year survival rate of 57% (95% CI of 30% - 100%) while patients with ACT had a MST of 57.8 months and a 5-year survival rate of 41% (95% CI: 20% - 83%) (Table 2 and Figure 3D). Although the ACT treatment effect in stage I patients did not reach statistical significance, stage I patients with high E2F, 5-year survival increased from 31% without ACT to 61% with ACT. In contrast, in stage I patients with low E2F, 5-year survival was 83% in untreated patients and 77% in ACT-treated patients.

**Table 2:** Predictive effects of the E2F score in resected lung adenocarcinoma patients

Overall survival	No ACT				With ACT						
	N	Median survival	5-year Survival rate (%)		N	Median survival	5-year Survival rate (%)	Log-rank test p-value	HR** (95% CI) ACT vs No ACT	Int-P***	
Individual cohorts: JBR10.AD[22]: Fresh Frozen										
Low E2F group (N=48)	21	NR*	71		27	72.4	66	0.154	1.92 (0.77, 4.77)	**0.016**	
High E2F group (N=23)	11	36.4	22		12	73.1	75	**0.043**	0.31 (0.1, 1.01)		
**NATCH[7]: FFPE**										
Low E2F group (N=23)	13	81.5	69		10	NR	51	0.631	1.4 (0.35, 5.64)	0.407	
High E2F group (N=51)	27	25.1	20		24	65.6	52	0.162	0.61 (0.3, 1.23)		
**Combined cohorts (JBR10.AD and NATCH)**: **All stages**										
Low E2F group (N=71)	34	NR	70		37	72.4	63	0.195	1.62 (0.78, 3.36)	**0.016**	
High E2F group (N=74)	38	25.1	20		36	69.7	60	0.023	0.51 (0.28, 0.92)		
**Stage I**										
Low E2F group (N=46)	24	NR	83		22	NR	77	0.202	2.05 (0.67, 6.28)	**0.184**	
High E2F group (N=34)	18	48.5	31		16	73.1	61	0.526	0.75 (0.30, 1.85)		
**Stage II**										
Low E2F group (N=20)	7	73.2	57		13	57.8	41	0.466	1.55 (0.47, 5.13)	**0.015**	
High E2F group (N=22)	11	18	18		11	NR	81	**0.012**	0.21 (0.06, 0.80)		
**Stage III/IV**										
Low E2F group (N=5)	3	22.8	0		2	22.5	≤50	0.455	0.43 (0.04, 4.20)	**0.74**	
High E2F group (N=18)	9	17.6	0		9	20.3	33	0.302	0.57 (0.20, 1.65)		

NR*: median survival time not reached within the study

HR**: hazard ratio

Int-P***: P value of interaction effect

Significant P values are in bold text

## DISCUSSION

We have explored the possibility that a measurement of E2F deregulation, an E2F score, could identify “under-treated” stage I patients likely to benefit from ACT and “over-treated” stage II patients unlikely to benefit from ACT. The resulting E2F score is based on 74 E2F-regulated genes and contains 18 internal control genes. The pathway-based E2F score originated using a targeted siRNA approach in cell lines and was validated using multiple cohorts and platforms. GeneGO analysis (Table S6) demonstrates that the predominant biological pathways downstream of E2F are proliferation and apoptosis, as expected. However, two of the genes in the E2F signature with high loading coefficients and independent predictive power are *LAMC2* (laminin C2) and *PLAUR (*plasminogen activator urokinase receptor), which are not directly tied to cell cycle or apoptosis. Serum levels of *LAMC2* have previously been shown to be prognostic in NSCLC [23] and *LAMC2* has been shown to drive metastatic potential of lung adenocarcinoma [24] in support of our findings. Likewise, components of the plasminogen activator pathway are thought to contribute to tissue remodeling in the context of tumorigenesis and *PLAUR* has specifically been implicated as a prognostic biomarker in NSCLC [25].

We have tested the E2F signature in two contexts, 1) as a prognostic biomarker in large number of patients and 2) as a predictive biomarker in patients randomized to surgery only or surgery plus ACT, in two clinical trials. As a prognostic biomarker, E2F-high patients demonstrate a significantly shorter survival than the low E2F-group when considering all patients, and more importantly when considering only stage I patients. Current clinical standard of care does not offer ACT treatment for the majority of stage I patients because this group is considered low-risk. However, the E2F signature was able to identify a subset of stage I patients with poor survival who may benefit from traditional ACT or from a therapy, such as treatment with a cyclin-dependent kinase inhibitor [26], that might counteract the aggressiveness of this E2F-driven disease. Although we cannot directly compare our findings to many other biomarkers [27, 28] in lung adenocarcinoma, such as histological subtype [13], our results indirectly support other investigations that have addressed the potential of proliferation-related genes as prognostic biomarkers. In particular, two products (Pervenio^TM^ from Life Technologies and MyPlan^TM^ from Myriad Genetics) have been described for estimating an early-stage NSCLC patient's likelihood of survival (prognosis) based upon gene expression signatures [16, 17]. Together these studies support the central role that E2F-driven proliferation plays in patient outcome and demonstrate that the E2F pathway is a relevant target to promote patient survival in lung adenocarcinomas.

As a predictive biomarker, the E2F score demonstrated a favorable ACT treatment effect in patients with high E2F when patients of all stages are considered. Further subgroup analysis indicates that the signature retains its predictive power in stage II patients. The benefit from cisplatin-based ACT in resected stage II lung adenocarcinoma patients as a whole has been established by several randomized clinical trials [6–12]. However, these studies also suggest that only a subset of these patients truly benefit from ACT [13]. Our data suggest that stage II patients can be classified as high-E2F patients who are likely to benefit from ACT and low-E2F patients who are unlikely to benefit. Although other gene signatures in lung cancer [15, 22, 29, 30] have been developed for this purpose, none have been validated in two independent randomized trials.

Given that the number of randomized clinical trials in early-staged lung cancer is limited, it was necessary for us to adapt the E2F signature for application to FF tissue to allow the use of the many available datasets, especially JBR10. However, in the future we foresee applying the E2F signature only to FFPE tissues. Informal surveys with practice groups in which we explain the usefulness our test suggest that the test is most likely to be utilized in high-risk stage I patients wishing to improve outcome. While we have not proven that the test is predictive in stage I patients at this point. We suggest that our prognostic data in stage I and predictive data in stage II justify a prospective clinical trial in which stage I lung adenocarcinoma patients with high-E2F scores would be randomized to surgery only or surgery plus ACT. Such a trial should also include other potential predictive markers [13, 27, 28]. Given that the number of patients examined in FFPE format is relatively small, we foresee further optimizing the data analysis component of the assay in a manner that will allow training on new data to be obtained in the future. In the NanoString format, the E2F assay is technically similar to the FDA-approved Prosigna^TM^ assay [2] for prognosis in breast cancer which is being adopted by many CLIA facilities where the assay is performed locally and the data analyzed centrally with fast turnaround.

In conclusion, we have identified and validated an E2F pathway-based scoring system that is a prognostic biomarker in stage I and a predictive biomarker in stage II lung adenocarcinoma patients. The NanoString-based E2F assay described herein represents a potential decision-support tool that would provide valuable information in the choice of ACT in early-stage lung adenocarcinoma patients.

## MATERIALS AND METHODS

### Study cohorts

Multiple datasets were used to develop and validate the E2F gene signature in this study (Figure 1 and Table S1). In particular, cells lines and RNAi were used to identify E2F regulated genes, and then two GEO datasets GSE18842 [31] (45 adjacent normal tissues and 46 tumors) and GSE19188 [32] (58 adjacent normal tissues and 87 tumors) were used to identify E2F-regulated genes that were measurably different between tumor and adjacent normal lung tissue. Four published datasets, that reported OS (overall survival) as primary outcome, were used to test the prognostic and/or predictive effects of the E2F signature. The MLOS dataset (Moffitt Lung Adenocarcinoma, Overall Survival) [33] includes 398 patients with available OS from the original 442 Moffitt lung adenocarcinoma patients with microarray gene expression data from fresh frozen (FF) RNA (Accession# GSE72094). The MCLA dataset (Molecular Classification of Lung Adenocarcinoma) [34] includes 442 lung adenocarcinoma patients with microarray gene expression data from FF RNA (Accession# GSE68465). The TCGA dataset (the Cancer Genome Atlas project, lung adenocarcinoma) [35, 36] includes 436 patients. TCGA utilized FF tissue and RNASeq was used to measure gene expression. The JBR10 dataset (National Cancer Institute of Canada Clinical Trials Group) [22] is a subset of the original JBR10 study representing 133 stage IB-II NSCLCs patients for which microarray data from FF tissue is available (Accession# GSE14814). Since this cohort is a mixture of lung adenocarcinomas and squamous cell carcinomas, we analyzed the data in two ways: JBR10 (adenocarcinomas and squamous) and JBR10.AD (adenocarcinoma only).

Three novel patient cohorts were used to test the prognostic/predictive potential of the E2F NanoString assay using OS and PFS (progression-free survival, reported only in the Supplementary Material). Detailed clinical characteristics of all seven cohorts are provided in Table S2 and S3. The LCBRN cohort (Lung Cancer Biospecimen Resource Network) includes 99 lung adenocarcinoma patients with RNA from FF tissue and was used to explore the prognostic effect. The MLCom cohort (Moffitt Lung Adenocarcinoma, Complete) [37] was used to test the prognostic effect and as the training dataset for RNA from FFPE. This cohort includes 150 lung adenocarcinoma patients and is referred to as “complete” since NanoString results were acquired and detailed medical chart review was performed. Although previously reported [37] this is the first time this cohort has been explored with respect to survival. The NATCH cohort [(Neo)-Adjuvant Taxol/Carboplatin Hope] [7] was used for both prognostic and predictive effects. The NATCH trial was a randomized trial including three arms: 1) surgery only, 2) surgery followed by paclitaxel-carboplatin ACT and 3) paclitaxel-carboplatin followed by surgery. Herein, NATCH includes a 74-patient lung adenocarcinoma subset from Arms 1 and 2 for which FFPE blocks were available. An additional cohort, referred to as MLTO (Moffitt Lung Adenocarcinoma Technical Optimization) consisted of 36 lung adenocarcinoma patients for which we obtained matching FF and FFPE tissue for direct comparison. It does not overlap with any other cohorts and was used only for methods optimization.

### RNA preparation from tissue samples

RNA from Moffitt patients was acquired through Moffitt's Tissue Core Facility, an established honest broker system under the supervision of USF's Institutional Review Board and Moffitt's Scientific Review Committee. Tissue blocks were reviewed by a certified staff pathologist for confirmation of a diagnosis of adenocarcinoma, and percent malignancy, cellularity, stroma, and immune infiltration. Three 10-μm and one 5-μm sections of each FFPE block were cut. The 5-μm section was stained with hematoxylin and eosin (H&E) and the staff pathologist marked approximate tumor margins using the H&E stained slide. The tumor regions of the three 10-μm slides were excised and subjected to RNA extraction using Qiagen's RNeasy FFPE kit (as previously reported) [38, 39].

### NanoString experiments

NanoString Assays were performed with 150-ng aliquots of RNA using the NanoString nCounter Analysis system (NanoString Technologies, Seattle, WA). Generic codesets (Table S4) were obtained directly from NanoString Technologies and gene-specific oligonucleotides were obtained from IDT (Integrated DNA Technologies, Coralville, Iowa). After codeset hybridization overnight, the samples were washed and immobilized to a cartridge using the NanoString nCounter Prep Station. Cartridges were scanned in the nCounter Digital Analyzer at 555 fields of view for the maximum level of sensitivity. Ultimately, 18 highly invariant genes were selected to serve as internal controls for normalization between samples and 74 genes represented the E2F pathway (Table S5).

### Derivation and validation of an E2F scoring system

The overall E2F scoring system was generated by principal component analysis (PCA) [15] with the first principal component (PC1) of the E2F-regulated genes representing the E2F score. First, PCA was performed to derive PC1 in the FF and FFPE training cohorts. Next, PC1 from the training cohort was used to calculate an E2F score in the validation cohorts. Utilization of the median E2F score as the cutoff was justified by systematically comparing various cutoffs. To validate the prognostic and predictive effects of the E2F signature, each platform used the corresponding training cohort to classify patients into low or high E2F groups. The high and low groups in each cohort (or combined cohort) were then used for subsequent analyses. For validation of the prognostic effect, the E2F signature was analyzed to identify survival differences between the high and low E2F groups using the log-rank test or by Cox proportional hazards model for covariate adjustment. For validation of the predictive E2F signature, the Cox proportional hazards model was used to identify any differential treatment effect by testing interaction effect while the log-rank test was used to test the treatment effect (ACT versus non-ACT) in each risk group (low or high E2F).

### Statistical analysis

Microarray data processing in patient samples included IRON [40] and COMBAT [41] methods for normalization. NanoStringNorm R package [42] was used to NanoString data. For optimization of the E2F signature in NanoString, Spearman and Pearson correlation analysis was used to remove poorly correlated genes (between FF and FFPE) and to evaluate effects by platforms (microarray and NanoString) and by tissue type (FF and FFPE) using the MLTO cohort. To validate the E2F signature, we employed a training and validation scheme. Specifically, we used the MLOS cohort and the MLCom cohort as the training set for FF and FFPE tissues, respectively. The validation cohorts were MCLA, TCGA, LCBRN and JBR10.AD for the FF platform and the NATCH cohort for the FFPE platform. PCA was used to derive the E2F scoring system as described previously. Log-rank test and Cox proportional hazards model were used for survival analysis. Proportional hazards assumption was performed for the Cox model analyses. OS was defined as from date of surgery (or randomization for JBR10 and NATCH) to date of death or last date of follow-up for those patients still alive. PFS was defined as from date of surgery to date of recurrence, progression, or death. Those alive with no evidence of disease at last follow-up were censored. When information was unknown or unavailable, analyses were performed on the largest possible subset. *Sample size justification:* For the prognostic effect, we used non-ACT patients from the 5 combined cohorts (MLOS, MCLA, TCGA, JBR10.AD, and LCBRN: 1065 non-ACT patients with 287 events) in fresh frozen platform and 2 cohorts (MLCom and NATCH: *N* = 141 non-ACT patients with 68 events) in FFPE platform. Assuming 50% prevalence of high E2F in each combined cohort, the sample size of 1065 non-ACT patients with 287 events will have 80% power to detect a hazard ratio of 1.4 (HR: high vs. low E2F) with two-sided 5% type I error. The power for the sample size of 141 non-ACT patients with 68 events will be 80% to detect a HR of 2.03. For the predictive effect, we combined two randomized trials (JBR10.AD and NATCH: *N* = 145 with 77 events, an overall 5-year survival rate of 53%, and 50% patients in ACT). With this information and assumption of 50% prevalence of high E2F in the ACT and non-ACT, this sample size will have 80% power to detect a hazard ratio of 0.26 (HR in high E2F/HR in low E2F, assuming a 5-year survival rate of 66% and 20% for low and high E2F, respectively, in the control group, and 66% for both low and high E2F in the treatment group) with a two-sided 5% type I error. Power calculation is based on the functions, by R package powerSurvEpi [43] PowerPredictiveBiomarker.shiny (for the predictive effect) by R package PowerPredictiveBiomarker [44] (github.com/dungtsa/PowerPredictiveBiomarker).

## SUPPLEMENTARY FIGURES AND TABLES


