# Supplementary material for: Early2 factor (E2F) deregulation is a prognostic and predictive biomarker in lung adenocarcinoma
Source: Oncotarget. 2016 Oct 14;7(50):82254–65. doi: 10.18632/oncotarget.12672 (PMC5347689; doi:10.18632/oncotarget.12672)
Supplement: Supplementary file 1 [file oncotarget-07-82254-s001.docx]

Early2 factor (E2F) deregulation is a prognostic and predictive biomarker in lung adenocarcinoma

Supplementary Material

Contents

[Methods 3](#_Toc462774110)

[Study cohorts (Table S1) 3](#_Toc462774111)

[Published tumor/normal gene expression datasets without survival information 4](#_Toc462774112)

[Published patient cohorts (Table S2) 4](#_Toc462774113)

[Our new patient cohorts (Table S3) 6](#_Toc462774114)

[siRNA in cell lines 8](#_Toc462774115)

[RNA preparation 8](#_Toc462774116)

[NanoString experiments (Table S4 and Table S5) 8](#_Toc462774117)

[Statistical analysis 19](#_Toc462774118)

[Microarray analysis in siRNA-treated knockdown and control cell lines 20](#_Toc462774119)

[Data processing of microarray platform in patient data. 20](#_Toc462774120)

[Data processing of NanoString platform. 20](#_Toc462774121)

[E2F signature optimization in NanoString platform. 21](#_Toc462774122)

[E2F scoring system 21](#_Toc462774123)

[Median-threshold of E2F score for risk classification. 22](#_Toc462774124)

[Validation of the prognostic E2F signature. 22](#_Toc462774125)

[Validation of the predictive E2F signature 23](#_Toc462774126)

[Results 23](#_Toc462774127)

[A siRNA-derived E2F signature is representative of cell cycle (Table S6 and Figure S1) 23](#_Toc462774128)

[Translation of the E2F signature to a NanoString format 25](#_Toc462774129)

[a. Side-by-side comparison of individual gene expression between FF and FFPE to eliminate poor correlated genes (Table S7 and Figure S2). 25](#_Toc462774130)

[b. Comparison of the PC1 scores derived from the 74 gene signature and the original 106 genes (Figure S3 and S4). 29](#_Toc462774131)

[c. Correlation analysis of PC1 Score among FF in microarray and FF and FFPE in NanoString (Figure S5). 31](#_Toc462774132)

[E2F scoring system 32](#_Toc462774133)

[a. Percentage of total variation for PC1 (Fig S6) 33](#_Toc462774134)

[b. Correlation of gene weights between the two platforms (Table S8 and Fig S7) 33](#_Toc462774135)

[Post-hoc evaluation of E2F median-cutoff classification from training to validation cohorts (Table S9, S10, S11, S12 and Figure S8-S9) 36](#_Toc462774136)

[Analysis of prognostic effects for Non-ACT patients in each cohort (Table S13) 45](#_Toc462774137)

[Multivariate analysis of prognostic effects (Table S14) 47](#_Toc462774138)

[Evaluation of prognostic effect for the combined cohorts from the two randomized clinical trials (JBR10.NSCLC + NATCH or JBR10.AD + NATCH) (Table S15-S16) 53](#_Toc462774139)

[Evaluation of predictive effect for PFS in the NATCH cohort and DSS in the JBR10 cohort (Table S17) 55](#_Toc462774140)

[Predictive effect (OS) after adjustment for tissue type, FF and FFPE (Table S18) 56](#_Toc462774141)

[Multivariate analysis of predictive effect (Table S19) 58](#_Toc462774142)

[Proportional hazard assumption test (Table S20) 67](#_Toc462774143)

[ROC curves showing added value of E2F gene signature (Table S21) 74](#_Toc462774144)

[Median survival time of published JBR10 and NATCH trials 74](#_Toc462774145)

[1). JBR10 trial 74](#_Toc462774146)

[2). NATCH trial 75](#_Toc462774147)

[References: 75](#_Toc462774148)

# Methods

## Study cohorts (Table S1)

A series of cohorts/datasets were used to develop and validate the E2F gene signature; including, cell lines (H1299/A549) tumor/normal datasets (GSE18842[^1^](#_ENREF_1) and GSE19188[^2^](#_ENREF_2)), one cohort of patient samples for technical validation (MLTO) and seven cohorts of patient samples for training or validation of prognostic and/or predictive effects (MLCom, NATCH[^3^](#_ENREF_3), LCBRN, MLOS[^4^](#_ENREF_4), MCLA[^5^](#_ENREF_5), TCGA[^6^](#_ENREF_6)^,^ [^7^](#_ENREF_7), and JBR10[^8^](#_ENREF_8)). Five new cohorts were generated by our study including H1299 and A549 cell lines with control, E2F1, E2F3a, E2F3b, E2F3a/b, E2F4, and Rb knock-down, respectively, in microarray platform and MLTO, MLCom, NATCH, and LCBRN in NanoString platform. Six cohorts were from the public data including GSE18842[^1^](#_ENREF_1), GSE19188[^2^](#_ENREF_2), MLOS[^4^](#_ENREF_4), MCLA[^5^](#_ENREF_5), and JBR10[^8^](#_ENREF_8) in microarray platform and TCGA[^6^](#_ENREF_6)^,^ [^7^](#_ENREF_7) in RNA-Seq platform. Table S1 summarizes the cohorts used in this study.

Table S1: Summary of the study cohorts/datasets for the E2F signature development and validation

| Cohort/Dataset | Accession | Tissue type | Platform | Prognostic effect4 | Predictive effect w/ACT5 | Technical validation |
| --- | --- | --- | --- | --- | --- | --- |
| H1299/A549 | this work | Cells | Microarray |  |  | X |
| Tumor/normal 1 | GSE18842 | FF | Microarray |  |  | X |
| Tumor/normal 2 | GSE19188 | FF | Microarray |  |  | X |
| MTO | this work | FF/FFPE | MicroarrayNanoString |  |  | X |
| MLOS | GSE72094 | FF | Microarray | X |  |  |
| MCLA | GSE68465 | FF | Microarray | X |  |  |
| TCGA | TCGA | FF | RNAseq | X |  |  |
| JBR.10 | GSE14814 | FF | Microarray | X | X |  |
| LCBRN | this work | FF | NanoString | X |  |  |
| MLCOM2 | this work | FFPE | NanoString | X |  |  |
| NATCH | this work | FFPE | NanoString | X | X |  |

^1^PCA training set for microarray and RNAseq on FF RNA
^2^PCA training set for NanoString on FFPE RNA
^3^TCGA LUAD data portal: <https://tcga-data.nci.nih.gov/>
^4^The prognostic effect compares survival of patients with a high E2F signature score and those with a low score without regard to treatment
^5^The predictive effect compares survival within each treatment arm of a randomized trial (treated with ACT versus not treated) and is present if the E2F score significantly affects survival in one arm and not the other

### Published tumor/normal gene expression datasets without survival information

1. GSE18842. The cohort included 45 adjacent normal tissues and 46 tumors. The cohort was used to filter out the E2F associated genes from the E2F cell line experiments.
2. GSE19188. The cohort included 58 adjacent normal tissues and 87 tumors. The cohort was used to filter out the E2F associated genes from the E2F cell line experiments.

### Published patient cohorts (Table S2)

Four published datasets, described below, were used to test the prognostic and/or predictive effects of the E2F signature in microarray or RNA-Seq platform. These four datasets reported OS as primary outcome variable. Additional clinical characteristics of these four datasets are tabulated in Table S2.

1. The MLOS cohort (Moffitt LUAD, Overall Survival) includes 398 Moffitt LUAD patients with Affymetrix-based microarray gene expression data from fresh frozen (FF) RNA (GSE72094). The median follow-up time was 28.6 months. There were 113 (28.4%) deaths. The number of Non-ACT patients was 300 (75.4%). There were 254 (63.8%) patients with stage I, 67 (16.8%) patients with stage II, and 72 (18.1%) patients with stage III/IV. This cohort was used as a training cohort for the microarray platform.
2. The MCLA cohort (Molecular Classification of Lung Adenocarcinoma) includes 442 LUAD patients with Affymetrix-based microarray gene expression data from FF RNA (Accession# GSE68465). The median follow-up time was 66 months. There were 236 (53.4%) deaths. The number of Non-ACT patients was 233 (52.7%). There were 276 (62.4%) patients with stage I, 95 (21.5%) patients with stage II, and 68 (15.4%) patients with stage III/IV. This cohort was used as a validation cohort to evaluate the prognostic effect in the microarray platform.
3. The TCGA cohort (the Cancer Genome Atlas project, LUAD) includes 436 LUAD patients. TCGA utilized FF tissue for RNA and RNASeq was used to measure gene expression. The median follow-up time was 5.1 months. There were 111 (25.5%) deaths. The adjuvant chemotherapy information was unknown. There were 235 (53.9%) patients with stage I, 103 (23.6%) patients with stage II, and 97 (22.2%) patients with stage III/IV. This cohort was used as a validation cohort to evaluate the prognostic effect in the microarray platform.
4. The JBR10 cohort (National Cancer Institute of Canada Clinical Trials Group) a subset of the original JBR10 study representing 133 Stage IB-II NSCLCs patients (62 surgery only and 71 surgery plus cisplatin/vinorelbine ACT) for which microarray data from FF tissue is available (Accession# GSE14814). Two sets of the cohort were analyzed: JBR10.NCSLC (AD+SQ) and JBR10.AD (AD only). The median follow-up time was 82.4 months. For JBR10.NCSLC, there were 60 (45.1%) deaths. The number of Non-ACT patients was 62 (46.6%). There were 73 (54.9%) patients with stage I and 60 (45.1%) patients with stage II. For JBR10.AD, there were 35 (49.3%) deaths. The number of Non-ACT patients was 32 (45.1%). There were 42 (59.2%) patients with stage I and 29 (40.8%) patients with stage II. Both cohorts were used as a validation cohort to evaluate both the prognostic and predictive effects in the microarray platform.

Table S2: Demographics of the published cohorts for E2F signature evaluation

|  | MLOS (N=398) | MCLA (N=442) | TCGA (N=436) | JBR.10 (N=133) |
| --- | --- | --- | --- | --- |
| Age (years, mean[SD]) | 69.4 (9.4) | 64.4 (10.1) | 65.7 (9.7) | 60.8 (9) |
| Follow-up time (months, median[IQR]) | 28.6 (22.9-34.7) | 66 (44-87.8) | 5.1 (1-16) | 82.4 (64.6-95.3) |
| Adjuvant Chemotherapy |  |  |  |  |
| Adjuvant Chemotherapy ACT | 73 (18.3%) | 89 (20.1%) |  | 71 (53.4%) |
| Adjuvant Chemotherapy No ACT | 300 (75.4%) | 233 (52.7%) |  | 62 (46.6%) |
| Adjuvant Chemotherapy z Unknown | 25 (6.3%) | 120 (27.1%) | 436 (100%) |  |
| Gender |  |  |  |  |
| Gender Female | 222 (55.8%) | 219 (49.5%) | 237 (54.4%) | 42 (31.6%) |
| Gender Male | 176 (44.2%) | 223 (50.5%) | 199 (45.6%) | 91 (68.4%) |
| Smoking history |  |  |  |  |
| Smoking history, No | 31 (7.8%) | 49 (11.1%) | 62 (14.2%) |  |
| Smoking history, Yes | 300 (75.4%) | 300 (67.9%) | 362 (83%) |  |
| Smoking history, Unknown | 67 (16.8%) | 93 (21%) | 12 (2.8%) | 133 (100%) |
| Stage |  |  |  |  |
| Stage I | 254 (63.8%) | 276 (62.4%) | 235 (53.9%) | 73 (54.9%) |
| Stage II | 67 (16.8%) | 95 (21.5%) | 103 (23.6%) | 60 (45.1%) |
| Stage III/IV | 72 (18.1%) | 68 (15.4%) | 97 (22.2%) |  |
| Stage, Unknown | 5 (1.3%) | 3 (0.7%) | 1 (0.2%) |  |
| Vital Status |  |  |  |  |
| Vital Status Alive | 285 (71.6%) | 206 (46.6%) | 325 (74.5%) | 73 (54.9%) |
| Vital Status Dead | 113 (28.4%) | 236 (53.4%) | 111 (25.5%) | 60 (45.1%) |

Detailed sub-stage information (A or B) was not available for all patients Abbreviations: IQR: inter-quartile range (1st - 3rd quartile); SD: Standard Deviation

### Our new patient cohorts (Table S3)

Four cohorts used the NanoString assay to generate E2F gene expression, including one patient cohort (MLTO) for technical validation and three novel patient cohorts (MLCom, NATCH, LCBRN) for testing the prognostic and/or predictive effects of the E2F NanoString assay with OS as the primary outcome event. The clinical characteristics of MLCom, NATCH, and LCBRN cohorts are described below and in Table S3. When information was unknown or unavailable, analyses were performed on the largest possible subset.

1. The MLTO cohort (Moffitt LUAD for technical optimization). This cohort included 36 Moffitt LUAD patients with available matching RNA from FF tissue and from FFPE blocks. The paired data were used to determine the best codesets for the final NanoString assay. In addition, the cohort had microarray data from FF tissues to evaluate effect of platforms and tissue types.
2. The MLCom cohort (Moffitt LUAD, complete). This cohort used FFPE tissues from 150 LUAD patients with detailed medical chart review to obtain PFS and OS and was referred to as "complete". The median follow-up time was 55.3 months. There were 76 (50.7%) deaths and 86 (57.3%) patients with disease progression. The number of Non-ACT patients was 101 (67.3%). There were 84 (56%) patients with stage I, 25 (16.7%) patients with stage II, and 41 (27.3%) patients with stage III/IV. This cohort was used as a training cohort for the NanoString platform.
3. The NATCH cohort [(Neo)-Adjuvant Taxol/Carboplatin Hope]^3^. This cohort included 74 LUAD patients (FFPE blocks), a subset of the NATCH trial which was a randomized trial with three arms: a) surgery only, b) surgery followed by paclitaxel-carboplatin and c) paclitaxel-carboplatin followed by surgery. Both PFS and OS were available for analysis in this cohort. The median follow-up time was 64 months. There were 42 (56.8%)deaths and 48 (64.9%) patients with disease progression. The number of ACT and Non-ACT patients was 34 (45.9%) and 40 (54.1%), respectively. There were 38 (51.4%) patients with stage I, 13 (17.6%) patients with stage II, and 23 (31.1%) patients with stage III/IV. This cohort was used as a validation cohort to evaluate both prognostic and predictive effects in the NanoString platform.
4. The LCBRN cohort (Lung Cancer Biospecimen Resource Network, <http://www.lcbrn.org/>). This cohort included 99 LUAD patients with RNA from FF tissue. Both PFS and OS were available for analysis in this cohort. The median follow-up time was 24 months. There were 23 (23.2%) deaths and 40 (40.4%) patients with disease progression. The number of Non-ACT patients was 64 (64.6%). There were 55 (55.6%) patients with stage I, 31 (31.3%) patients with stage II, and 12 (12.1%) patients with stage III/IV . This cohort was used as a validation cohort to evaluate the prognostic effect using the NanoString platform.

Table S3: Demographics of the novel cohorts for E2F signature evaluation

|  | MLCom (N=150) | NATCH (N=74) | LCBRN (N=99) |
| --- | --- | --- | --- |
| Age (years, mean[SD]) | 69.2 (8.2) |  | 66.8 (10.4) |
| Follow-up time (months, median[IQR]) | 55.3 (43.4-66.3) | 64 (43-73.7) | 24 (19.8-30) |
| Adjuvant Chemotherapy |  |  |  |
| Adjuvant Chemotherapy ACT | 41 (27.3%) | 34 (45.9%) | 35 (35.4%) |
| Adjuvant Chemotherapy No ACT | 101 (67.3%) | 40 (54.1%) | 64 (64.6%) |
| Adjuvant Chemotherapy z Unknown | 8 (5.3%) |  |  |
| Gender |  |  |  |
| Gender Female | 76 (50.7%) | 20 (27%) | 47 (47.5%) |
| Gender Male | 74 (49.3%) | 54 (73%) | 52 (52.5%) |
| PFS event |  |  |  |
| PFS event 0 | 58 (38.7%) | 26 (35.1%) | 59 (59.6%) |
| PFS event 1 | 86 (57.3%) | 48 (64.9%) | 40 (40.4%) |
| PFS event z Unknown | 6 (4%) |  |  |
| Smoking history |  |  |  |
| Smoking history No | 7 (4.7%) |  | 12 (12.1%) |
| Smoking history, Yes | 120 (80%) |  | 87 (87.9%) |
| Smoking history, Unknown | 23 (15.3%) | 74 (100%) |  |
| Stage |  |  |  |
| Stage I | 84 (56%) | 38 (51.4%) | 55 (55.6%) |
| Stage II | 25 (16.7%) | 13 (17.6%) | 31 (31.3%) |
| Stage III/IV | 41 (27.3%) | 23 (31.1%) | 12 (12.1%) |
| Stage z Unknown |  |  | 1 (1%) |
| Vital Status |  |  |  |
| Vital Status Alive | 74 (49.3%) | 32 (43.2%) | 76 (76.8%) |
| Vital Status Dead | 76 (50.7%) | 42 (56.8%) | 23 (23.2%) |

Abbreviations: IQR: inter-quartile range (1st - 3rd quartile); SD: Standard Deviation

## siRNA in cell lines

An RNAi approach was used to independently deplete lung cancer cell lines of E2F1, E2F3A, E2F3B, both -3A and -3B, E2F4, and pRb (Figure S1A). A549 and H1299 cell lines were obtained from ATCC and cultured at 37°C with 5% CO2 in RPMI with 10% FBS (no antibiotics). Cells were plated at ~50% confluency, then transfected with siRNA (Dharmacon) using Lipofectamine 2000 per manufacturer instructions. The siRNA used were siGENOME Non-Targeting siRNA #2, E2F1 ON-TARGETplus SMARTpool siRNA, E2F4 ON-TARGETplus SMARTpool siRNA, RB1 ON-TARGETplus SMARTpool siRNA, and E2F3a, E2F3b, and E2F3a+b sequences from^7^. Cells were harvested 36 hours following transfection. Approximately 30 $\mu g$ of whole cell lysates were resolved in each lane of 10% SDS-PAGE gels. Protein was wet-transferred onto PVDF membranes for two hours at 100 volts on ice. Membranes were blocked in 5% milk for 30 minutes at room temperature, rinsed for 5 minutes in water, then placed in primary antibodies diluted 1:1000 in PBS. Antibodies used for immunoblotting were as follows: E2F1 (C-20, sc-193, Santa Cruz), E2F3 (C-18, sc-878, Santa Cruz), monoclonal $\beta$-actin (clone AC-15, cat no: A5441, Sigma), E2F4 (c-108, sc-512, Santa Cruz) and Rb (Ab-1, #OP28, Calbiochem). Proteins were detected using horseradish-peroxidase-conjugated secondary antibodies and enhanced chemiluminescence (ECL) purchased from Amersham or Thermo Scientific.

## RNA preparation

RNA from Moffitt patients was acquired through Moffitt's Tissue Core Facility, an established honest broker system under the supervision of USF's Institutional Review Board and Moffitt's Scientific Review Committee. All tissue blocks were reviewed by a certified staff pathologist for confirmation of a diagnosis of adenocarcinoma, and percent malignancy, cellularity, stroma, and immune infiltration. Three 10-$\mu m$ and one 5-$\mu m$ sections of each FFPE block were cut. The 5-$\mu m$ section was stained with hematoxylin and eosin (H&E) and the staff pathologist marked approximate tumor margins using the H&E stained slide. The tumor regions of the three 10-??m slides were excised and subjected to RNA extraction using Qiagen's RNeasy FFPE kit (as previously reported)^4,5^. In the developmental stages of the work, samples were quality-controlled using an Agilent bioanalyzer, a step later deemed a waste of sample since the internal controls genes were the best way to assess sample quality.

## NanoString experiments (Table S4 and Table S5)

NanoString Assays were performed with 150-ng aliquots of RNA using the NanoString nCounter Analysis system (NanoString Technologies, Seattle, WA). The CLIA-ready labeled codesets were obtained directly from NanoString and gene-specific oligonucleotides were obtained from IDT (Integrated DNA Technologies, Coralville, Iowa). The codesets were listed in Table S4. After codeset hybridization overnight, the samples were washed and immobilized to a cartridge using the NanoString nCounter Prep Station. Cartridges were scanned in the nCounter Digital Analyzer at 555 fields of view for the maximum level of sensitivity. Eighteen highly invariant genes were selected to serve as internal controls for normalization between samples (Supplementary Table S5 for a list of internal control genes).

Table S4 Gene Specific Primers for the E2F NanoString Elements Assay, Housekeeping genes are in bold

| Probe | Gene | Sequence |
| --- | --- | --- |
| A | ABAT | GGGCGAGGGAGCCAAAAGTCAGACCTCAAGTTGCCCTGGGAAACATGATGCCTCAAGACCTAAGCGACAGCGTGACCTTGTTTCA |
| A | ABCC6 | GCCACCAGAGTGTGGACAGCAAACACCACCAGTGCGACCAGAAATGTAGACATCCTCTTCTTTTCTTGGTGTTGAGAAGATGCTC |
| A | ACOX2 | AGCTAAACTCCGGGTAACTGTGGATGATGCTCTCAACTTTCCTGCGGAGTCACAATTCTGCGGGTTAGCAGGAAGGTTAGGGAAC |
| A | AGPAT9 | TAAAAGGATGAAGCCGAGAACCAGCGTCAGCCAGGTGGAAAGGATCTTCCCTGTTGAGATTATTGAGCTTCATCATGACCAGAAG |
| A | ANXA1 | AGATCTCTCTTCAGTTCCTCTCTGTAGACCCTGTTAATGTCTCTGATTTCCGAACCTAACTCCTCGCTACATTCCTATTGTTTTC |
| A | ARL14 | TCATGTGGCTCTTCACAAATCCAGTTAATTTCCTGAACCCCTGGGCCAGCCAACAGCCACTTTTTTTCCAAATTTTGCAAGAGCC |
| A | BDH2 | CCCAGTCTTTCTCCTCACAATCCAGGACAGTTCCATGATGGACAAAACCACAGATAAGGTTGTTATTGTGGAGGATGTTACTACA |
| A | BIRC5 | TCTCCTCATCCACCTGAAGTTCACCCCGTTTCCCCAATGACTTAGAATGGCTTCCTTCCTGTGTTCCAGCTACAAACTTAGAAAC |
| A | BLM | ACTCTTACCACCTCCAGTCGGCATCAGGATAAAACAGTCTTCACCAAGCACATAAAATTGGTTTTGCCTTTCAGCAATTCAACTT |
| A | BUB1B | CCTTTGCTCTTTAGTTCAGCTAGTGTGCTTCGTTGTGGTACAGAAGACTCCTGGTCAAGACTTGCATGAGGACCCGCAAATTCCT |
| A | CCNE2 | TCAGGTGTATAAAACCTCGCCAGACCAGCTACCGCTCGGCTGAGGCTGTTAAAGCTGTAGCAACTCTTCCACGA |
| A | CDC6 | GACTTTGCGGGCACAGAATTGAACTGCAGCATTGTCCAGAACCTGATCTCCCACGCGATGACGTTCGTCAAGAGTCGCATAATCT |
| A | CDCA4 | GCCGTGGTCGTAACTAACACCACATTCCCATTTTGTTTGCTGGATAATTTCACAAGAATCCCTGCTAGCTGAAGGAGGGTCAAAC |
| A | CDK6 | TCAAGTCGCGGGCCTTGAACACCTTCCCATAGGCGCTTACAGATCGTGTGCTCATGACTTCCACAGACGT |
| A | CHST11 | TATCAGGGGTTTGCTAATTGTCGAGCTGGATGCTCTATGTCACATAAAGCCCCGAAGCAATACTGTCGTCACTCTGTATGTCCGT |
| A | CKS1B | GTTCATGGATCATATAATGGACCCATCCCTGACTCTGCTGAACGCCAAGACCGGGAATCGGCATTTCGCATTCTTAGGATCTAAA |
| A | CPM | AACCCACAGGTTTCTACCTTTCACAGATTTCCCAATACTGTGTAAGTGAGCATTCGCAACCATGTGAAGTAATGTGAGCGTACTT |
| A | CYBRD1 | TTAAACTTTTAGGACTGCTTCCCGCTAGTTGTGAAATCATTGTCTCCCAGCACCAGTTAGCGTGGCGTATACCATGTTGTTAACA |
| A | CYP1B1 | CTTGTTGATGAGGCCATCCTTGTCCAAGAATCGAGCTGGATCAAAGTTCTCCTGAATCAATAGAACAATATCAGTTATGGCGGTG |
| A | DOCK4 | AAAAGTAAAGAACACTCGGTCTGGACGACCACCGCCGACTCCGAAGCAGGCGGTTGTTAATATGACAGGCCGCTAAAGACGTTCT |
| A | DTL | ACTTAGAAAAGGCAACTGACTTGAGGCTGCATTGATGACCTTTGCATGTTCTATCAGCTAATAGGGTCGGCTCAACAGTGTATCC |
| A | EVI5 | CCTGGTCCAGATCATTCAGATTTCTTGATGTGAGGGTCAAAGCAATAATGCTAGCCCAGATCCTACGAGATGAGCTACGTAACTA |
| A | FIGNL1 | CGGTCTCGATCATGAGCTGAGTTAGGTAATGAGTCACTCTCTTGAGAACTCACGATCTGTATTTTGCACCTTTCGCTATGCTGAG |
| A | FN1 | AACAATGCACTGATCTCGAAGCTGCGAGTAGGCAATGCATGTCCATTCCCCTGTGTCCGTCTATACGCATACTGGTCCACATATA |
| A | GBP2 | GTTCCAGGGTGAAATCTCTGAGAGTCCACACAAATGCTGGAAAAAAGCTCCGCTCATTTTGAACATACGATTGCGATTACGGAAA |
| A | GINS1 | AAGCGGTCATACAGGTATGCTACAGTGCAGCGTCGATTTCTTAACAGAGACCTATGCATCATGTGCCTCACTAGGACATCATGCT |
| A | GLIPR1 | TGGCTGTTGGTTTCACCTCTGATCGGAACTTGTTATGGATTCGAACGCAGCCTAAATTGGGAAAAAAGGTTTTAGCTATTGATGG |
| A | GNG2 | AAAAGCATTTCCCAGCTCATGGTGAATCCTTAACTCTCCTGCTTGGTGACCTTCAGTTAAAGGCTATCTTGCTCCGCTCGTTCTC |
| A | HAUS6 | GCCTCACAACCTCCGGGAAATTGAGTTTCTAACGGTATAGTGCGGCCACCCTCTGTGAACTGTCATCGGTCCGATCAATTAGTCT |
| A | IDS | CCAAAGCACTCAAGAGGCGGCCGACCTGTGTATCCAAATATGACACAGAGCCATCCACTTTCATGGAAACAATAAGAGCAGGGAA |
| A | IMPA2 | CTCTTGTCGAACAGCAAATCCAATGCTAACCGCCACAGTCGGGAATCTGTCACAAACTCACTACTACCAACAACCTCACCAAAAA |
| A | IRS1 | GAGTACCAGCAACTTCCAGTTTTAAATCGGTAGTTTGATGCCACCATCAACTCATGTCCTCTGTTAATCCAGCCTGAATATGCCA |
| A | ISG20 | ACAGCACAGCACCGTGGACGTTCACGAGGCTGCAACGAGCCAGAAATGTCACTCCCATGGTGGCTGATATAGAAA |
| A | ITGB8 | CCTGAGACAAATTGTGAGGGTGAAGGCATTGCATACAATTCCAGTTTTCCCATGTCGAACCTTGGATAGGAGCGACCGATTACGT |
| A | KIF4A | CAGAATGCTCTGAACAATATTTTAGATCCTCGATGTCCATGCTTTGCTCCCTTAGGCTACCAAATGAATTTAAAGCCAGCTGAAA |
| A | LAMC2 | AGGAGGCGGAGACTGTGCTGATAAGACCTATCTGCTTCAATTTCCGCTTGCCTGCATTCTCATGGAAATGCAATGGATTCATTCC |
| A | LAT2 | CCCCAGCTCTTCCCATGAGACAGTCACAACACTGTTTAATCAGCCTGTTGCAGTATCACGTAAATACCTACTTCGATA |
| A | LMNB1 | CCAAGCGCGTTTCATGCTTCCTTCTGGTCTCGTTAATCTCCTCTTCATACCCTTACGACTTCACTGCAATTGACGATTCAGTTAA |
| A | MAP2 | ACAGATTTGTAACAGTGTGTTGGAACCTCGGAATCCCAGCATACAGAGTACTTTCGCCACCCATATAAACCCCACTTCGTCCTCA |
| A | MCM10 | AGCTCATCAAATGCGTCGGGCTCGCCATTTTCCCGCGTCAAGAAGTTATTCCTACATATATAGGAAAAGGGAAGGTAGAAGAGCT |
| A | MCM2 | CAAGTAAGACACACTGGCAAGGCATCCCACCCAAAGGTGAGAAGCACAAACCTTCTGGAATTTCTTCCTTTGATTTTGCCATTTT |
| A | MDFIC | TCATCACATCCGGATCGGGAAGGCCTCGAACTCTTGGCTAGCGGCCAAGGCCTAGCCTAAAGGTTCTTGCAGAGCAACAT |
| A | MEX3D | CTGCCCCCTCCGGCCGCGCACGGTTGATGTACAAAAGCATAAATGGTTAGCTTGAGTTATACGGAACTTCGCAAAAGTATTCCCT |
| A | MYB | CATTGTCTTCATCTTCTGATGCTGGTGCCATTAAAACGGAGCTTGTAAGACTCTAACCTGCATACATATGGCATTTAGTTGTTCA |
| A | NCAPD2 | TGGGAGTCGCAGAAAGTGGCACTGATCATGCAGAACTTGCCAAGGGCAAGCCTTCTTGAAGACCTATGTAAAGAAACGGGTCACT |
| A | NCAPG | ATTGTCATGTACTGTTAAGGCCTGATAATCTTGGGAAGTCTTGGCCTGAGCCATACGCATGACTACATTACAACGGGCCAGGAAG |
| A | NDC80 | AGGGACCGAATTTCTGTAAACCGTGCAAGTATCTGCTAGACAGGACAGCGCATGAACGTGTCGTGTTATGCAGCGGTATGTCGTG |
| A | NRP1 | TAGAGCTGTACAATCCTCAGCCCGTCTTGGAGAAAAGAAAGCAGCGAGGCCGCCCTGGAACAACGGTTATATTCTCTGGCAGAAC |
| A | PLAUR | CTGGTGATCTTCAAGCCAGTCCGATAGCTCAGGGTCCGAGATACGTCTTACCGCACATAGGTAACCTATT |
| A | PLK1 | TGAGGTCTCGATGAATAACTCGGTTTCGGTGCAGGTACTGGCAGCCAAGCGCTGGGTTAAATAGAGAAATGGCTACTCATTTAG |
| A | PLSCR4 | AAGGGTTAGGGATAGAAGCCAGGTTATTTTATGCCAACAAAGTCTGCTCTCTAGCTCAATTGCCATTGCTGGAATAGTGTCGGTA |
| A | PRMT3 | CCAGTTCCACACCCAACATCCAAAACTACCTTGTCTTTGAAGATATGTGGCGGCTATGATTCACCACCGTGTAGGAGTTATGCGC |
| A | PTP4A1 | ATCGTAAAATGCAATCCTTCTAGGGCTGGAACCGCGTCTCAGTGTCGAGCCGATATTTTGCTGATGTAACTGTTACTGACCTTGC |
| A | QKI | GTTGTTTGGCTGTAAGTCCTCTAGGTCCAAGGATTCTCCCAACAAAATTACGCTTTAGAATGCTTAAAGATGGCAGAGTTGGAGG |
| A | RAD51AP1 | GCTAATCTGGACAAGCCAAGGCGGAGACTCTGATTGGGAGACTTCACAGACGATTAAGCCGTAGTTGAATTTATGGAGCGGTGCC |
| A | RASGRP1 | CAGGTTTCCATCTGCATCAAAAGATTGAATGCAGCTGTCAATGAGATCGTCTGTAACGGTAGATGGGAAAAAGTGTGCTCATTTT |
| A | RRAS2 | AGACCAGACCAGCAAGTAAAAGTTCTACCACAGTTTCTGTAGTTTCCACTCGCCGCGTTTCGTTGCGATAACCTCATATAGTCCC |
| A | SLC16A1 | TGTTACAGAAAGAAGCTGCAATCAAGCCACAGCCTGACAAGCAGCCACCACCCCTCTTAACTATAGCTCCATTTGGACAGACGTT |
| A | SNAP25 | GACACTCAAAGGACCGTGGCAGTAACTCTGTGGAATGTCACAGTTTTACACAAAGCAAACCTAATGAAGCCAGACACGAGATCAC |
| A | SOX4 | GCCGCCGCCAGCTCCCCAACGTGCAAATCCATTTCAGTTTGACCGTGAACCATTACGACTTTACGAGTTCGCAGAACAAAGACTT |
| A | ST3GAL5 | TGAGGTCATATCCAAAACCCGCCAAACTGACTTCATCGCACAGATGTGTGCAGCGAATCTGCAACTAACGCAAGTTACATCCTAG |
| A | SYT1 | GTCACAGTGTTACACAGAGCAGTCCTTTAAGACTCTTATGGAATGACTAGCGTGGTATCCCTGGTTGAGACATTGGACTAGTGTA |
| A | SYTL2 | ACCAGACCCTCTTAACTGTGGCACAGAATTGTGGTGTGAAACACATGATGCCAGCTGTCTCAATTATTGCTCAAAAACCACTATT |
| A | TCF19 | ATCTTCTGGCCAATCCCTCACTCATAACTGGTTGAACCTCCGAGGGCAATCTATCGTGGACCCAAAGTCGATACGTCCGATTAAG |
| A | TGFB1I1 | CCTTCTGCTCTCCTGAAGCCACCTTGCTAGATGGGAACTGAGACATGATTCTATGTCGTGCTATAATGGCGTCTGTCGTGCTCAT |
| A | TMEM156 | TAGTGTTATATTCCTCCAAGTGGTCAACCAGAGGAGCTACACTGAAGTTACAGTGCGTTTTATAAATACAGGGATGCTGTTTTTC |
| A | TMEM194A | CATGGACAGAAACTTCATTTGGCGTGAGGTGGGAAGAGCCTTCCACAAAGCTATTTGTGGCAGAATATGTCGATGCAAAAAACCT |
| A | TMPO | TTCACTCCGTATTTCACAAGCTGATCCAAAAGATCTTCATTAGTGAGCTCCCTCGAACAGTGATACGCACACTGATAACTATGCG |
| A | TOX2 | TGGCGGCCTGAGTGTCTCTGAAGAAGAGTGCGTAGGCCGACACAGCTATGATAAATGGTTTTCATCAGCCGGATTTTGTT |
| A | TTC9 | TATTTACACTGAATCAGTCCACAGTTCAATCCCCTAGCCTATACGATGCTCCTTGGTGAAAAGTCTCTTGTTATTTTGTGCTCGC |
| A | ZBTB41 | CAGAATCACCAGCCTCAGGTTCTTCTTCTGCATCATTATGATCCTTTTCACTGATGTACAGGGTGAAGGAACTGTAACGGCAGCT |
| A | ZEB2 | GCAGAGCAGGTTAGAACTGATCTCTTTCGGCCACTCCAGGAAACACAAACCTCTAAGGTAAAGTGCTTCTCAATAACATCCGCTC |
| A | ZNF559 | AGTAAGATGTAAGCATCGACAGGTTTCCTACGTTCAGTTTGTTTCCTAGGCAATCAGAAACCCTACTTGCCTGCTGCATGTTGTG |
| A | ZNF569 | TGATCATGTAATGTGCAATGAGGTGTTAATTCCTTCAGATGGCCTTGCCACTAGCATCTCTGACGAAAACAGCAGACGGAAAAGT |
| A | C2orf42 | CTTCAACACTAGGCTGCTTGCGTGCACCGTAGCGGAATATGGTTCCGAGCTATTGTAAATACGCAGCAACAAGCGTCCAG |
| A | DEDD | GCCTCTAATAAAGAGCCATTGATGTAGTCACGCCAGAATGCATCGAGGTACGATACCCGTAGCGTGATGGTCTTATAGCTGCTCT |
| A | GIGYF2 | TTCCCAATGCTGGTGTGCAGGTTGGAATGCGTATTGTTACGAGCTCTGTTCTCAAGAGCTTGCGAACAAGACGGAAGAAGATATG |
| A | HDAC3 | AGCAGGTAGATGGTTCGAGAACCAAATGTGGTCTCCAGACTCTTTCCCAGCCCCAACAACCGCCGGATATACGTTGGGATATAAA |
| A | PRDM4 | ACAATGCCTGGTTCTAATCCAACATTTCCATTGGACTGATAGGGATCTAGCGTTCAATCGCTTAGTTTCGTGGCGGGATTTGAGG |
| A | SART3 | AGGGCGCGCTCAAAGATCAACTGAATGCGAGCAGGATCGCCAATTTTCATCACTGGTTGGTTATCCAATGATCGACCTGGAGTCT |
| A | USP4 | AGGAAATAGGTTATGTTCACCCACATTGTACATGTCCCAGCTGTCAAAGCCTTTTAACAAACACTGGCTTTTTCCATCTTGTTGC |
| A | BIRC6 | TTCGGCTTTTCCTGAGTGGTAGATGTTACAACTGGAGCACTCACCTGTTGCGGGAAAACCATTGATCATGTATTCTTTGTCGCAT |
| A | EMC8 | ACATGCCACTTCTTAAAACGGTCAGCTTTCCACACAGGCTACAAGATATTCTCAGCATCTTTAGCAGTAGCACTTGCTAAATTGG |
| A | HADHA | CTTTTCAAAACCTTGGTAATCAAGCTGCCCAGTCAAGTTGCTGAAGATGGCATTTGGTGTTGCCTAGTACTAGGTGACTGGTACC |
| A | MAEA | GGAAGTAAGGCGGCACACAGAGGGTGTGATATCGAAACGACGCAGCTACGCATCAACACCAAACGCAATATCAGGCTGAATGGTG |
| A | MRPL18 | GCTGAGTCCTTATAACTCGCAACCTGTGCCAGAACTCACGGGAGGGAAACCCTAGTTGACTTGGAGTATGCCATGAAGACTCGTC |
| A | ORMDL1 | CGGCTGTTCATGACACGGGTATTTGGATTCACTTCACTGTGGGCAACTCCCGTTGGGAATGCCATCTCTATCCCTTCTTTTTCAA |
| A | PSMD11 | GATGGAATTCAAGAAGGGTCGTACATACTTCAGGAGTCCTCCAAGCTCTGCGCGATACTGATGATGAAATTTTCAAAAATGGCTG |
| A | RBM4 | CGGCGCGGACGCTTCTGACAAAACGCTAAAATGGCGGCGGCCGCAGCAGTCACTCACTAATTGTCCGTGCGACGACAAATTACCG |
| A | STX6 | TGGAGGCCCTTCAAATTAACACTGGTTCTGTAGGGACTGCCCAGTCAGCCCTATGTTGTTGTTGAGGAGATTTATGTTGTGGG |
| A | TRIM39 | ACTGCAGATTCTTCTCTCAGTCATTTCTGTGAAGATTACCCGAATAGCAGCTCTAATACATCGTGATACGGGCGATATAATGCTC |
| A | UBE2K | GTTGCAGTCTCTACATCCCATGATTTTGAAGACAAGGCCACTATTACTGCCGAATTTGTCCGCTGGGTAATGGTGGAATTAAAGA |
| A | NA | NA |
| B | ABAT | CGAAAGCCATGACCTCCGATCACTCCTCACAAGTGGTGTGAAGGTGGATGAGCAAAAGAGAGGAAGAGCCTCTTA |
| B | ABCC6 | CGAAAGCCATGACCTCCGATCACTCTGAGAACTGTGAGAGTCACAAAGGCTTTCTCTGCATTCATAGCATTCTCG |
| B | ACOX2 | CGAAAGCCATGACCTCCGATCACTCCATGGCAGCCTTATAACGCTCATTCTGGGTCATGAAATAATTGTCCTTAC |
| B | AGPAT9 | CGAAAGCCATGACCTCCGATCACTCATCTTCATGTAGATCTCGGAGATGCCCAGAGACACTCCGAAGACCGAAGG |
| B | ANXA1 | CGAAAGCCATGACCTCCGATCACTCGCAAAGCGTTCCGAAAATCTCCAGATGTGTCTGAGGTTATGTCTTTGGCC |
| B | ARL14 | CGAAAGCCATGACCTCCGATCACTCTTCGCAGCCTCAGTTCTGCTTGAAGAACGCCAAAGTGTCTCCTCTTGATT |
| B | BDH2 | CGAAAGCCATGACCTCCGATCACTCTGCCTTGATCATCAGGTACATGCTGCGCACATTGAGATTCATCGAGAAGT |
| B | BIRC5 | CGAAAGCCATGACCTCCGATCACTCAGCAGTGGCAAAAGGAGTATCTGCCAGACGCTTCCTATCACTCTATTCTG |
| B | BLM | CGAAAGCCATGACCTCCGATCACTCATGACAACAGTGACCCCAGGAGAAACACAGGCAGGGAGCTGGTAACACAA |
| B | BUB1B | CGAAAGCCATGACCTCCGATCACTCCCTTGAGAGCACCTCCTACACGGATGATTGGAGCTCTTGCTGTCTTTTTC |
| B | CCNE2 | CGAAAGCCATGACCTCCGATCACTCGGCTGCTGCTTAGCTTGTAAACGGCTACTTCGTCTTGACATTCTCTTCTT |
| B | CDC6 | CGAAAGCCATGACCTCCGATCACTCGCTCTCCTGCAAACATCCAGTGCTTTGCGAACATCTCCTGAAACAGCAGA |
| B | CDCA4 | CGAAAGCCATGACCTCCGATCACTCCCAGATAATACAGCAGAACTGATCCTGCAGAAAGGTTGCTGGAGGGTCAG |
| B | CDK6 | CGAAAGCCATGACCTCCGATCACTCCCGCACGCGCTTCAACGCCACGAAACGGCCTCCGTTCT |
| B | CHST11 | CGAAAGCCATGACCTCCGATCACTCTCTGCTGTTGCTCATGAATAGTCCAAACCTCATGTCAAGTCCATGAATAA |
| B | CKS1B | CGAAAGCCATGACCTCCGATCACTCGTTTCTTGGGTAGTGGGCGCCGGAACAGCAAGATGTGAGGTTCTG |
| B | CPM | CGAAAGCCATGACCTCCGATCACTCAACTCTGGAATCCCAATTCTGTGTTCCTTTGGAAACCGCCCCACAACAAG |
| B | CYBRD1 | CGAAAGCCATGACCTCCGATCACTCCTGTGTAGAGTCATGATCTAAACCCTGTCCCAGATATTCCTTATCGGATT |
| B | CYP1B1 | CGAAAGCCATGACCTCCGATCACTCCACCGCCTTTTGCCCACTGAAAAAATCATCACTCTGCTGGTCAGGTC |
| B | DOCK4 | CGAAAGCCATGACCTCCGATCACTCGTGATGAAGACATCCTGAGCCACCTTCTAGCGTGAAACCTCAACCAAACA |
| B | DTL | CGAAAGCCATGACCTCCGATCACTCGACCATAATGTTGCCATCTCTTCCACCCGTACAGAATACAGCTTTCTCAA |
| B | EVI5 | CGAAAGCCATGACCTCCGATCACTCTAAAAACTGGCAGCTGACTGCAATAGCTTGTAGGCTGCACTTTGCTGACC |
| B | FIGNL1 | CGAAAGCCATGACCTCCGATCACTCGGGCATTCTGAAGGAGTTTCAAACGATTGCTCTCCGGGAAGTCTTGGGTC |
| B | FN1 | CGAAAGCCATGACCTCCGATCACTCTCTTCATGACGCTTGTGGAATGTGTCGTTCACATTGTAAGTGATGTCATC |
| B | GBP2 | CGAAAGCCATGACCTCCGATCACTCCAAGTAGTCATCAGCAGTGATGGGTTCTCCATCTACTTCCA |
| B | GINS1 | CGAAAGCCATGACCTCCGATCACTCCATTTGGCAAGACGCTACCATATTCCCATCTGAGTGCTCTGATCCGAAGC |
| B | GLIPR1 | CGAAAGCCATGACCTCCGATCACTCCAATTTGGGCTAGTGCTGGGTCCCAAGTCATGTATAGCATATCAC |
| B | GNG2 | CGAAAGCCATGACCTCCGATCACTCCTAGAATGTCCATGATTCAGGGAAAGAGGGAATTTGCTCATACTCATGGC |
| B | HAUS6 | CGAAAGCCATGACCTCCGATCACTCCATTGCCAACGCCTGCTCCTTTCACGCCCAGAGCGATTTCGCCTCAAAGG |
| B | IDS | CGAAAGCCATGACCTCCGATCACTCATGATCCGAGGTAAATGCAATGATGGTGCTGTTGGCCAGCTGAAGATCGT |
| B | IMPA2 | CGAAAGCCATGACCTCCGATCACTCTGTACAGCCGCTCCTCTGTGCAGTGGTAAATCACTCCGAATTCAAG |
| B | IRS1 | CGAAAGCCATGACCTCCGATCACTCCGTTGCTCAAAGGAAACCCTTCGTGTCGCCAGAGTATGAACTTTTGGTTT |
| B | ISG20 | CGAAAGCCATGACCTCCGATCACTCTAATCGGTGATCTCTCCCTCAGGCCGGATGAACTTGTCGT |
| B | ITGB8 | CGAAAGCCATGACCTCCGATCACTCATGCTGTTGTTCCATGAGAGCACATGAGGTTTTGCACTGATCAAGTATAG |
| B | KIF4A | CGAAAGCCATGACCTCCGATCACTCCCCTCATCATCATCACCATCACCATCCTCATGCTCATTCA |
| B | LAMC2 | CGAAAGCCATGACCTCCGATCACTCCCACCTGAAAGGACTGATCACTGACTCCCTGAAGCCGAGACACTGAATCC |
| B | LAT2 | CGAAAGCCATGACCTCCGATCACTCGAGATACTTTTTCTGGCAACATTTCTGACTCAAGGTCCCTCTGGG |
| B | LMNB1 | CGAAAGCCATGACCTCCGATCACTCTTGCGCCAGCTTGTACTCATACTCAATTTGACGCCCAGAATCCACCTCTA |
| B | MAP2 | CGAAAGCCATGACCTCCGATCACTCGCCTTTCTATGGTAACAGGCTCTAGAATTATCAGAAGAAAGAAACCCCCC |
| B | MCM10 | CGAAAGCCATGACCTCCGATCACTCCATCATCAGCCTCTTCTGTATAAGATTCACCGTCGCCGTCGGCATCAAAG |
| B | MCM2 | CGAAAGCCATGACCTCCGATCACTCAGGTACTGAGTGGAGACAAAGCACTCGGAGGTGGCAAGATGTTCAGCAAC |
| B | MDFIC | CGAAAGCCATGACCTCCGATCACTCCTTCCACTCCTACAATCCTGAAACACTTCTCCCTCTGTTGCTCTTTT |
| B | MEX3D | CGAAAGCCATGACCTCCGATCACTCATTAACAGAAAGTTAGAAATTTGATTGGAACGCCCCTCGCCCCCGCCCCC |
| B | MYB | CGAAAGCCATGACCTCCGATCACTCCAAGGGGCTCGCCAGGGACCTGTTTTTAGGTACTGTAAATGCTTTGAGAA |
| B | NCAPD2 | CGAAAGCCATGACCTCCGATCACTCGGACAATGGGAAGTGGAGACTTTTCCAGCATGGTGAACAGAAGACGAAGC |
| B | NCAPG | CGAAAGCCATGACCTCCGATCACTCTCTGGCGAGCACGGACTTGTTAAGATCTCATTGCAAATTTTCATAGCCAA |
| B | NDC80 | CGAAAGCCATGACCTCCGATCACTCCTTCATGCTTATGACCTTTTTTCCAGTTTCCTGACACGACCC |
| B | NRP1 | CGAAAGCCATGACCTCCGATCACTCAATGCAGCAAAGAGGAGAATCTAAGCGATCCGAAGAGCCCCAACTCCGCC |
| B | PLAUR | CGAAAGCCATGACCTCCGATCACTCCAGAGTTGCCCTGGTTGCACAAGTCTAACCCACACACAACCTCGGTAAGG |
| B | PLK1 | CGAAAGCCATGACCTCCGATCACTCATCCCCTATTTTCACCTCCAGATCTTCATTCAGGAAAAGGTTGCCCAGCT |
| B | PLSCR4 | CGAAAGCCATGACCTCCGATCACTCACAAGTTTGTCTTCAGTATGAGGACATGTTGACGGAGAGGAAAGGTAGGA |
| B | PRMT3 | CGAAAGCCATGACCTCCGATCACTCCTCCAAGAACCTTCTTCGCCCCAGCTTTAGCAGCAAACATAGAGAGAATT |
| B | PTP4A1 | CGAAAGCCATGACCTCCGATCACTCTCCAGGAGTGCACACATGTCACATGAGGAGACTCGAAGTTTGCCTGA |
| B | QKI | CGAAAGCCATGACCTCCGATCACTCCCTTTGCCTCGGACCATGATTTTACATCCGGTTTCTGCTTCAA |
| B | RAD51AP1 | CGAAAGCCATGACCTCCGATCACTCTGTACCACACTCAGGTGCTAGTGGCATTTGGATGCAAAGGTTTAACTCGT |
| B | RASGRP1 | CGAAAGCCATGACCTCCGATCACTCACAATTCGGTGCATGGTCAGCATGACTTGCAACAGTTGGTTACTTCGACA |
| B | RRAS2 | CGAAAGCCATGACCTCCGATCACTCGGGAAGGTTTCTTGAGTAGTTATGTGACTGGCCAAAGATGGGTACAACCA |
| B | SLC16A1 | CGAAAGCCATGACCTCCGATCACTCAAGCCCAAGACCTCCAATGACTCCAATACAGACGTATAGTTGCTGTACGG |
| B | SNAP25 | CGAAAGCCATGACCTCCGATCACTCTAACAGCCATGAGGAACCAAAGACGGCACATTTTGAGAGATTCAGAGCCT |
| B | SOX4 | CGAAAGCCATGACCTCCGATCACTCCTCACCTCACTGATTTCACGTAGAAAAGAAGGCGGAGGCCCAGCAGCC |
| B | ST3GAL5 | CGAAAGCCATGACCTCCGATCACTCAGCAGCCATGCATTGACTGTCGAAGTAGTGCAAAGGTGTTCTGGGTTGAT |
| B | SYT1 | CGAAAGCCATGACCTCCGATCACTCAAATTGAGTGCTATCCACTGAGGAAACTACGGGTCTAAGCACACACGGCA |
| B | SYTL2 | CGAAAGCCATGACCTCCGATCACTCTATCTTTCTGCGACTTTTTCCCACAACCTACAAAGGTCCTGCAAGGGT |
| B | TCF19 | CGAAAGCCATGACCTCCGATCACTCAAACTTGCTTATCCTAATAGAGCACTCCTGCTTGCCTGCGCCCCG |
| B | TGFB1I1 | CGAAAGCCATGACCTCCGATCACTCGCTGGAAGGGAGGCTGGGTCTTTTCTTATCTTCAGACTGGTCCT |
| B | TMEM156 | CGAAAGCCATGACCTCCGATCACTCCATCCTCCATGATTGTTGATCTTCCAGTGTGGTTTTTTAGATGACAGG |
| B | TMEM194A | CGAAAGCCATGACCTCCGATCACTCCTCATAGATTTCATCCTGGGCAATAATGCTCCCTAATCCATACTCCTGCT |
| B | TMPO | CGAAAGCCATGACCTCCGATCACTCAAAGCTTTTTCTCATATAGCTTCCTGGTTGTTCCCACAATAGGACCAGGA |
| B | TOX2 | CGAAAGCCATGACCTCCGATCACTCCACGATTTTGGACACGTCACCGAAAGTGGCACTGGGGTTCTGACCCTTGA |
| B | TTC9 | CGAAAGCCATGACCTCCGATCACTCGGGCTGCTTCCAACAGAACTGACAGGAACTACTACCTGTTAATTTGTTTT |
| B | ZBTB41 | CGAAAGCCATGACCTCCGATCACTCGTTGCTGTTCTGAATGACCACTGGAGTTAACCCCTCATGAACATTTCCTA |
| B | ZEB2 | CGAAAGCCATGACCTCCGATCACTCACTGTACAAAAACCTCGCCAAGAGTGTCGGGAGGCAGGACCGTTATTCCT |
| B | ZNF559 | CGAAAGCCATGACCTCCGATCACTCCTTCCTCACATTCATAGCATTTCTCTCCAGCATGAGTTCAAACAGACTC |
| B | ZNF569 | CGAAAGCCATGACCTCCGATCACTCGTGGCTTTTTCAGAAGGCTATCCCACATTCATAGTTTTCTACTCTGGAAG |
| B | C2orf42 | CGAAAGCCATGACCTCCGATCACTCTTGCCGCACTGAGTAGACCTGAAGATCAGAGCCTGTAATGATTTTGACAG |
| B | DEDD | CGAAAGCCATGACCTCCGATCACTCCCCACAGCTTGCTTGAGGGAGTCTGTGATGAAGACACCTTTAAGT |
| B | GIGYF2 | CGAAAGCCATGACCTCCGATCACTCCAGATGCCCACTGGTTAGGAGGACCAGTATTTATAGAGCCCCAAACAGAA |
| B | HDAC3 | CGAAAGCCATGACCTCCGATCACTCATCTCCATCCCTAATAGGTACCATTGTCAGGCCTTGGGAGAGAGAGGAAA |
| B | PRDM4 | CGAAAGCCATGACCTCCGATCACTCGATGAAGACTTTGGGCACCATGTGTGTTCACAGAGCGAGAGTCTATTGAA |
| B | SART3 | CGAAAGCCATGACCTCCGATCACTCGGTACTGACTGTAACGGATCCATAAGTCTGGGACAAGGCAGTTCTCGACC |
| B | USP4 | CGAAAGCCATGACCTCCGATCACTCAAGGTCTGACTCTCAGGATCTGAAAATAGCCCAGAGTTGTCTATTGGGCC |
| B | BIRC6 | CGAAAGCCATGACCTCCGATCACTCCTAACTCCCCTGACTGTTCAATGGTCACCCATTCAAACTGATCGCTATCC |
| B | EMC8 | CGAAAGCCATGACCTCCGATCACTCACTCTGTGTTAAAAACACGACCGACTGAACATTCTGCCCCCTTTCAAGGC |
| B | HADHA | CGAAAGCCATGACCTCCGATCACTCCTGTGCTTAAGACTAAGGTCCTCAAACACAGCTTCAATCACCATGTCGGC |
| B | MAEA | CGAAAGCCATGACCTCCGATCACTCTCTGCAGAAATTTGGTCCCGTGGATTTCCACGTTATACATTCTCGAAGCA |
| B | MRPL18 | CGAAAGCCATGACCTCCGATCACTCCGAAACCACAACCTTGCCATTCTGATGCTCCACAAGTGCTTCTACATGAT |
| B | ORMDL1 | CGAAAGCCATGACCTCCGATCACTCAGACAATATGAAGCAAGCCAACTCCCAATGCATATGTCAGCCACATACCC |
| B | PSMD11 | CGAAAGCCATGACCTCCGATCACTCAGAAACAGATCAAGAAGAGATCGGACCAGGCGAGCTGCTTTAGCCTTGCT |
| B | RBM4 | CGAAAGCCATGACCTCCGATCACTCCTGACAAGAGCCCGCACAGAAACCAGCAGGGCCTCCTCCTCG |
| B | STX6 | CGAAAGCCATGACCTCCGATCACTCATGTAGGTTCCCTGGATTGTTCTCTTTGGCTGCCACATTTGGAGCCTGAG |
| B | TRIM39 | CGAAAGCCATGACCTCCGATCACTCTGGACAAAGTCCTGTTGAGGTGGTAGGAGGAACTGAAATGCCCTCAGTAA |
| B | UBE2K | CGAAAGCCATGACCTCCGATCACTCCTTGACTATATCAGCAGCTCTCTATGCCTCAGTTACTCAGAAGCAATTCT |

Table S5 Eighteen genes used as internal controls

| Gene | Mean.Expression | Standard.Deviation | SD.mean |
| --- | --- | --- | --- |
| HADHA | 3·11 | 0·34 | 0·11 |
| MRPL18 | 2·50 | 0·43 | 0·17 |
| SART3 | 2·47 | 0·39 | 0·16 |
| COX4NB | 2·50 | 0·39 | 0·16 |
| UBE2K | 2·87 | 0·49 | 0·17 |
| GIGYF2 | 2·36 | 0·47 | 0·20 |
| PSMD11 | 2·74 | 0·45 | 0·16 |
| DEDD | 2·15 | 0·45 | 0·21 |
| C2orf42 | 1·97 | 0·33 | 0·17 |
| STX6 | 2·48 | 0·43 | 0·17 |
| MAEA | 2·40 | 0·46 | 0·19 |
| HDAC3 | 2·46 | 0·40 | 0·16 |
| PRDM4 | 2·16 | 0·38 | 0·18 |
| TRIM39 | 1·99 | 0·36 | 0·18 |
| USP4 | 2·23 | 0·43 | 0·19 |
| RBM4 | 2·58 | 0·27 | 0·10 |
| BIRC6 | 2·76 | 0·40 | 0·14 |
| ORMDL1 | 2·82 | 0·38 | 0·13 |
| Standard D | eviation (SD) |  |  |

## Statistical analysis

A series of data analyses were performed from microarray analysis in cell line data and in patient data, E2F signature optimization in NanoString platform, E2F scoring system development, assessment of median-cutoff E2F score, and validation of the prognostic and predictive E2F signature. Briefly, in cell lines, microarray data were normalized by the RMA method and differential expressed genes were selected by comparing siRNA-treated knockdown versus control A549 and H1299 cell lines with a number of filters. GeneGo MetaCore was used for pathway enrichment analysis. Microarray data processing in patient samples included IRON v2.1.5[^9^](#_ENREF_9) normalization method to adjust for minor differences in dynamic range between samples, and COMBAT[^10^](#_ENREF_10) method for correcting batch effect. Data process for NanoString platform was based on NanoStringNorm R package[^11^](#_ENREF_11) to normalize gene expression. For optimization of E2F signature in NanoString platform, Spearman and Pearson correlation analyses were first used to remove poor correlated genes in NanoString platform (between FF and FFPE). They were also used to analyze the E2F gene signature using the PC1 and the corresponding loading coefficients to see if the entire signature was well preserved from FF to FFPE in the matched paired samples, as well as reproducibility between FF and FFPE tissues and between microarray and NanoString platforms. To validate the E2F signature, we employed the training and validation scheme. Specifically, we used the MLOS cohort and the MLCom cohort as the training set for the microarray and NanoString platforms, respectively. The validation cohorts were MCLA, TCGA, and JBR10 for the microarray platform and LCBRN and NATCH cohorts for the NanoString platform. Principal component analysis was performed to derive a PC1 in the training cohort for the E2F scoring system development. Percentage of total variation contributed by PC1 was evaluated. Robustness of the PC1 loading coefficients was evaluated by a bootstrapping approach. The PC1 was then used to calculate E2F score in the training and validation cohorts. Performance of median threshold of E2F score was done by comparing with other cutoffs in terms of prognostic and predictive effects using the log-rank test and the Cox proportional hazards model, respectively. To validate the prognostic and predictive effects of the E2F signature, each platform used the corresponding training cohort to classify patients into low or high E2 score groups for the training and validation cohorts. The classified groups (low and high E2F) in each cohort or combined cohort were then used for the following analyses. Particularly, validation of the prognostic E2F signature was analyzed to see any difference of survival curves between low and high E2F score groups using the log-rank test or by Cox proportional hazards model for covariate adjustment. For validation of the predictive E2F signature, the Cox proportional hazards model was used to examine any differential treatment effect by testing interaction effect while the log-rank test was used to test the treatment effect (ACT versus non-ACT) in each risk group (Low or high E2F). Proportional hazards assumption was performed for the Cox model analyses. All statistical analyses were performed using R version 3.2.2 and were described in detail in the Supplementary R Markdown file. Below is the detailed description of data analysis for each component.

### Microarray analysis in siRNA-treated knockdown and control cell lines

Each sample was then subjected to Affymetrix-based microarray analysis, and a number of filters applied (Figure S1B) to generate an initial list of E2F-regulated genes. Samples from siRNA-treated knockdown and control A549 and H1299 cell lines were normalized separately with the RMA method using Affymetrix Power Tools software, v1.12.0 (Affymetrix, Inc., Santa Clara, CA, USA), due to large differences in gene expression between cell lines. Log2 ratios were then calculated between knockdown and control. The following filters were then applied to identify differentially expressed probesets. 1) For each knockdown, low expressing probesets were discarded by requiring at least one sample to express a normalized log2 intensity greater than 6. 2) Second, we required both cell lines to agree in direction of change, to change by more than $\pm$ 1.1-fold, and at least one of the cell lines must change by at least $\pm$ 1.5-fold. 3) The final list of E2F-related genes was assembled by including all probesets that were differentially expressed in at least 5 of the 6 knockdown conditions. 4) As a final filter to reduce the number of genes in the signature, probesets that differ between tumor and adjacent normal lung tissue were identified using GEO datasets GSE18842 (45 adjacent normal tissues and 46 tumors) and GSE19188^10^ (58 adjacent normal tissues and 87 tumors, after discarding outlier samples). Each dataset was normalized with IRON[^9^](#_ENREF_9) and analyzed separately. For each probeset, the average and standard deviation (SD) of the adjacent normal log2 intensities were calculated. Upper and lower bounds for baseline adjacent normal expression were set at $\pm$ 3 SD from average. The number of samples outside $\pm$ 3 SD was counted for both adjacent normals and tumors. A probeset was identified as differentially expressed within a subset of tumors if the following criteria were met: (A) must have at least three log2 intensities $\geq$ 5 across all samples, (B) must have at least three tumor samples outside 3 SD (significant), (C) the frequency of significant samples within tumors must be at least twice that observed within adjacent normals, and (D) significant tumor samples must be at least 1.5-fold further from the adjacent normal average than significant adjacent normals. The lists of differentially expressed probesets from each dataset were then intersected. The intersection of this list with the E2F-related signature results in 145 probesets. Entrez GeneIDs for the intersected probesets were entered into GeneGo MetaCore for pathway enrichment analysis.

### Data processing of microarray platform in patient data.

Microarray analysis of patient data included IRON v2.1.5[^9^](#_ENREF_9) normalization method to adjust for minor differences in dynamic range between samples, and COMBAT[^10^](#_ENREF_10) method for correcting batch effect.

### Data processing of NanoString platform.

Gene expression was normalized using NanoStringNorm R package[^11^](#_ENREF_11). Specifically, the geometric mean was used to summarize positive controls and housekeeping genes and mean ± 2 standard deviation was used for background correction.

### E2F signature optimization in NanoString platform.

We incorporated 106 well-annotated genes (13 poorly annotated genes were excluded) from the microarray-based 119-gene signature and 9 internal controls into a NanoString^TM^ Legacy assay. NanoString analysis was then performed on the MLTO cohort (36 Moffitt LUAD patients for technical optimization of the NanoString assay) using the matching RNA from FF tissue and from FFPE blocks. The paired data were used to determine the best codesets to be represented in the final NanoString assay by Spearman and Pearson correlation analysis (individual gene level). Pearson correlation of 0.5 was used as the threshold to filter out poor correlated genes. Since Pearson correlation analysis is sensitive to outlier and normality assumption, we also used Spearman correlation analysis to examine if these correlations by Pearson method were similar to the ones by Spearman method. Both correlation analyses were also used to evaluate the gene signature using the PC1 and the corresponding loading coefficients to see if the entire signature was well preserved from FF to FFPE in the matched paired samples. Since the "MLTO" cohort had microarray data from FF tissues, the same correlation analyses and PCA were performed between microarray and NanoString platforms.

### E2F scoring system

The overall E2F score was generated by principal component analysis (PCA) to reflect the combined effect of the E2F gene signature. We used the first principal component (PC1) as the E2F score to represent the overall expression level for the signature. That is, PC1, defined as $\sum w_{i}x_{i}$, is a weighted average expression among all genes in the E2F signature, where $x_{i}$ represents gene i expression level, $w_{i}$ is the corresponding weight (PC1's loading coefficient for gene i) with $\sum w_{i}^{2}=1$, and the $w_{i}$ values maximize the variance of $\sum w_{i}x_{i}$. Prior to PCA, data were standardized by centering the mean and scaled by the standard deviation for each gene in the training cohort. The standardized expression data were then used in PCA to generate the PC1's loading coefficients. These fixed PC1's loading coefficients derived from the training cohort were then used to calculate PC1 scores using the standardized expression data in training and validation cohorts. The uniqueness of the PC1 scoring system is that it explains the largest total variation, likely linked to biological effect. More importantly, it integrates all molecular features into one score (efficient data reduction) for each patient, simplifying clinical decision-making. This approach has been used to derive various gene signatures previously. For example, in our studies of lung and breast cancer, we used PC1 to capture most gene signature information and this PC1 scoring approach was able to demonstrate the clinical association (e.g., cancer risk, prognosis, and prediction of chemotherapy) of gene signatures. In our experiments, PCA was performed by singular value decomposition of the correlation matrix (data centered and scaled) with orthogonal rotation using the r function 'prcomp'. The MLOS cohort was the training set while MCLA, TCGA, JBR10, and LCBRN were the validation cohorts for the FF platform to develop and validate the E2F score (PC1). For the FFPE platform, we used the MLCom cohort as the training set and NATCH cohort as the validation set.

### Median-threshold of E2F score for risk classification.

There are various ways to determine a threshold of E2F score for risk classification. For example, a common approach is an optimum-based approach, using a cutoff at the smallest p value. While it is appealing, it often leads to a local maximum (e.g., strong association in the training set, but weak association in validation sets). In contrast, our experiences in the median cutoff had enjoyed success in many gene signature developments. Thus, our evaluation focused on robustness of the median-cutoff E2F score compared to other percentile cutoffs (e.g., 30th or 60th percentile) in training cohort in terms of prognostic effect. Post-hoc assessment of the E2F median cutoff classification was also performed from the training cohorts to validation cohorts for the prognostic and predictive effects. A reliable median-cutoff E2F score should yield significant results in most training and validation cohorts. In the evaluation process, a cutoff of the E2F score from the training cohort was used to assign patients into high and low risk survival groups. The unit cutoffs ranged from the 25th to 75th percentile. At each cutoff, we compared the low score group (below the cutoff) and the high score group (above the cutoff) for the survival differences in non-ACT patients of the training cohort using the log-rank test to assess the prognostic effect. For the post-hoc evaluation, we extend assessment of the prognostic effect to the validation cohorts, in addition to the training cohort. Moreover, we compared survival difference of ACT-treated patients with untreated patients in the high and low E2F score groups, separately, using the log-rank test, and the combined group using the Cox proportional hazards model with an interaction term, to evaluate the predictive effect in the validation cohorts. In our experiments, the training set was the MLOS cohort in the FF platform and the MLCom cohort in the FFPE platform. Validation cohorts for the post-hoc evaluation included MCLA, TCGA, JBR10, and LCBRN for the FF platform and NTACH cohort for the FFPE platform.

### Validation of the prognostic E2F signature.

The E2F prognostic effect was evaluated by various ways of analyses in non-ACT patients from FF to FFPE platforms, from all stages to each stage, from OS to PFS, from individual cohort to combined cohorts. The purpose was to test whether patients identified with a low E2F score had a better survival than the patients with a high E2F score. Three sets of data were analyzed: (1) FF-based cohorts (MLOS, MCLA, TCGA, JBR10, and LCBRN), (2) FFPE-based cohorts (MLCom, and NATCH), and (3) two randomized clinical trials (JBR10: FF-based microarray and NATCH: FFPE-based NanoString). To follow the training-validation procedure, we used the MLOS cohort as the training cohort for the FF platform. The training cohort for the FFPE platform was the MLCom cohort. In each platform, the training cohort was used to classify patients into low or high E2 score groups for the training and validation cohorts. The classified groups (low and high E2F) in each cohort or combined cohort were then analyzed to see any survival difference by the log-rank test or by Cox proportional hazards model for covariate adjustment. Proportional hazards assumption was performed for the Cox model.

### Validation of the predictive E2F signature

Gene expression data from two randomized clinical trials were used for validation: JBR10 (JBR10.NCSLC and JBR10.AD) and NATCH cohorts. The key analysis was to test whether patients with ACT had a better survival than patients without ACT in the high E2F score group. And the low E2F score group had a reverse trend (a better survival in patients without ACT). Most importantly, we would like to know if there was a differential treatment effect, meaning that ACT effect was different between low and high E2F groups (interaction effect in statistical term)? Analysis also covered from all stages to each stage, from OS to PFS, from individual cohort to combined cohorts. Same as the previous procedure, the training cohort was the MLOS cohort for the FF platform and the MLCom cohort for the FFPE platform. The E2F classified groups in each cohort or combined cohort were evaluated to examine any differential treatment effect by testing interaction effect in the Cox proportional hazards model and to test the treatment effect (ACT versus non-ACT) in each risk group (Low or high E2F) by the log-rank test. Covariate adjustment and proportional hazards assumption were also examined for the Cox model.

# Results

## A siRNA-derived E2F signature is representative of cell cycle (Table S6 and Figure S1)

We utilized an RNAi-based approach to avoid the problem with previously derived E2F signatures that utilized overexpression[^12^](#_ENREF_12). We independently depleted A549 and H1299 cells of E2F1, E2F3A, E2F3B, both 3A and 3B, E2F4, and Rb. Independent depletion was confirmed by Western blotting (see Figure S1). Each sample was then subjected to Affymetrix-based microarray analysis, and a number of filters applied to generate an initial signature of 471 probesets. Considered too large to represent a practical clinical signature, this set of 471 was filtered through two published datasets (GSE18842 and GSE19188) to identify those genes that were the most differentially expressed in LUAD tumors compared to normal adjacent lung tissues. This 145-probeset microarray signature (corresponding to 119 coding genes) was subjected to GeneGo analysis, where it was observed that the cell cycle was the most significantly correlated pathway (see Table S6).

Figure S1: Targeted siRNAs effectively and specifically inhibit expression of the expected E2F/Rb family members.


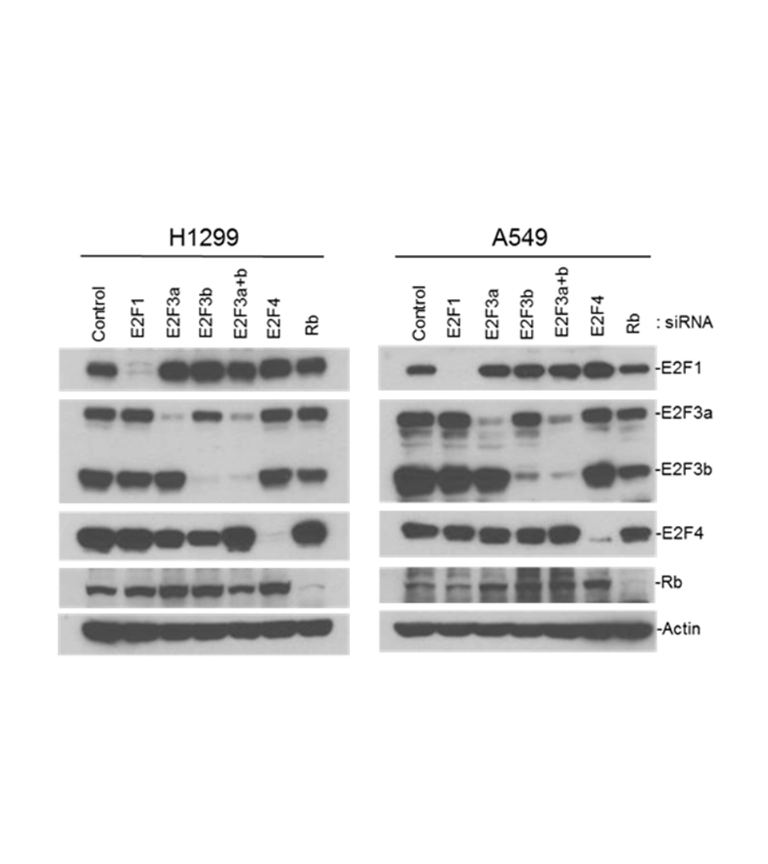


Table S6: GeneGo analysis. The 119-gene (145-probset) E2F signature was analyzed via GeneGo and ordered in the table below based on p-values. Cell cycle was the most highly represented pathway (orange highlighting), as expected^17,18^. As anticipated from our previous work, DNA damage/apoptosis^19-25^ and cell adhesion/signaling^26-28^ were also well represented (yellow highlighting).

| Pathway.Mappings | p.value |
| --- | --- |
| Cell cycle_The metaphase checkpoint | <0.001 |
| Cell cycle_Chromosome condensation in prometaphase | <0.001 |
| Cell cycle_Role of APC in cell cycle regulation | <0.001 |
| Cell cycle_Start of DNA replication in early S phase | <0.001 |
| Apoptosis and survival_DNA-damage-induced apoptosis | 0.003 |
| Cell adhesion_ECM remodeling | 0.003 |
| Cell cycle_Initiation of mitosis | 0.009 |
| G-protein signaling_TC21 regulation pathway | 0.009 |
| DNA damage_ATM/ATR regulation of G2/M checkpoint | 0.01 |
| Neurophysiological process_Role of CDK5 in presynaptic signaling | 0.011 |
| Cell cycle_Role of SCF complex in cell cycle regulation | 0.012 |
| DNA damage_Role of Brca1 and Brca2 in DNA repair | 0.013 |
| DNA damage_ATM/ATR regulation of G1/S checkpoint | 0.015 |
| Cell cycle_Role of Nek in cell cycle regulation | 0.015 |
| Cell cycle_ESR1 regulation of G1/S transition | 0.016 |
| Apoptosis and survival_Caspase cascade | 0.016 |
| Development_TGF-beta-depdendent induction of EMT via SMADs | 0.017 |
| Cell adhesion_Plasmin signaling | 0.017 |
| Influence of low doses of Arsenite on Glucose stimulated insulin secretion in pancreatic cells | 0.018 |
| Cell adhesion_Chemokines and adhesion | 0.02 |
| Cytoskeleton remodeling_Cytoskeleton remodeling | 0.021 |
| Cell adhesion_PLAU signaling | 0.021 |
| Development_VEGF-family signaling | 0.023 |
| Nicotine signaling in dopaminergic neurons. Pt. 2-axon terminal | 0.026 |
| Cytoskeleton remodeling_TGF, WNT and cytoskeletal remodeling | 0.026 |

## Translation of the E2F signature to a NanoString format

There were 106 genes (from the 119 genes) were found well-annotated. Thus, these 106 genes were incorporated into the NanoString assay.

Three aspects were evaluated in the "MLTO" cohort.

### a. Side-by-side comparison of individual gene expression between FF and FFPE to eliminate poor correlated genes (Table S7 and Figure S2).

Pearson correlation of 0.5 was used as the threshold to filter out poor correlated genes. As a result, there were 32 genes with a correlation less than 0.5 by Pearson correlation analysis (Figure S2A). Since Pearson correlation analysis is sensitive to outlier and normality assumption, we also used Spearman correlation analysis to evaluate if these correlations by Pearson method were similar to the ones by Spearman method. Result showed that the Pearson correlations of these 32 genes were not deviated away from the Spearman correlations (24 genes with a Spearman correlation less than 0.5 and 8 genes with a Spearman correlation 0.5-0.6; Figure S2B). Therefore it led to a 74-gene signature.

Figure S2
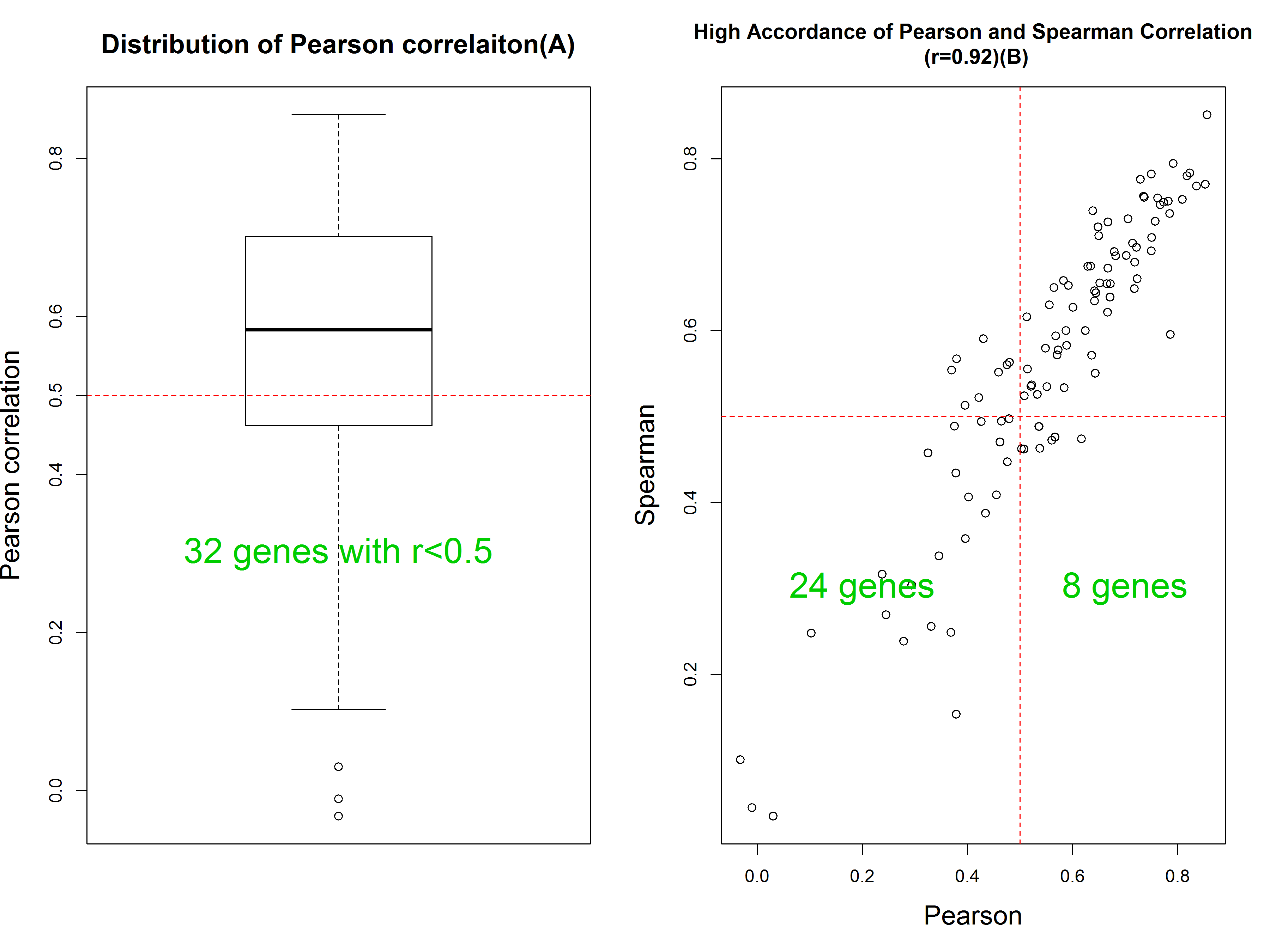


Table S7: Pearson and Spearman correlation coefficients of the 106 genes

| Gene | Pearson_r | Pearson_pvalue | Spearman_r | Spearman_pvalue |
| --- | --- | --- | --- | --- |
| ABAT | 0.5325235 | 0.0008278 | 0.5258687 | 0.0011615 |
| ABCC6 | 0.7498996 | 0.0000001 | 0.7824968 | 0.0000003 |
| ACOX2 | 0.7227366 | 0.0000006 | 0.6602317 | 0.0000192 |
| AGPAT9 | 0.5374440 | 0.0007246 | 0.4630631 | 0.0048534 |
| AK2 | 0.4258439 | 0.0096120 | 0.4942085 | 0.0024637 |
| ANKDD1A | 0.4341846 | 0.0081490 | 0.3876448 | 0.0201346 |
| ANXA1 | 0.7215232 | 0.0000007 | 0.6967825 | 0.0000047 |
| ARHGDIB | 0.3248948 | 0.0531886 | 0.4576577 | 0.0054280 |
| ARL14 | 0.7857283 | 0.0000000 | 0.5956242 | 0.0001701 |
| BDH2 | 0.7359241 | 0.0000003 | 0.7552124 | 0.0000006 |
| BIRC5 | 0.5723720 | 0.0002647 | 0.5776062 | 0.0002905 |
| BLM | 0.5220962 | 0.0010906 | 0.5369369 | 0.0008786 |
| BUB1B | 0.6713093 | 0.0000074 | 0.6391248 | 0.0000411 |
| CCNE2 | 0.5663906 | 0.0003171 | 0.4761905 | 0.0036727 |
| CDC6 | 0.5838334 | 0.0001855 | 0.5335907 | 0.0009569 |
| CDCA4 | 0.5640721 | 0.0003397 | 0.6501931 | 0.0000277 |
| CDK6 | 0.5677492 | 0.0003044 | 0.5940798 | 0.0001783 |
| CENPF | 0.4214745 | 0.0104638 | 0.5220077 | 0.0012777 |
| CENPQ | 0.4643951 | 0.0043274 | 0.4947233 | 0.0024350 |
| CHST11 | 0.6439601 | 0.0000226 | 0.6440154 | 0.0000346 |
| CKS1B | 0.6484833 | 0.0000189 | 0.7207207 | 0.0000019 |
| CPM | 0.6384432 | 0.0000279 | 0.7395109 | 0.0000010 |
| CYBRD1 | 0.6495722 | 0.0000181 | 0.7104247 | 0.0000028 |
| CYP1B1 | 0.6166398 | 0.0000621 | 0.4741313 | 0.0038394 |
| DEPDC1B | 0.4800438 | 0.0030475 | 0.5631918 | 0.0004367 |
| DHFR | 0.2781486 | 0.1004677 | 0.2386100 | 0.1606426 |
| DOCK4 | 0.5874598 | 0.0001653 | 0.6000000 | 0.0001487 |
| DTL | 0.5915818 | 0.0001448 | 0.6525097 | 0.0000255 |
| E2F7 | -0.0322089 | 0.8520673 | 0.1006435 | 0.5578492 |
| EVI5 | 0.5886082 | 0.0001594 | 0.5830116 | 0.0002482 |
| FANCD2 | 0.3958319 | 0.0168623 | 0.3580438 | 0.0326625 |
| FIGNL1 | 0.8522241 | 0.0000000 | 0.7703990 | 0.0000004 |
| FN1 | 0.7287875 | 0.0000005 | 0.7760618 | 0.0000004 |
| GATA3 | 0.0302286 | 0.8610715 | 0.0350064 | 0.8391233 |
| GBP2 | 0.8357975 | 0.0000000 | 0.7683398 | 0.0000005 |
| GDPD1 | 0.2374269 | 0.1632220 | 0.3163449 | 0.0606105 |
| GINS1 | 0.7350265 | 0.0000003 | 0.7564994 | 0.0000006 |
| GINS2 | 0.4588316 | 0.0048831 | 0.5516088 | 0.0005984 |
| GINS4 | 0.4788773 | 0.0031300 | 0.4972973 | 0.0022961 |
| GLIPR1 | 0.7661498 | 0.0000001 | 0.7467181 | 0.0000008 |
| GNG2 | 0.5204512 | 0.0011382 | 0.5351351 | 0.0009200 |
| HAUS5 | 0.2939769 | 0.0818065 | 0.3039897 | 0.0718292 |
| HAUS6 | 0.6516883 | 0.0000167 | 0.6555985 | 0.0000228 |
| HMMR | 0.3453562 | 0.0391161 | 0.3377091 | 0.0445503 |
| IDS | 0.6817044 | 0.0000047 | 0.6872587 | 0.0000069 |
| IMPA2 | 0.7818353 | 0.0000000 | 0.7505792 | 0.0000007 |
| IRS1 | 0.6717763 | 0.0000073 | 0.6548263 | 0.0000234 |
| ISG20 | 0.7728744 | 0.0000000 | 0.7492921 | 0.0000008 |
| ITGB8 | 0.8554376 | 0.0000000 | 0.8512227 | 0.0000000 |
| KIF15 | 0.4752949 | 0.0033955 | 0.5603604 | 0.0004721 |
| KIF4A | 0.5553371 | 0.0004386 | 0.6298584 | 0.0000565 |
| KIF5C | 0.3785501 | 0.0228097 | 0.1534106 | 0.3702841 |
| LAMC2 | 0.7614318 | 0.0000001 | 0.7544402 | 0.0000007 |
| LARP6 | 0.1026777 | 0.5512404 | 0.2481338 | 0.1442171 |
| LAT2 | 0.7175605 | 0.0000008 | 0.6489060 | 0.0000291 |
| LEPROT | 0.3789885 | 0.0226398 | 0.5673102 | 0.0003894 |
| LMNB1 | 0.7143442 | 0.0000010 | 0.7016731 | 0.0000039 |
| MAP2 | 0.7016620 | 0.0000019 | 0.6875161 | 0.0000068 |
| MASTL | 0.3748533 | 0.0242851 | 0.4888031 | 0.0027830 |
| MCM10 | 0.5129243 | 0.0013800 | 0.6159588 | 0.0000897 |
| MCM2 | 0.6661975 | 0.0000092 | 0.6213642 | 0.0000751 |
| MDFIC | 0.6289632 | 0.0000398 | 0.6746461 | 0.0000112 |
| MEX3D | 0.7572126 | 0.0000001 | 0.7274131 | 0.0000015 |
| MYB | 0.5081444 | 0.0015561 | 0.5240669 | 0.0012145 |
| NCAPD2 | 0.6670332 | 0.0000089 | 0.6728443 | 0.0000120 |
| NCAPG | 0.5825742 | 0.0001930 | 0.6584299 | 0.0000205 |
| NDC80 | 0.6006818 | 0.0001073 | 0.6270270 | 0.0000622 |
| NFIA | 0.4302006 | 0.0088221 | 0.5904762 | 0.0001988 |
| NRP1 | 0.7505821 | 0.0000001 | 0.7086229 | 0.0000030 |
| PLAT | 0.3699469 | 0.0263645 | 0.5541828 | 0.0005585 |
| PLAUR | 0.6794730 | 0.0000052 | 0.6918919 | 0.0000057 |
| PLK1 | 0.6417464 | 0.0000246 | 0.6465894 | 0.0000316 |
| PLSCR4 | 0.7496650 | 0.0000001 | 0.6929215 | 0.0000055 |
| PRMT3 | 0.6358680 | 0.0000308 | 0.5711712 | 0.0003492 |
| PTHLH | 0.3954723 | 0.0169712 | 0.5129987 | 0.0015896 |
| PTP4A1 | 0.8176504 | 0.0000000 | 0.7804376 | 0.0000004 |
| QKI | 0.5480791 | 0.0005395 | 0.5794080 | 0.0002757 |
| RABL3 | 0.4759994 | 0.0033418 | 0.4476190 | 0.0066532 |
| RAD51 | 0.4546749 | 0.0053375 | 0.4087516 | 0.0139154 |
| RAD51AP1 | 0.6413341 | 0.0000250 | 0.6347490 | 0.0000478 |
| RASGRP1 | 0.6429813 | 0.0000235 | 0.5503218 | 0.0006193 |
| RRAS2 | 0.7178450 | 0.0000008 | 0.6797941 | 0.0000092 |
| SEC61A2 | -0.0103213 | 0.9523597 | 0.0447876 | 0.7948741 |
| SFXN1 | 0.4616637 | 0.0045930 | 0.4702703 | 0.0041698 |
| SKA1 | 0.2445001 | 0.1506769 | 0.2692407 | 0.1122940 |
| SLC16A1 | 0.7846973 | 0.0000000 | 0.7364221 | 0.0000011 |
| SLC1A1 | 0.4017980 | 0.0151381 | 0.4064350 | 0.0145063 |
| SNAP25 | 0.6343491 | 0.0000326 | 0.6751609 | 0.0000110 |
| SOX4 | 0.8226564 | 0.0000000 | 0.7835264 | 0.0000003 |
| ST3GAL5 | 0.8085140 | 0.0000000 | 0.7528958 | 0.0000007 |
| STIL | 0.3308944 | 0.0486982 | 0.2555985 | 0.1322389 |
| SYT1 | 0.6669776 | 0.0000089 | 0.7263835 | 0.0000016 |
| SYTL2 | 0.7913271 | 0.0000000 | 0.7945946 | 0.0000003 |
| TCF19 | 0.5360917 | 0.0007517 | 0.4885457 | 0.0027991 |
| TGFB1I1 | 0.7052674 | 0.0000016 | 0.7302445 | 0.0000014 |
| TK1 | 0.3774197 | 0.0232527 | 0.4344916 | 0.0086109 |
| TMEM156 | 0.6652190 | 0.0000096 | 0.6548263 | 0.0000234 |
| TMEM194A | 0.5600871 | 0.0003821 | 0.4725869 | 0.0039687 |
| TMPO | 0.6241085 | 0.0000475 | 0.6002574 | 0.0001475 |
| TOX2 | 0.5508294 | 0.0004991 | 0.5348777 | 0.0009261 |
| TTC9 | 0.5028263 | 0.0017750 | 0.4625483 | 0.0049057 |
| TTPAL | 0.3685422 | 0.0269862 | 0.2486486 | 0.1433661 |
| ZBTB41 | 0.5139528 | 0.0013445 | 0.5552124 | 0.0005432 |
| ZEB2 | 0.5075588 | 0.0015790 | 0.4620335 | 0.0049586 |
| ZNF559 | 0.5357793 | 0.0007581 | 0.4882883 | 0.0028152 |
| ZNF569 | 0.5704439 | 0.0002807 | 0.5719434 | 0.0003417 |

### b. Comparison of the PC1 scores derived from the 74 gene signature and the original 106 genes (Figure S3 and S4).

The correlation was strong between the 74 genes and the 106 genes regardless of tissue type (FF: r=0.99-0.99 in Figure S3B; FFPE: r=0.98-0.99 in Figure S3B). The percentage of total variation was similar (28.62-30.18%; Figure S4A-D). These results indicate the remaining 74 genes reflected the original biology of the larger list.

Figure S3
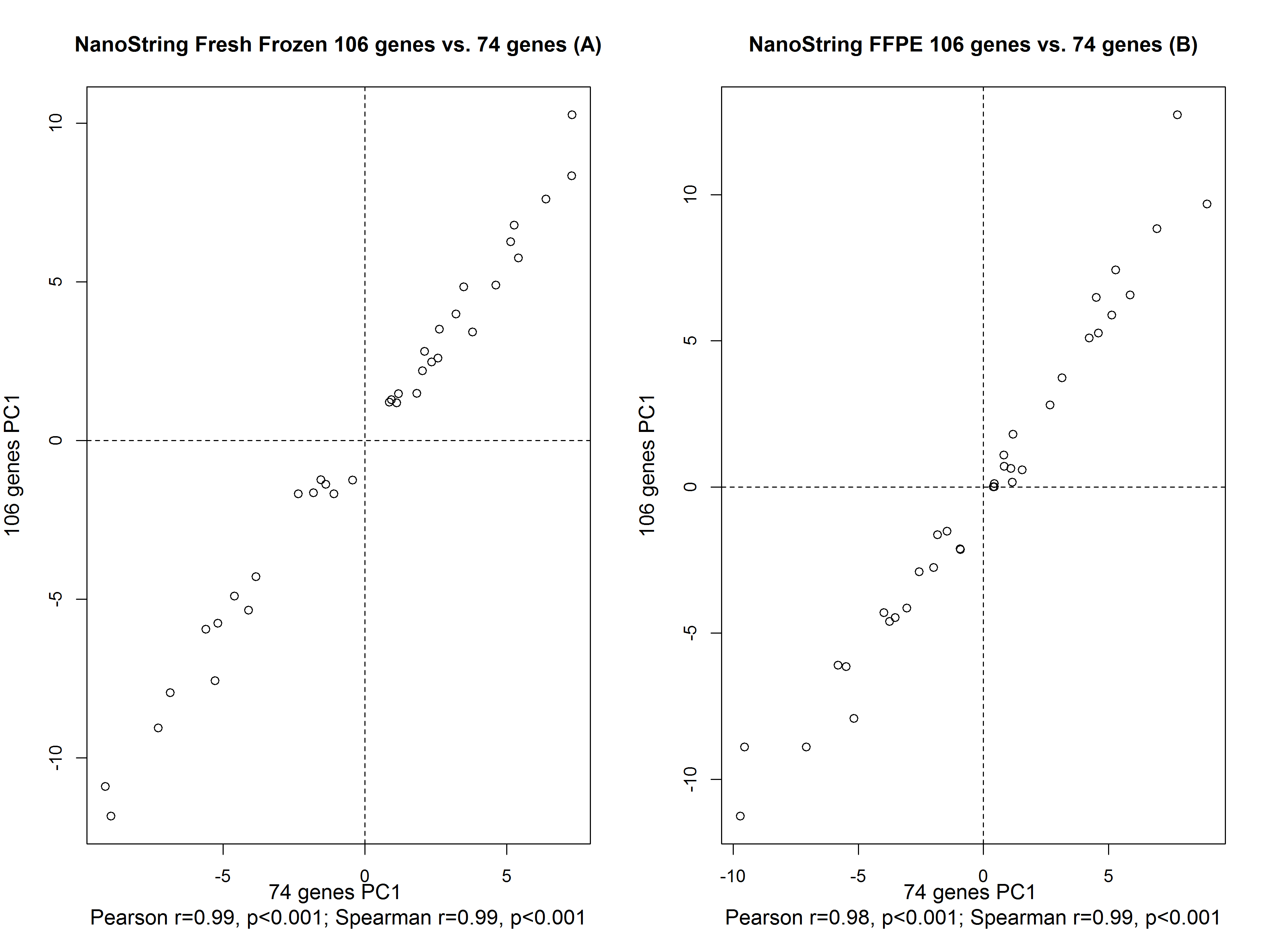


Figure S4
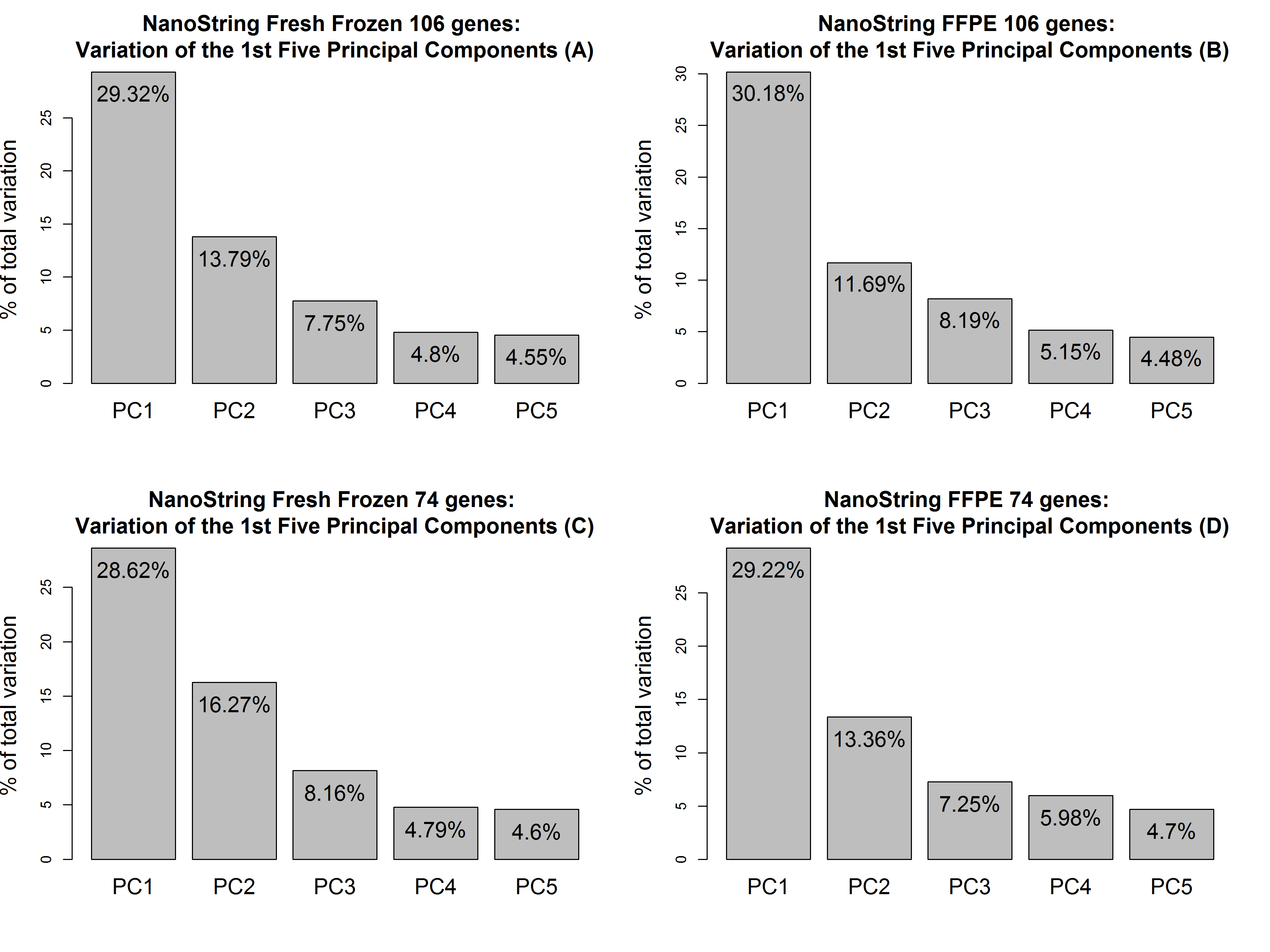


### c. Correlation analysis of PC1 Score among FF in microarray and FF and FFPE in NanoString (Figure S5).

In Figure S5, the correlation of PC1 loading coefficients was high and comparable between NanoString and microarray (r=0.84-0.86; Figure S5B) and between in NanoString between FFPE and FF (r=0.87-0.88; Figure S5D). However, the correlation of PC1 score was higher in FF between NanoString and microarray (r=0.76-0.78; Figure S5A) than in NanoString between FFPE and FF (r=0.64-0.65; Figure S5C), indicating a larger variation due to the tissue type than the platform. The correlation of PC1 score further became weaker between FF in microarray and FFPE in NanoString (r=0.3-0.43; Figure S5E), suggesting a synergistic effect to variation by the combination of different tissue type and different platform. The correlation of the corresponding PC1 loading coefficients was also smaller (r=0.69-0.7; Figure S5F).

Figure S5
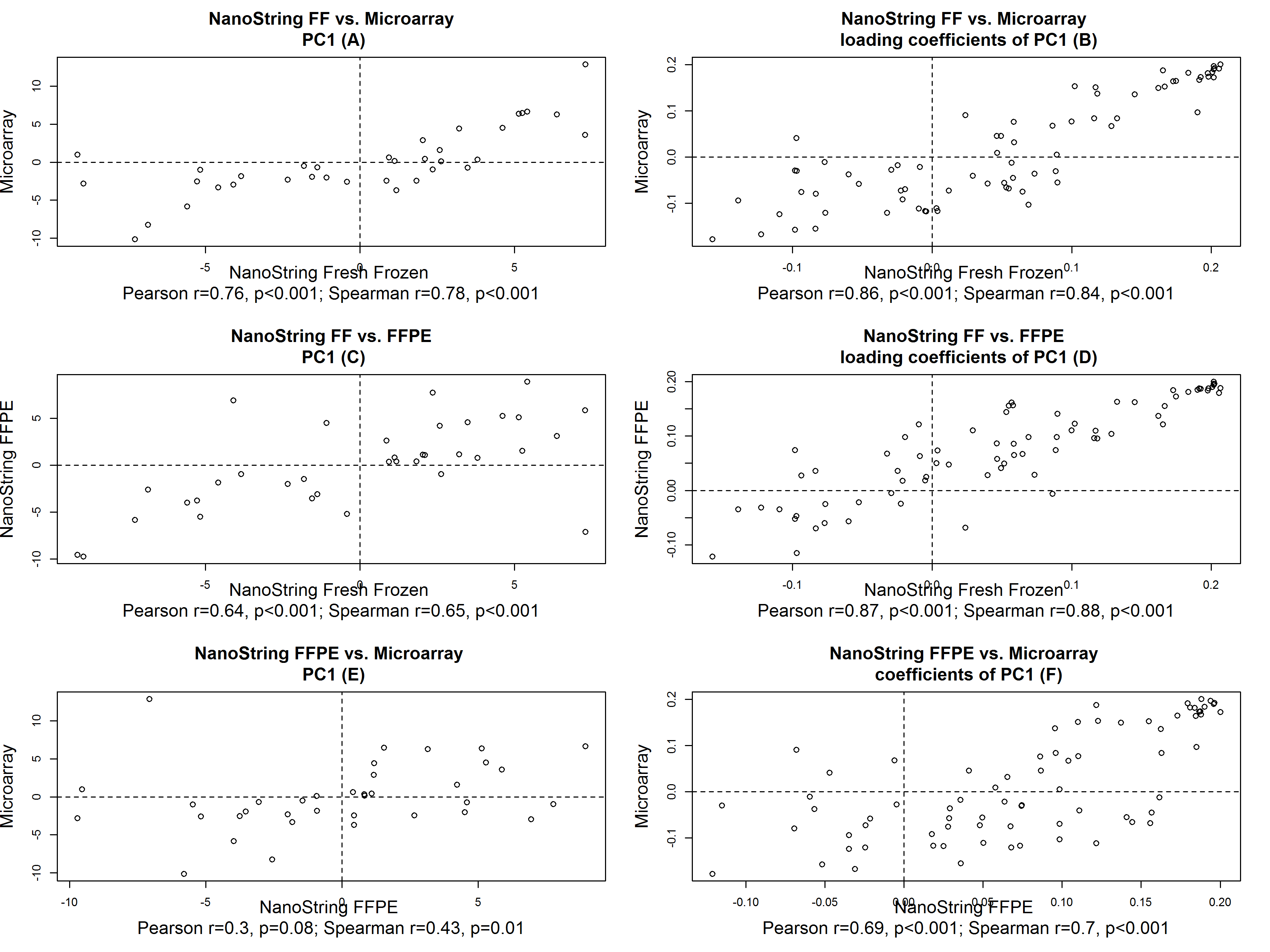


## E2F scoring system

The E2F score was calculated based on the PC1 loading coefficients (gene weights). To adjust for variation due to tissue types and platforms, the E2F scoring system of the 74-gene signature was developed in two platforms: FF and FFPE. The FF platform used the MLOS cohort to derive the PC1 gene weights while the FFPE platform used the MLCom cohort for derivation. Two aspects were evaluated for the E2F scoring system between the two platforms.

### a. Percentage of total variation for PC1 (Fig S6)

Figure S6
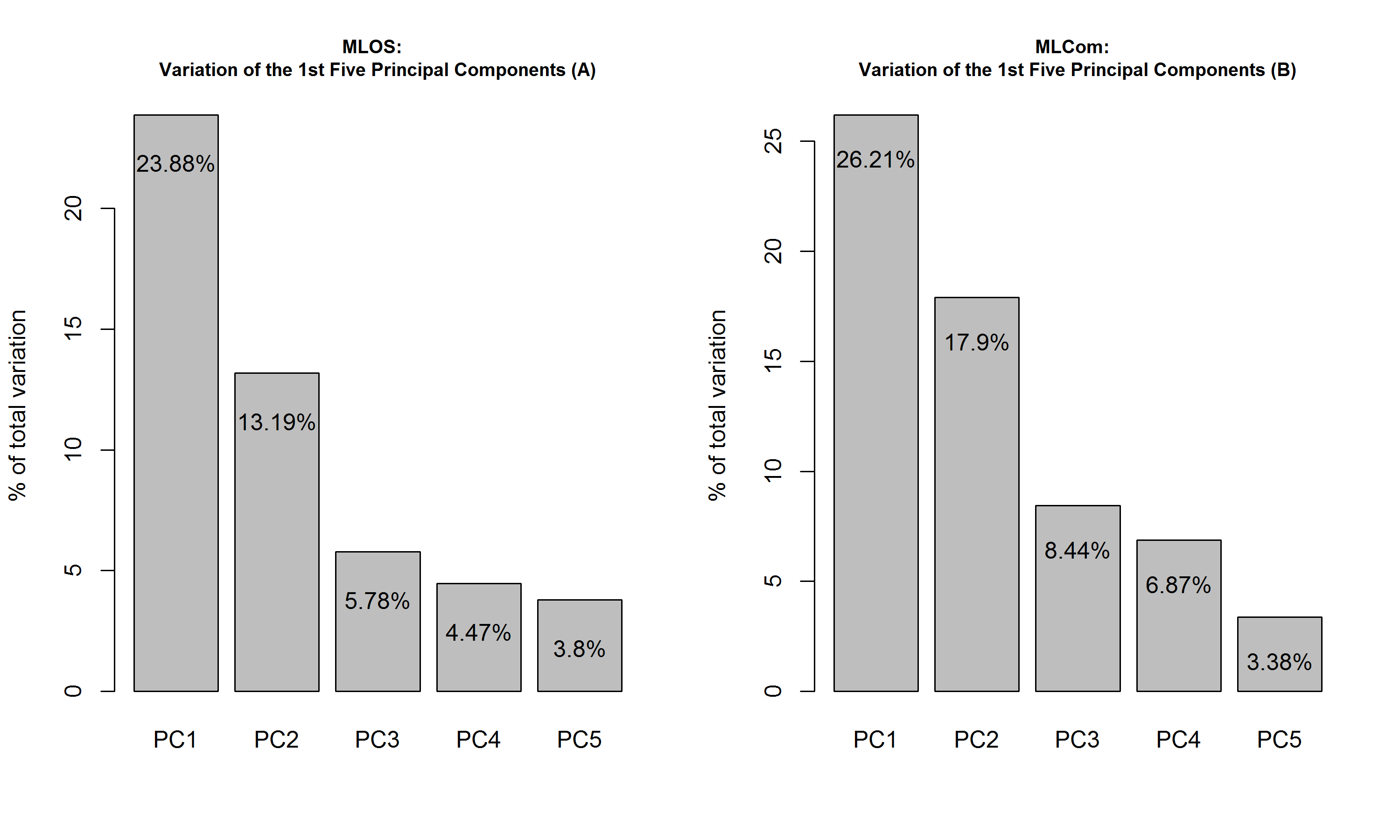


The PC1 had a comparable percentage of total variation (23.88-26.21%; Figure S6) in both platforms.

### b. Correlation of gene weights between the two platforms (Table S8 and Fig S7)

Correlation was weak (r=0.25-0.28; Figure S7). However, both platforms gave a similar range of gene weights (-0.165 to 0.223 in microarray and -0.165 to 0.21 in NanoString). Moreover, majority of gene weights (62.16%) had the same positive or negative direction between the two platforms even with various confounding factors (tissue type and platform) trying to distort the concordance.

Figure S7
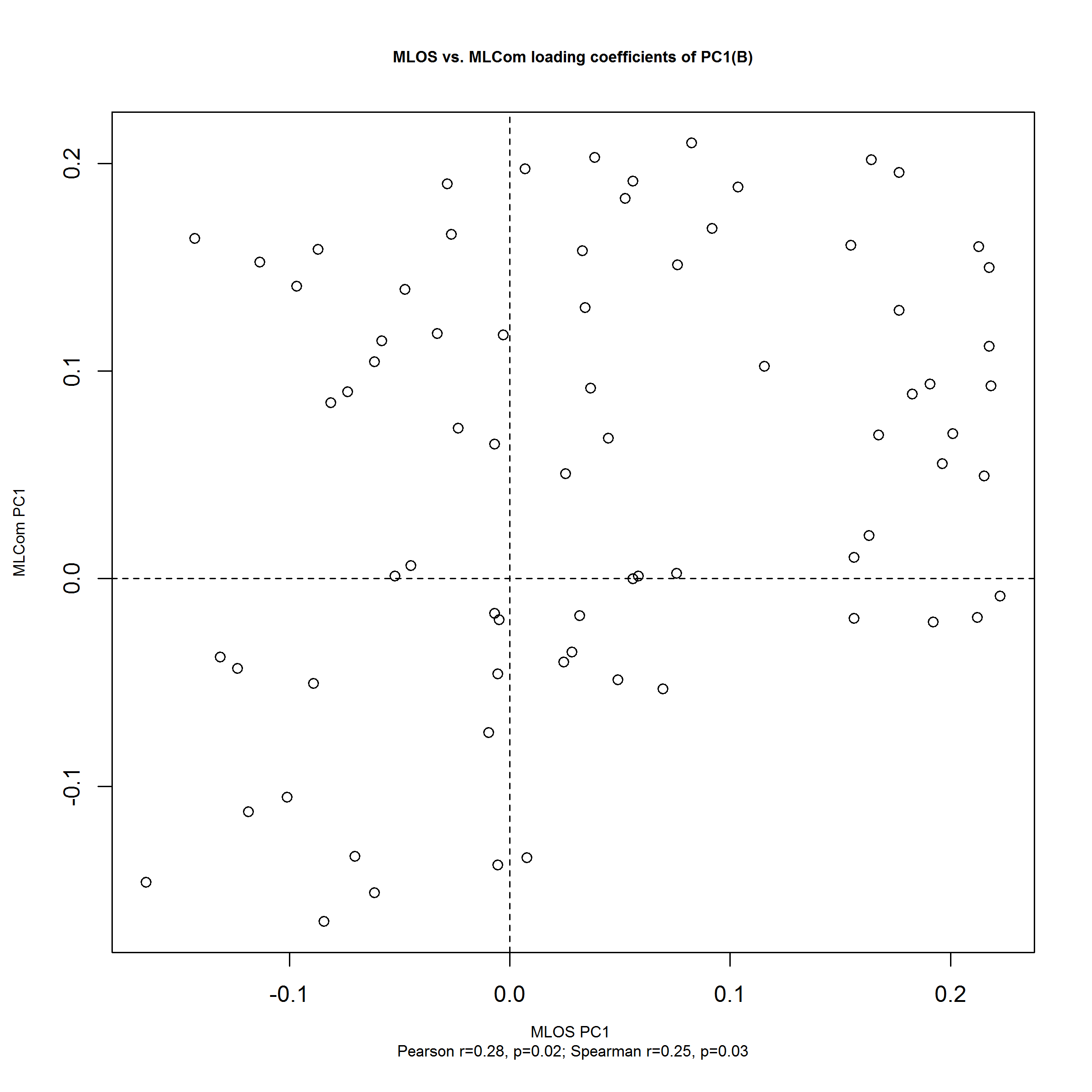


Table S8: Loading coefficients of PC1 in MLOS and MLCom cohorts

| Gene | MLOS_PC1 | MLCom_PC1 |
| --- | --- | --- |
| ABAT | -0.1010905 | -0.1053693 |
| ABCC6 | -0.0966461 | 0.1408491 |
| ACOX2 | -0.0842738 | -0.1651885 |
| AGPAT9 | -0.0448101 | 0.0062285 |
| ANXA1 | -0.0234442 | 0.0723667 |
| ARL14 | 0.0447506 | 0.0675834 |
| BDH2 | -0.1428950 | 0.1637960 |
| BIRC5 | 0.2176624 | 0.1496777 |
| BLM | 0.1827808 | 0.0889119 |
| BUB1B | 0.2185485 | 0.0926530 |
| CCNE2 | 0.1549286 | 0.1604561 |
| CDC6 | 0.2176120 | 0.1117077 |
| CDCA4 | 0.1631919 | 0.0207153 |
| CDK6 | 0.0523628 | 0.1830719 |
| CHST11 | 0.0282783 | -0.0354830 |
| CKS1B | 0.1767451 | 0.1291335 |
| CPM | -0.0890454 | -0.0506735 |
| CYBRD1 | -0.1650846 | -0.1463917 |
| CYP1B1 | -0.0067929 | 0.0647764 |
| DOCK4 | -0.1185291 | -0.1123287 |
| DTL | 0.2010301 | 0.0696431 |
| EVI5 | -0.1134075 | 0.1522570 |
| FIGNL1 | 0.1035669 | 0.1885991 |
| FN1 | 0.0558682 | -0.0001356 |
| GBP2 | 0.0331302 | 0.1577334 |
| GINS1 | 0.0696837 | -0.0532720 |
| GLIPR1 | -0.0054665 | -0.1380187 |
| GNG2 | -0.0812298 | 0.0846646 |
| HAUS6 | 0.0826252 | 0.2097804 |
| IDS | -0.0736091 | 0.0899071 |
| IMPA2 | -0.0054705 | -0.0458628 |
| IRS1 | 0.0490877 | -0.0488498 |
| ISG20 | 0.0342629 | 0.1304103 |
| ITGB8 | 0.0253629 | 0.0504778 |
| KIF4A | 0.2226870 | -0.0084484 |
| LAMC2 | 0.0762938 | 0.1510649 |
| LAT2 | 0.0070729 | 0.1973859 |
| LMNB1 | 0.1767536 | 0.1954229 |
| MAP2 | -0.0521407 | 0.0012231 |
| MCM10 | 0.2129807 | 0.1598036 |
| MCM2 | 0.1962904 | 0.0553125 |
| MDFIC | -0.0282216 | 0.1900267 |
| MEX3D | 0.0386346 | 0.2027269 |
| MYB | 0.0318550 | -0.0180176 |
| NCAPD2 | 0.1563597 | 0.0100330 |
| NCAPG | 0.2123537 | -0.0188984 |
| NDC80 | 0.1908375 | 0.0935457 |
| NRP1 | -0.0869296 | 0.1585023 |
| PLAUR | 0.0918000 | 0.1686459 |
| PLK1 | 0.2153638 | 0.0492623 |
| PLSCR4 | -0.1312896 | -0.0377947 |
| PRMT3 | 0.0584934 | 0.0011401 |
| PTP4A1 | 0.0367103 | 0.0917284 |
| QKI | -0.0028557 | 0.1173032 |
| RAD51AP1 | 0.1921854 | -0.0209469 |
| RASGRP1 | -0.0701790 | -0.1338729 |
| RRAS2 | -0.0580838 | 0.1145004 |
| SLC16A1 | 0.1155689 | 0.1022164 |
| SNAP25 | -0.0094575 | -0.0743411 |
| SOX4 | 0.0559584 | 0.1913382 |
| ST3GAL5 | -0.1234971 | -0.0432589 |
| SYT1 | 0.0757941 | 0.0023706 |
| SYTL2 | -0.0473995 | 0.1392201 |
| TCF19 | 0.1642371 | 0.2016309 |
| TGFB1I1 | -0.0329194 | 0.1178857 |
| TMEM156 | 0.0244818 | -0.0403670 |
| TMEM194A | 0.1564160 | -0.0193204 |
| TMPO | 0.1675399 | 0.0691305 |
| TOX2 | -0.0068597 | -0.0169027 |
| TTC9 | 0.0078763 | -0.1343876 |
| ZBTB41 | -0.0264331 | 0.1657864 |
| ZEB2 | -0.0614269 | 0.1042986 |
| ZNF559 | -0.0612469 | -0.1512738 |
| ZNF569 | -0.0047067 | -0.0199321 |

## Post-hoc evaluation of E2F median-cutoff classification from training to validation cohorts (Table S9, S10, S11, S12 and Figure S8-S9)

The median-cutoff of the E2F score was derived from the two training cohorts: the MLOS cohort for the fresh frozen cohorts and the the MLCom cohort for the FFPE cohorts. The median-cutoff score was then used to classify patients into low and high E2F groups. In the fresh frozen cohorts, the median-cutoff of the E2F score (derived from the MLOS cohort) was used to classify patients into low and high E2F groups in the MLOS cohort. As expected, the classification by the median-cutoff E2F score was significantly associated with OS in non-ACT patients with poor OS in high E2F group (Figure S8A). The classification with the same median cutoff (i.e., the median cutoff from the MLOS cohort) in the validation cohorts (MCLA, TCGA, JBR10, and LCBRN) also showed a significant separation between the low and high E2F groups in non-ACT patients (p<0.05; Figure S8A). In comparison, while classification by other cutoffs (25th-75th percentiles) had a significant association in the training cohort (MLOS), significant level most occurred in cutoff with the range of 50th-60th percentiles in all the three validation cohorts (Figure S8A). In evaluating the JBR10 cohorts (JBR10.NSCLC and JBR10.AD) for the predictive effect, again the significant level (for treatment effect in the high E2F group and for interaction effect) most happened in the interval of 50th-60th percentiles either in OS (Figure S8B) or DSS (Figure S8C).

Similarly, in the FFPE cohorts, the median-cutoff E2F score was derived from the MLCom cohort (the training set). In Figure S8A-B, the median-cutoff classification was able to significantly separate the low and high E2F groups in non-ACT patients of the MLCom cohort in terms of OS and PFS. For the validation cohorts, the same median-cutoff classification showed a significant level in the NATCH cohort. In contrast, classification by other cutoffs performed differently. The significant level most occurred in cutoff with the range of 40th-60th percentiles (for OS; Figure S8A) and the range of 25th-75th percentiles (for PFS; Figure S8B) in the MLCom cohort, but only happened around median range in the NATCH cohort for both OS and PFS. For evaluation of the predictive effect in the NATCH cohort, the significant level (for treatment effect in the high E2F group) happened around the median range in PFS (Figure S8C). The p values around the median range in OS were the smallest, but not below 0.05 (Figure S8D).

By taking these results for consideration, it suggests the median-cutoff E2F score as a reasonable choice for risk classification to better predict prognosis and ACT effect. Table S9 - S12 and Figure S8-S9 summarized the results.

Figure S8

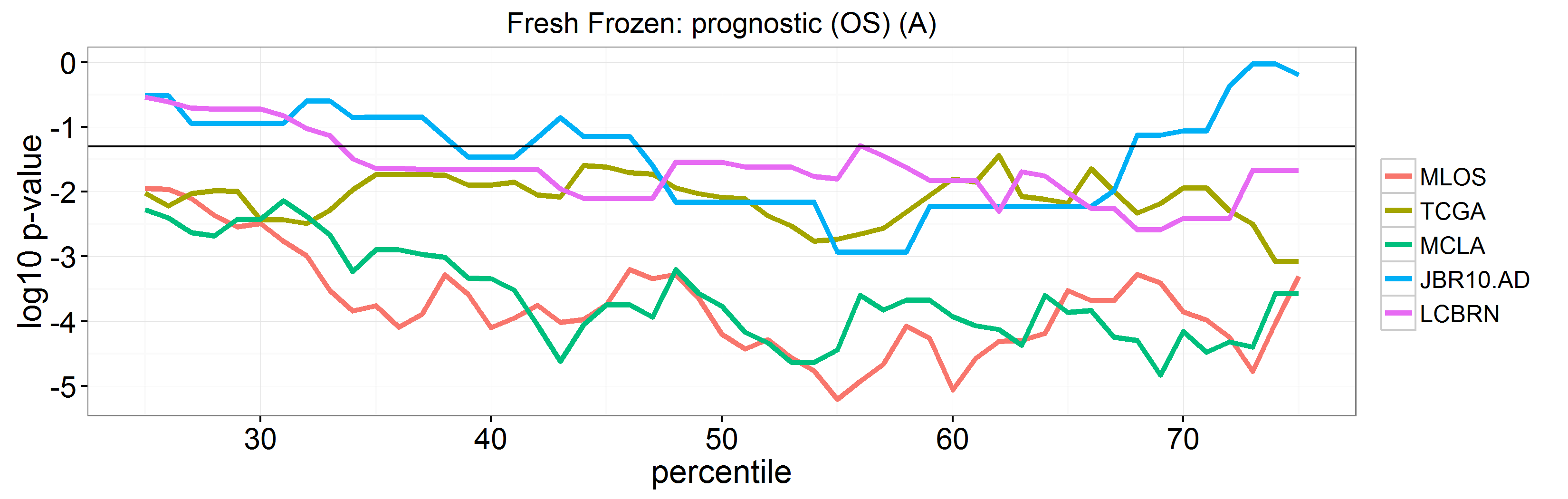

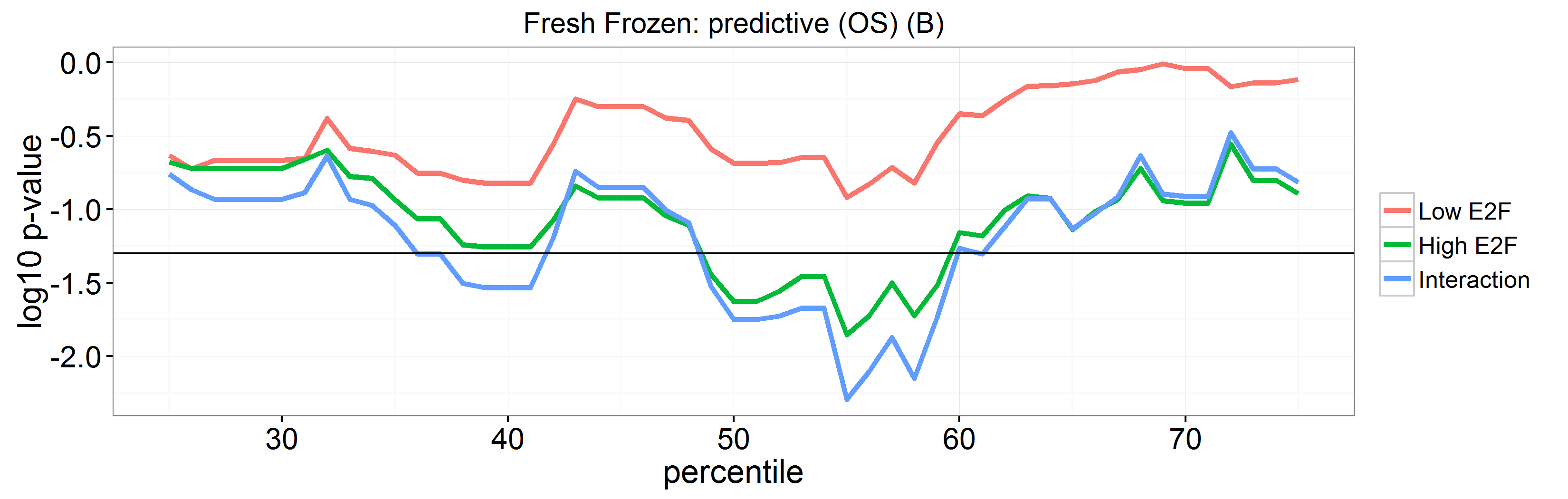

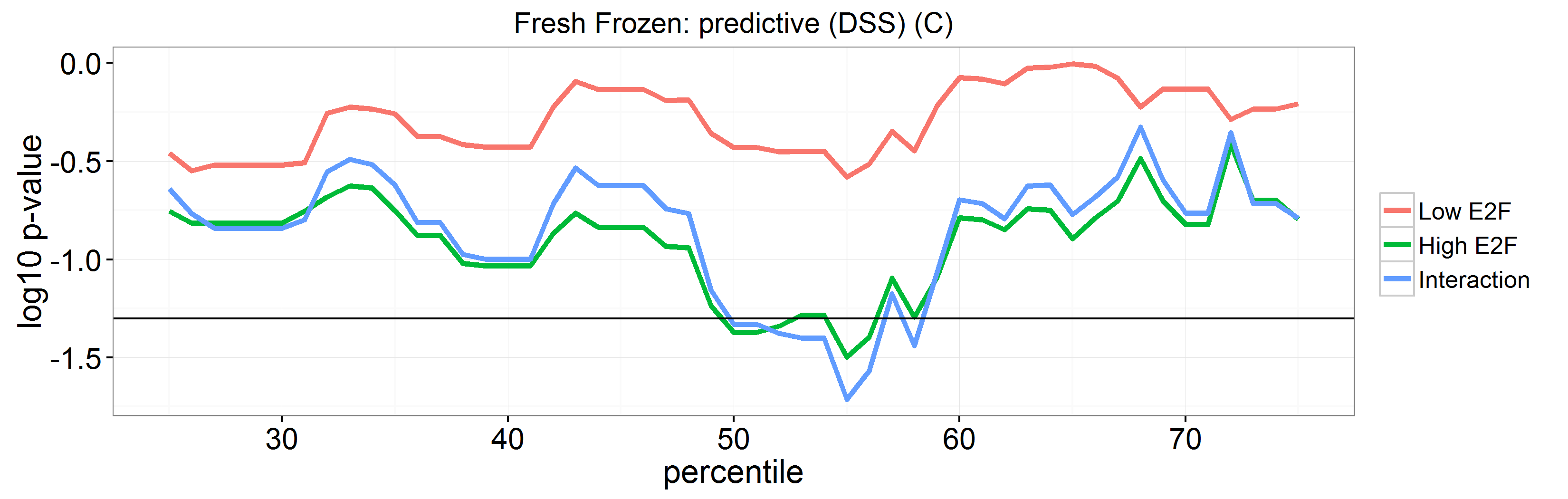


Figure S9

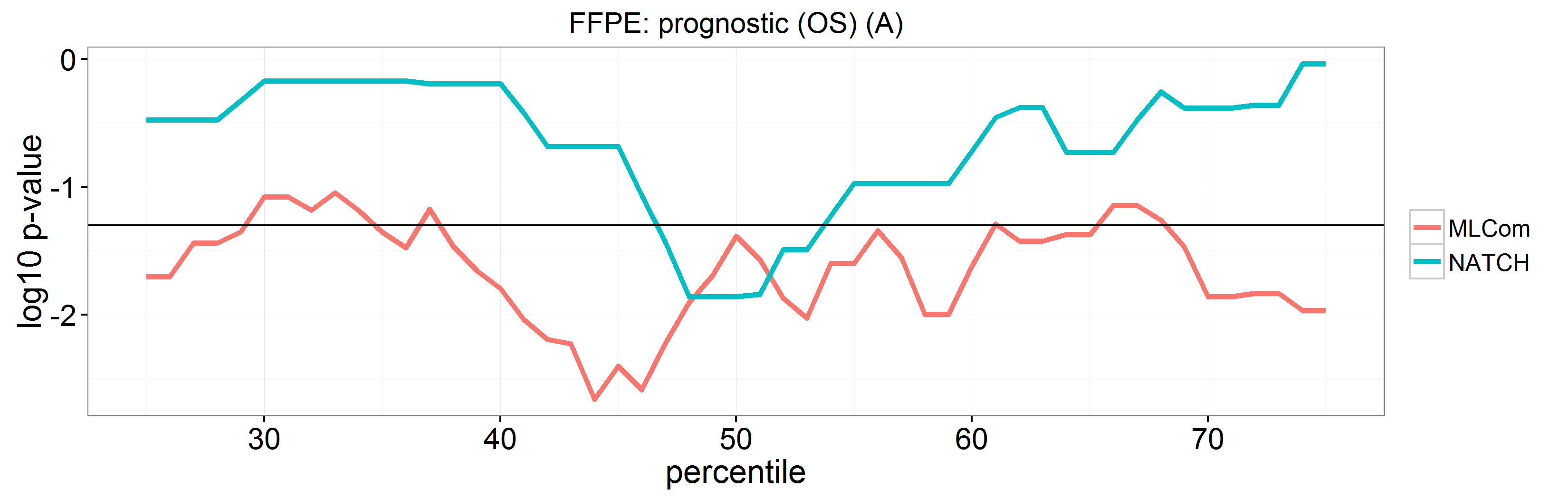

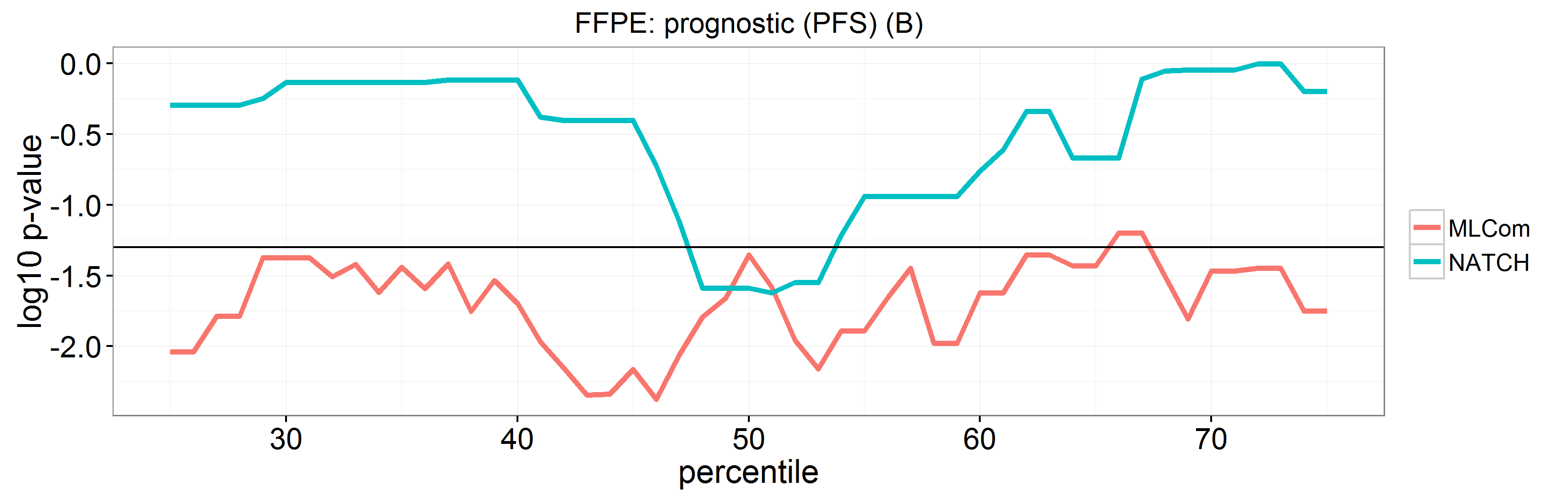

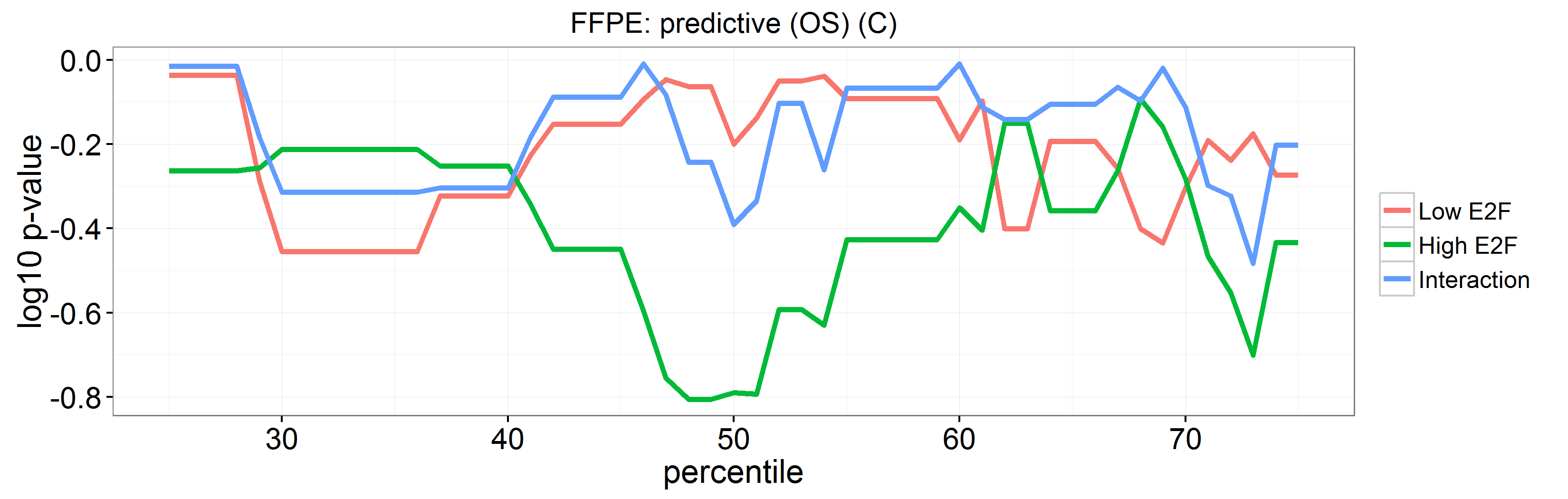

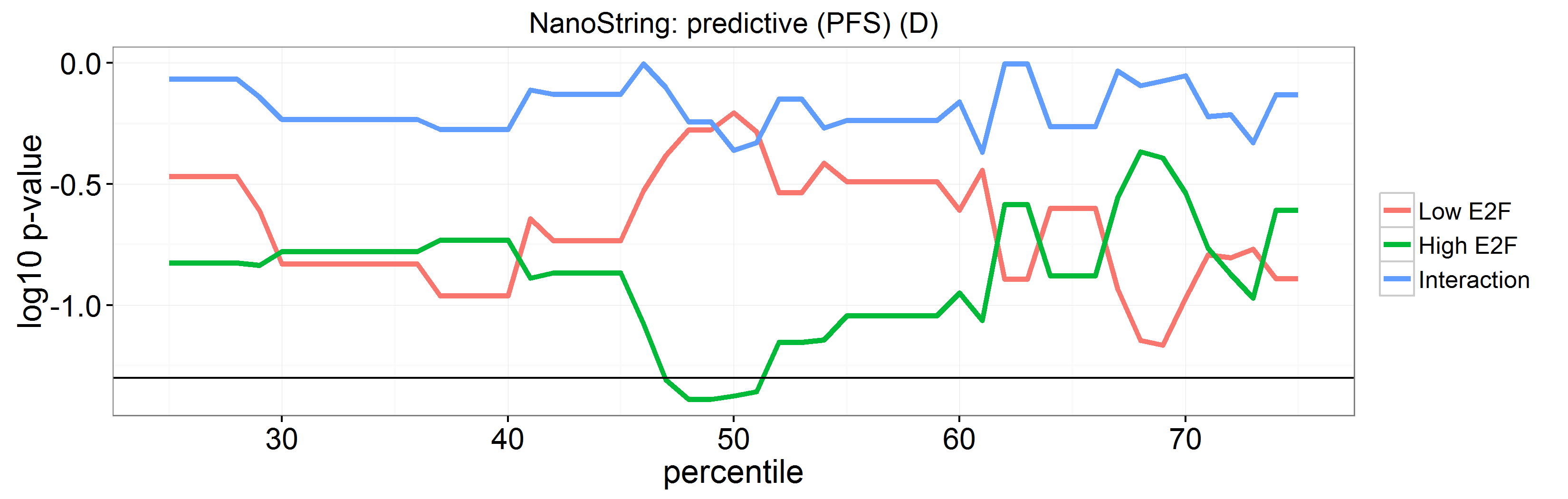


Table S9: Evaluation of E2F score cutpoints in FF cohorts (prognostic effects)

| percentile | cutpoint | MLOS | TCGA | MCLA | JBR10 | LCBRN |
| --- | --- | --- | --- | --- | --- | --- |
| 25^th^ | -3.2 | 0.0112908 | 0.0095579 | 0.0052922 | 0.3058929 | 0.2928693 |
| 26^th^ | -3.122 | 0.010996 | 0.006019 | 0.0039351 | 0.3058929 | 0.2445851 |
| 27^th^ | -2.96 | 0.0079921 | 0.0093416 | 0.0023362 | 0.114855 | 0.1961848 |
| 28^th^ | -2.832 | 0.0043304 | 0.0103466 | 0.0020992 | 0.114855 | 0.1903627 |
| 29^th^ | -2.782 | 0.0028744 | 0.0103034 | 0.0037479 | 0.114855 | 0.1903627 |
| 30^th^ | -2.728 | 0.0032207 | 0.0037344 | 0.0037479 | 0.114855 | 0.1903627 |
| 31th | -2.638 | 0.0017207 | 0.0037109 | 0.0073414 | 0.114855 | 0.1511692 |
| 32th | -2.482 | 0.0010228 | 0.0032547 | 0.0041458 | 0.2530853 | 0.0961902 |
| 33th | -2.351 | 0.0003007 | 0.0052138 | 0.0021626 | 0.2530853 | 0.0739212 |
| 34^th^ | -2.145 | 0.0001446 | 0.0107538 | 0.0005891 | 0.1405721 | 0.0321532 |
| 35^th^ | -1.992 | 0.0001749 | 0.0185637 | 0.0012764 | 0.143467 | 0.0231118 |
| 36^th^ | -1.926 | 0.0000804 | 0.0184911 | 0.0012764 | 0.143467 | 0.0231118 |
| 37^th^ | -1.889 | 0.0001286 | 0.0184911 | 0.0010704 | 0.143467 | 0.0221248 |
| 38^th^ | -1.768 | 0.0005185 | 0.0181314 | 0.0009698 | 0.0699488 | 0.0221248 |
| 39^th^ | -1.704 | 0.0002588 | 0.0127118 | 0.0004631 | 0.0345029 | 0.0221248 |
| 40^th^ | -1.642 | 0.0000795 | 0.0127118 | 0.0004542 | 0.0345029 | 0.0221248 |
| 41th | -1.585 | 0.0001115 | 0.0140704 | 0.0003054 | 0.0345029 | 0.0221248 |
| 42th | -1.463 | 0.0001761 | 0.0088758 | 0.0000874 | 0.0695068 | 0.0221248 |
| 43th | -1.341 | 0.0000958 | 0.008326 | 0.0000239 | 0.1413438 | 0.0111575 |
| 44^th^ | -1.09 | 0.0001068 | 0.0256049 | 0.0000883 | 0.071568 | 0.007954 |
| 45^th^ | -1.029 | 0.0001794 | 0.02437 | 0.000179 | 0.071568 | 0.007954 |
| 46^th^ | -0.966 | 0.0006269 | 0.019931 | 0.000179 | 0.071568 | 0.007954 |
| 47^th^ | -0.906 | 0.0004588 | 0.0189327 | 0.0001147 | 0.0256393 | 0.007954 |
| 48^th^ | -0.71 | 0.0005306 | 0.0115255 | 0.0006272 | 0.0069669 | 0.0284954 |
| 49^th^ | -0.575 | 0.0002259 | 0.0093948 | 0.0002687 | 0.0069669 | 0.0284954 |
| 50^th^ | -0.504 | 0.000063 | 0.0082466 | 0.0001719 | 0.0069669 | 0.0284954 |
| 51th | -0.408 | 0.0000371 | 0.0077415 | 0.0000671 | 0.0069669 | 0.0240885 |
| 52th | -0.292 | 0.0000518 | 0.0042768 | 0.0000465 | 0.0069669 | 0.0240885 |
| 53th | -0.22 | 0.0000275 | 0.0029616 | 0.0000231 | 0.0069669 | 0.0240885 |
| 54^th^ | -0.129 | 0.0000171 | 0.0017175 | 0.0000231 | 0.0069669 | 0.0172749 |
| 55^th^ | 0.1 | 0.0000062 | 0.0018603 | 0.0000357 | 0.0011658 | 0.0158833 |
| 56^th^ | 0.257 | 0.0000118 | 0.0022429 | 0.0002508 | 0.0011658 | 0.0521689 |
| 57^th^ | 0.389 | 0.0000218 | 0.0027159 | 0.0001483 | 0.0011658 | 0.0359245 |
| 58^th^ | 0.544 | 0.0000836 | 0.0048654 | 0.0002139 | 0.0011658 | 0.0237929 |
| 59^th^ | 0.683 | 0.000055 | 0.0087925 | 0.0002139 | 0.0059054 | 0.0150538 |
| 60^th^ | 0.823 | 0.0000087 | 0.015792 | 0.0001169 | 0.0059054 | 0.0150538 |
| 61th | 0.94 | 0.0000267 | 0.0140837 | 0.0000857 | 0.0059054 | 0.0150538 |
| 62th | 1.119 | 0.0000491 | 0.0362978 | 0.0000749 | 0.0059054 | 0.0050677 |
| 63th | 1.317 | 0.0000501 | 0.0085505 | 0.0000424 | 0.0059054 | 0.0203654 |
| 64^th^ | 1.469 | 0.0000655 | 0.0077296 | 0.0002501 | 0.0059054 | 0.0175479 |
| 65^th^ | 1.722 | 0.0002975 | 0.0067431 | 0.000138 | 0.0059054 | 0.0097922 |
| 66^th^ | 1.921 | 0.0002078 | 0.0228388 | 0.0001469 | 0.0059054 | 0.0055952 |
| 67^th^ | 2.006 | 0.0002078 | 0.0101551 | 0.0000572 | 0.0104164 | 0.0055952 |
| 68^th^ | 2.146 | 0.0005306 | 0.0046605 | 0.0000502 | 0.0759254 | 0.0025863 |
| 69^th^ | 2.3 | 0.0003936 | 0.0065499 | 0.0000148 | 0.0759254 | 0.0025863 |
| 70^th^ | 2.508 | 0.0001402 | 0.0114322 | 0.0000695 | 0.0882138 | 0.0039 |
| 71th | 2.539 | 0.0001044 | 0.0114322 | 0.0000331 | 0.0882138 | 0.0039 |
| 72th | 2.706 | 0.0000564 | 0.0051221 | 0.0000481 | 0.4304957 | 0.0039 |
| 73th | 3.055 | 0.0000168 | 0.0031691 | 0.00004 | 0.9584996 | 0.0214212 |
| 74^th^ | 3.222 | 0.0000945 | 0.0008367 | 0.0002673 | 0.9584996 | 0.0214212 |
| 75^th^ | 3.267 | 0.0004963 | 0.0008349 | 0.0002673 | 0.642442 | 0.0214212 |

Table S10: Evaluation of E2F score cutpoints in FF cohorts (predictive effects)

| rname | cutoff | low.E2F.OS | High.E2F.OS | int.p.OS | low.E2F.DSS | High.E2F.DSS | int.p.DSS |
| --- | --- | --- | --- | --- | --- | --- | --- |
|  |  |  |  |  |  |  |  |
| 25th | -3.2 | 0.2322411 | 0.2101074 | 0.1747139 | 0.3474415 | 0.1760146 | 0.2283245 |
| 26th | -3.122 | 0.1889988 | 0.1899784 | 0.1360502 | 0.2825408 | 0.1535256 | 0.1710374 |
| 27th | -2.96 | 0.2154809 | 0.1910775 | 0.1176543 | 0.3020283 | 0.153055 | 0.144361 |
| 28th | -2.832 | 0.2154809 | 0.1910775 | 0.1176543 | 0.3020283 | 0.153055 | 0.144361 |
| 29th | -2.782 | 0.2154809 | 0.1910775 | 0.1176543 | 0.3020283 | 0.153055 | 0.144361 |
| 30th | -2.728 | 0.2154809 | 0.1910775 | 0.1176543 | 0.3020283 | 0.153055 | 0.144361 |
| 31th | -2.638 | 0.222411 | 0.2188578 | 0.1306494 | 0.3113993 | 0.175641 | 0.1595892 |
| 32th | -2.482 | 0.4166979 | 0.2522796 | 0.2311367 | 0.5538643 | 0.2082519 | 0.2803282 |
| 33th | -2.351 | 0.2605129 | 0.1680749 | 0.1176757 | 0.5964865 | 0.2360969 | 0.3240653 |
| 34th | -2.145 | 0.2496916 | 0.1629337 | 0.1066111 | 0.5851125 | 0.2318862 | 0.3039821 |
| 35th | -1.992 | 0.2343654 | 0.1161632 | 0.0778928 | 0.5527813 | 0.1769406 | 0.2398923 |
| 36th | -1.926 | 0.1768724 | 0.0864587 | 0.049823 | 0.4227324 | 0.1323684 | 0.1542197 |
| 37th | -1.889 | 0.1768724 | 0.0864587 | 0.049823 | 0.4227324 | 0.1323684 | 0.1542197 |
| 38th | -1.768 | 0.1575741 | 0.0574954 | 0.0314543 | 0.3845959 | 0.0954414 | 0.1062722 |
| 39th | -1.704 | 0.1512352 | 0.0555804 | 0.0293371 | 0.3734055 | 0.09262 | 0.1002857 |
| 40th | -1.642 | 0.1512352 | 0.0555804 | 0.0293371 | 0.3734055 | 0.09262 | 0.1002857 |
| 41th | -1.585 | 0.1512352 | 0.0555804 | 0.0293371 | 0.3734055 | 0.09262 | 0.1002857 |
| 42th | -1.463 | 0.2782894 | 0.0841808 | 0.0642733 | 0.597128 | 0.1356728 | 0.191333 |
| 43th | -1.341 | 0.5669972 | 0.1440317 | 0.1820185 | 0.8063112 | 0.1724439 | 0.2928018 |
| 44th | -1.09 | 0.5005028 | 0.119839 | 0.1414275 | 0.7320434 | 0.1459189 | 0.2375357 |
| 45th | -1.029 | 0.5005028 | 0.119839 | 0.1414275 | 0.7320434 | 0.1459189 | 0.2375357 |
| 46th | -0.966 | 0.5005028 | 0.119839 | 0.1414275 | 0.7320434 | 0.1459189 | 0.2375357 |
| 47th | -0.906 | 0.4205784 | 0.090295 | 0.0984648 | 0.6444237 | 0.1164155 | 0.1807377 |
| 48th | -0.71 | 0.4028142 | 0.0777706 | 0.0814269 | 0.6498786 | 0.1143631 | 0.1714371 |
| 49th | -0.575 | 0.2576484 | 0.0361394 | 0.0299864 | 0.4397627 | 0.0580921 | 0.0697907 |
| 50th | -0.504 | 0.2070023 | 0.0236718 | 0.0178533 | 0.3714228 | 0.0424406 | 0.046779 |
| 51th | -0.408 | 0.2070023 | 0.0236718 | 0.0178533 | 0.3714228 | 0.0424406 | 0.046779 |
| 52th | -0.292 | 0.2086588 | 0.0276227 | 0.018691 | 0.3527182 | 0.0455974 | 0.0419354 |
| 53th | -0.22 | 0.2260513 | 0.0350291 | 0.0212171 | 0.3564337 | 0.0519816 | 0.039659 |
| 54th | -0.129 | 0.2260513 | 0.0350291 | 0.0212171 | 0.3564337 | 0.0519816 | 0.039659 |
| 55th | 0.1 | 0.120569 | 0.0140618 | 0.0050885 | 0.2627261 | 0.0318353 | 0.0193774 |
| 56th | 0.257 | 0.1483717 | 0.0189446 | 0.0078685 | 0.3060481 | 0.040225 | 0.0270056 |
| 57th | 0.389 | 0.1926467 | 0.0316343 | 0.0133956 | 0.4485278 | 0.0804424 | 0.0667122 |
| 58th | 0.544 | 0.151097 | 0.0189396 | 0.0070777 | 0.3571513 | 0.0506758 | 0.0364377 |
| 59th | 0.683 | 0.2848858 | 0.030542 | 0.018402 | 0.6086096 | 0.081364 | 0.0864776 |
| 60th | 0.823 | 0.4499715 | 0.0695012 | 0.0545069 | 0.8451205 | 0.1625016 | 0.2017066 |
| 61th | 0.94 | 0.4352447 | 0.0660299 | 0.0498227 | 0.8291362 | 0.1589632 | 0.1920413 |
| 62th | 1.119 | 0.5570737 | 0.0986712 | 0.0762202 | 0.7853025 | 0.1419795 | 0.1606626 |
| 63th | 1.317 | 0.6898644 | 0.1240197 | 0.1178766 | 0.9427005 | 0.1809332 | 0.2367573 |
| 64th | 1.469 | 0.6960153 | 0.1194721 | 0.1184738 | 0.9513479 | 0.1776831 | 0.2396789 |
| 65th | 1.722 | 0.7159838 | 0.0726958 | 0.0737357 | 0.993458 | 0.1272818 | 0.1689103 |
| 66th | 1.921 | 0.7556126 | 0.0975966 | 0.0948106 | 0.9644855 | 0.1632939 | 0.208736 |
| 67th | 2.006 | 0.8670873 | 0.1166676 | 0.1213075 | 0.8377075 | 0.1982273 | 0.2632599 |
| 68th | 2.146 | 0.8978327 | 0.191083 | 0.2333786 | 0.5967011 | 0.326081 | 0.4714318 |
| 69th | 2.3 | 0.9839578 | 0.1147673 | 0.1273663 | 0.7380671 | 0.199198 | 0.2542037 |
| 70th | 2.508 | 0.9105961 | 0.1100817 | 0.123004 | 0.7374411 | 0.1507262 | 0.1723991 |
| 71th | 2.539 | 0.9105961 | 0.1100817 | 0.123004 | 0.7374411 | 0.1507262 | 0.1723991 |
| 72th | 2.706 | 0.6872116 | 0.2782105 | 0.333804 | 0.5171547 | 0.3851861 | 0.4412048 |
| 73th | 3.055 | 0.7267276 | 0.1575946 | 0.189286 | 0.5836821 | 0.2002766 | 0.1924004 |
| 74th | 3.222 | 0.7267276 | 0.1575946 | 0.189286 | 0.5836821 | 0.2002766 | 0.1924004 |
| 75th | 3.267 | 0.7684798 | 0.1286846 | 0.1528038 | 0.6208574 | 0.1605029 | 0.1619012 |

Table S11: Evaluation of E2F score cutpoints in FFPE cohorts (prognostic effects)

| percentile | cutpoint | MLCom_OS | NATCH_OS | MLCom_PFS | NATCH_PFS |
| --- | --- | --- | --- | --- | --- |
| 25^th^ | -2.496 | 0.019871 | 0.3334528 | 0.0091422 | 0.5057482 |
| 26^th^ | -2.477 | 0.019871 | 0.3334528 | 0.0091422 | 0.5057482 |
| 27^th^ | -2.392 | 0.0364306 | 0.3334528 | 0.016326 | 0.5057482 |
| 28^th^ | -2.304 | 0.0364306 | 0.3334528 | 0.016326 | 0.5057482 |
| 29^th^ | -2.226 | 0.0442264 | 0.4693336 | 0.0423484 | 0.5654945 |
| 30^th^ | -2.199 | 0.0834977 | 0.6727094 | 0.0423484 | 0.733685 |
| 31th | -2.172 | 0.0834977 | 0.6727094 | 0.0423484 | 0.733685 |
| 32th | -2.111 | 0.0657041 | 0.6727094 | 0.0311162 | 0.733685 |
| 33th | -2.081 | 0.0902278 | 0.6727094 | 0.0380231 | 0.733685 |
| 34^th^ | -2.009 | 0.0654155 | 0.6727094 | 0.0240075 | 0.733685 |
| 35^th^ | -1.933 | 0.0443252 | 0.6727094 | 0.0361601 | 0.733685 |
| 36^th^ | -1.86 | 0.0334898 | 0.6727094 | 0.0255148 | 0.733685 |
| 37^th^ | -1.83 | 0.0668162 | 0.6415041 | 0.0382523 | 0.7613321 |
| 38^th^ | -1.822 | 0.0345549 | 0.6415041 | 0.0176208 | 0.7613321 |
| 39^th^ | -1.812 | 0.0221434 | 0.6415041 | 0.0291646 | 0.7613321 |
| 40^th^ | -1.793 | 0.0160773 | 0.6415041 | 0.0201113 | 0.7613321 |
| 41th | -1.744 | 0.0091798 | 0.3807057 | 0.0107919 | 0.4167573 |
| 42th | -1.634 | 0.0063897 | 0.2061399 | 0.0070186 | 0.3948179 |
| 43th | -1.594 | 0.005923 | 0.2061399 | 0.0045285 | 0.3948179 |
| 44^th^ | -1.576 | 0.0021805 | 0.2061399 | 0.0045866 | 0.3948179 |
| 45^th^ | -1.563 | 0.0039734 | 0.2061399 | 0.0068624 | 0.3948179 |
| 46^th^ | -1.45 | 0.0025972 | 0.0866965 | 0.0042165 | 0.1882437 |
| 47^th^ | -1.388 | 0.0059855 | 0.0371403 | 0.0086429 | 0.0764975 |
| 48^th^ | -1.314 | 0.0125048 | 0.0138076 | 0.0161262 | 0.0257263 |
| 49^th^ | -1.276 | 0.0201247 | 0.0138076 | 0.0218449 | 0.0257263 |
| 50^th^ | -1.142 | 0.0412314 | 0.0138076 | 0.0443005 | 0.0257263 |
| 51th | -1.063 | 0.0269293 | 0.0144724 | 0.0261679 | 0.023894 |
| 52th | -0.977 | 0.0135515 | 0.0322481 | 0.0110237 | 0.0282923 |
| 53th | -0.962 | 0.0093829 | 0.0322481 | 0.00692 | 0.0282923 |
| 54^th^ | -0.875 | 0.0251325 | 0.059516 | 0.0128122 | 0.0609292 |
| 55^th^ | -0.805 | 0.0251325 | 0.1058508 | 0.0128122 | 0.1143692 |
| 56^th^ | -0.735 | 0.045445 | 0.1058508 | 0.0220078 | 0.1143692 |
| 57^th^ | -0.724 | 0.0279324 | 0.1058508 | 0.0356603 | 0.1143692 |
| 58^th^ | -0.678 | 0.010125 | 0.1058508 | 0.0104966 | 0.1143692 |
| 59^th^ | -0.657 | 0.010125 | 0.1058508 | 0.0104966 | 0.1143692 |
| 60^th^ | -0.576 | 0.0238661 | 0.189464 | 0.023784 | 0.1730327 |
| 61th | -0.453 | 0.0511229 | 0.3481929 | 0.023784 | 0.2447171 |
| 62th | -0.25 | 0.0375871 | 0.4189668 | 0.0442883 | 0.4583101 |
| 63th | -0.181 | 0.0375871 | 0.4189668 | 0.0442883 | 0.4583101 |
| 64^th^ | -0.091 | 0.0423785 | 0.1862596 | 0.0371223 | 0.2139885 |
| 65^th^ | -0.005 | 0.0423785 | 0.1862596 | 0.0371223 | 0.2139885 |
| 66^th^ | 0.067 | 0.0717323 | 0.1862596 | 0.0631466 | 0.2139885 |
| 67^th^ | 0.359 | 0.0717323 | 0.3344927 | 0.0631466 | 0.7766817 |
| 68^th^ | 0.566 | 0.0551628 | 0.556003 | 0.0310038 | 0.8861943 |
| 69^th^ | 0.672 | 0.0340519 | 0.4155064 | 0.0155767 | 0.9021123 |
| 70^th^ | 0.917 | 0.0138912 | 0.4155064 | 0.0339882 | 0.9021123 |
| 71th | 1.071 | 0.0138912 | 0.4155064 | 0.0339882 | 0.9021123 |
| 72th | 1.31 | 0.0146626 | 0.437545 | 0.0356335 | 0.9969449 |
| 73th | 1.408 | 0.0146626 | 0.437545 | 0.0356335 | 0.9969449 |
| 74^th^ | 1.489 | 0.010792 | 0.9209131 | 0.0177827 | 0.6360552 |
| 75^th^ | 1.549 | 0.010792 | 0.9209131 | 0.0177827 | 0.6360552 |

Table S12: Evaluation of E2F score cutpoints in FFPE cohorts (predictive effects)

| rname | cutoff | OS.low.E2F | OS.High.E2F | OS.int.p | PFS.low.E2F | PFS.high.E2F | PFS.int.p |
| --- | --- | --- | --- | --- | --- | --- | --- |
| 25th | -2.496 | 0.91917 | 0.54492 | 0.96655 | 0.33975 | 0.14898 | 0.86068 |
| 26th | -2.477 | 0.91917 | 0.54492 | 0.96655 | 0.33975 | 0.14898 | 0.86068 |
| 27th | -2.392 | 0.91917 | 0.54492 | 0.96655 | 0.33975 | 0.14898 | 0.86068 |
| 28th | -2.304 | 0.91917 | 0.54492 | 0.96655 | 0.33975 | 0.14898 | 0.86068 |
| 29th | -2.226 | 0.51567 | 0.55472 | 0.65409 | 0.24603 | 0.14624 | 0.72479 |
| 30th | -2.199 | 0.35111 | 0.61384 | 0.4853 | 0.14763 | 0.16614 | 0.58609 |
| 31th | -2.172 | 0.35111 | 0.61384 | 0.4853 | 0.14763 | 0.16614 | 0.58609 |
| 32th | -2.111 | 0.35111 | 0.61384 | 0.4853 | 0.14763 | 0.16614 | 0.58609 |
| 33th | -2.081 | 0.35111 | 0.61384 | 0.4853 | 0.14763 | 0.16614 | 0.58609 |
| 34th | -2.009 | 0.35111 | 0.61384 | 0.4853 | 0.14763 | 0.16614 | 0.58609 |
| 35th | -1.933 | 0.35111 | 0.61384 | 0.4853 | 0.14763 | 0.16614 | 0.58609 |
| 36th | -1.86 | 0.35111 | 0.61384 | 0.4853 | 0.14763 | 0.16614 | 0.58609 |
| 37th | -1.83 | 0.47531 | 0.55914 | 0.49686 | 0.10929 | 0.18585 | 0.53244 |
| 38th | -1.822 | 0.47531 | 0.55914 | 0.49686 | 0.10929 | 0.18585 | 0.53244 |
| 39th | -1.812 | 0.47531 | 0.55914 | 0.49686 | 0.10929 | 0.18585 | 0.53244 |
| 40th | -1.793 | 0.47531 | 0.55914 | 0.49686 | 0.10929 | 0.18585 | 0.53244 |
| 41th | -1.744 | 0.59335 | 0.45451 | 0.65502 | 0.22694 | 0.12935 | 0.77495 |
| 42th | -1.634 | 0.70337 | 0.35506 | 0.81639 | 0.18504 | 0.13589 | 0.74384 |
| 43th | -1.594 | 0.70337 | 0.35506 | 0.81639 | 0.18504 | 0.13589 | 0.74384 |
| 44th | -1.576 | 0.70337 | 0.35506 | 0.81639 | 0.18504 | 0.13589 | 0.74384 |
| 45th | -1.563 | 0.70337 | 0.35506 | 0.81639 | 0.18504 | 0.13589 | 0.74384 |
| 46th | -1.45 | 0.80492 | 0.25363 | 0.97963 | 0.29699 | 0.08363 | 0.99468 |
| 47th | -1.388 | 0.89818 | 0.17569 | 0.82721 | 0.41446 | 0.04897 | 0.79157 |
| 48th | -1.314 | 0.86407 | 0.15639 | 0.57123 | 0.52875 | 0.04077 | 0.57301 |
| 49th | -1.276 | 0.86407 | 0.15639 | 0.57123 | 0.52875 | 0.04077 | 0.57301 |
| 50th | -1.142 | 0.63102 | 0.16223 | 0.40676 | 0.62319 | 0.04217 | 0.43531 |
| 51th | -1.063 | 0.72772 | 0.16073 | 0.4629 | 0.5207 | 0.04379 | 0.46859 |
| 52th | -0.977 | 0.89155 | 0.25572 | 0.78978 | 0.29184 | 0.07012 | 0.71209 |
| 53th | -0.962 | 0.89155 | 0.25572 | 0.78978 | 0.29184 | 0.07012 | 0.71209 |
| 54th | -0.875 | 0.91589 | 0.23447 | 0.54805 | 0.38542 | 0.0718 | 0.54042 |
| 55th | -0.805 | 0.81015 | 0.37416 | 0.85686 | 0.32383 | 0.09042 | 0.57887 |
| 56th | -0.735 | 0.81015 | 0.37416 | 0.85686 | 0.32383 | 0.09042 | 0.57887 |
| 57th | -0.724 | 0.81015 | 0.37416 | 0.85686 | 0.32383 | 0.09042 | 0.57887 |
| 58th | -0.678 | 0.81015 | 0.37416 | 0.85686 | 0.32383 | 0.09042 | 0.57887 |
| 59th | -0.657 | 0.81015 | 0.37416 | 0.85686 | 0.32383 | 0.09042 | 0.57887 |
| 60th | -0.576 | 0.64604 | 0.44593 | 0.97806 | 0.24663 | 0.1123 | 0.6918 |
| 61th | -0.453 | 0.79864 | 0.3947 | 0.77183 | 0.36041 | 0.08617 | 0.42893 |
| 62th | -0.25 | 0.39685 | 0.70697 | 0.72252 | 0.12775 | 0.2606 | 0.99305 |
| 63th | -0.181 | 0.39685 | 0.70697 | 0.72252 | 0.12775 | 0.2606 | 0.99305 |
| 64th | -0.091 | 0.6415 | 0.43862 | 0.78394 | 0.25137 | 0.13228 | 0.54595 |
| 65th | -0.005 | 0.6415 | 0.43862 | 0.78394 | 0.25137 | 0.13228 | 0.54595 |
| 66th | 0.067 | 0.6415 | 0.43862 | 0.78394 | 0.25137 | 0.13228 | 0.54595 |
| 67th | 0.359 | 0.55202 | 0.54596 | 0.86207 | 0.11638 | 0.27903 | 0.92813 |
| 68th | 0.566 | 0.39698 | 0.80739 | 0.79965 | 0.07127 | 0.43041 | 0.80471 |
| 69th | 0.672 | 0.36772 | 0.69335 | 0.95526 | 0.06828 | 0.40622 | 0.84304 |
| 70th | 0.917 | 0.49746 | 0.5207 | 0.77052 | 0.10668 | 0.2903 | 0.88569 |
| 71th | 1.071 | 0.64494 | 0.3419 | 0.50393 | 0.16142 | 0.1714 | 0.60214 |
| 72th | 1.31 | 0.57792 | 0.27987 | 0.47561 | 0.15664 | 0.13405 | 0.61066 |
| 73th | 1.408 | 0.66848 | 0.19906 | 0.3283 | 0.17047 | 0.10705 | 0.46856 |
| 74th | 1.489 | 0.53359 | 0.36846 | 0.62749 | 0.12877 | 0.24639 | 0.73904 |
| 75th | 1.549 | 0.53359 | 0.36846 | 0.62749 | 0.12877 | 0.24639 | 0.73904 |

## Analysis of prognostic effects for Non-ACT patients in each cohort (Table S13)

Table S13 Univariable analysis of prognostic effects

| Cohort | Stage | N1 | MST1 | N2 | MST2 | log-rank p | HR (95% CI) |
| --- | --- | --- | --- | --- | --- | --- | --- |
| MLOS |  |  |  |  |  |  |  |
|  | All (N=300) | 156 | NR | 144 | 40.4 | <0.001 | 2.58 (1.59-4.17) |
|  | Stage I (N=228) | 131 | NR | 97 | 53.5 | <0.001 | 2.73 (1.47-5.06) |
|  | Stage II (N=35) | 11 | NR | 24 | 19.4 | 0.165 | 2.80 (0.62-12.74) |
|  | Stage III/IV (N=32) | 12 | 31.6 | 20 | 35.3 | 0.736 | 0.84 (0.30-2.34) |
| MCLA |  |  |  |  |  |  |  |
|  | All (N=233) | 119 | NR | 114 | NR | <0.001 | 2.32 (1.48-3.64) |
|  | Stage I (N=164) | 96 | NR | 68 | NR | 0.027 | 2.04 (1.07-3.88) |
|  | Stage II (N=42) | 13 | 50.2 | 29 | 41.3 | 0.942 | 1.03 (0.44-2.41) |
|  | Stage III/IV (N=25) | 8 | 52 | 17 | 15.5 | 0.027 | 3.38 (1.09-10.52) |
| TCGA |  |  |  |  |  |  |  |
|  | All (N=436) | 185 | 53.3 | 251 | 35.8 | 0.008 | 1.74 (1.15-2.64) |
|  | Stage I (N=235) | 118 | NR | 117 | 48.5 | 0.522 | 1.26 (0.62-2.57) |
|  | Stage II (N=103) | 38 | 41.2 | 65 | 25.3 | 0.161 | 1.73 (0.80-3.76) |
|  | Stage III/IV (N=97) | 28 | 32.5 | 69 | 20.8 | 0.35 | 1.42 (0.68-2.98) |
| MLCom OS |  |  |  |  |  |  |  |
|  | All (N=101) | 55 | 75.5 | 46 | 42.6 | 0.041 | 1.87 (1.02-3.44) |
|  | Stage I (N=72) | 43 | NR | 29 | NR | 0.311 | 1.55 (0.66-3.66) |
|  | Stage II (N=13) | 5 | 64.7 | 8 | 12.4 | 0.059 | 4.54 (0.84-24.39) |
|  | Stage III/IV (N=16) | 7 | 45.7 | 9 | 33.7 | 0.523 | 1.47 (0.45-4.76) |
| NATCH OS |  |  |  |  |  |  |  |
|  | All (N=40) | 13 | 81.5 | 27 | 25.1 | 0.014 | 3.26 (1.21-8.78) |
|  | Stage I (N=22) | 9 | 81.5 | 13 | 48.5 | 0.079 | 3.79 (0.77-18.60) |
|  | Stage II (N=6) | 1 | NR | 5 | 20.7 | 0.259 | 786149084.39 (0.00-Inf) |
|  | Stage III/IV (N=12) | 3 | 22.8 | 9 | 17.6 | 0.804 | 1.20 (0.31-4.60) |
| MLCom PFS |  |  |  |  |  |  |  |
|  | All (N=98) | 54 | 59.8 | 44 | 17.3 | 0.044 | 1.75 (1.01-3.04) |
|  | Stage I (N=71) | 43 | 62.9 | 28 | 64.6 | 0.252 | 1.53 (0.73-3.19) |
|  | Stage II (N=12) | 5 | 44.5 | 7 | 7.3 | 0.066 | 3.64 (0.85-15.51) |
|  | Stage III/IV (N=15) | 6 | 43.3 | 9 | 16.4 | 0.723 | 1.24 (0.38-3.99) |
| NATCH PFS |  |  |  |  |  |  |  |
|  | All (N=40) | 13 | 30.9 | 27 | 7.6 | 0.026 | 2.57 (1.09-6.06) |
|  | Stage I (N=22) | 9 | NR | 13 | 17.9 | 0.131 | 2.43 (0.74-7.95) |
|  | Stage II (N=6) | 1 | NR | 5 | 9.8 | 0.259 | 786149084.39 (0.00-Inf) |
|  | Stage III/IV (N=12) | 3 | 9.2 | 9 | 5.5 | 0.335 | 1.93 (0.50-7.55) |
| MLCom+NATCH PFS |  |  |  |  |  |  |  |
|  | All (N=138) | 67 | 59.8 | 71 | 16.1 | <0.001 | 2.14 (1.36-3.36) |
|  | Stage I (N=93) | 52 | 62.9 | 41 | 26.1 | 0.046 | 1.84 (1.00-3.38) |
|  | Stage II (N=18) | 6 | 44.5 | 12 | 7.6 | 0.059 | 3.03 (0.91-10.03) |
|  | Stage III/IV (N=27) | 9 | 9.5 | 18 | 7.1 | 0.365 | 1.49 (0.63-3.53) |

## Multivariate analysis of prognostic effects (Table S14)

Table S14: Multivariate analysis of prognostic effects

| Cohort | Stage | variable | HR | 95% CI | wald p-value |
| --- | --- | --- | --- | --- | --- |
| MLOS | All | E2F score (High vs Low) | 2.22 | (1.35,3.65) | 0.002 |
| MLOS | All | Stage (II vs I) | 2.81 | (1.52,5.21) | 0.001 |
| MLOS | All | Stage (III/IV vs I) | 3.21 | (1.75,5.89) | <0.001 |
| MLOS | All | Gender (Male vs Female) | 2.34 | (1.44,3.78) | <0.001 |
| MLOS | Stage I | E2F score (High vs Low) | 2.90 | (1.56,5.39) | <0.001 |
| MLOS | Stage I | Gender (Male vs Female) | 2.09 | (1.13,3.84) | 0.018 |
| MLOS | Stage II | E2F score (High vs Low) | 2.81 | (0.62,12.78) | 0.18 |
| MLOS | Stage II | Gender (Male vs Female) | 0.82 | (0.27,2.45) | 0.72 |
| MLOS | Stage III/IV | E2F score (High vs Low) | 0.68 | (0.24,1.94) | 0.47 |
| MLOS | Stage III/IV | Gender (Male vs Female) | 8.58 | (2.31,31.90) | 0.001 |
| TCGA | All | E2F score (High vs Low) | 1.46 | (0.96,2.24) | 0.078 |
| TCGA | All | Stage (II vs I) | 2.21 | (1.33,3.67) | 0.002 |
| TCGA | All | Stage (III/IV vs I) | 3.42 | (2.13,5.49) | <0.001 |
| TCGA | All | Gender (Male vs Female) | 0.91 | (0.61,1.35) | 0.63 |
| TCGA | Stage I | E2F score (High vs Low) | 1.27 | (0.63,2.59) | 0.5 |
| TCGA | Stage I | Gender (Male vs Female) | 0.81 | (0.39,1.66) | 0.56 |
| TCGA | Stage II | E2F score (High vs Low) | 1.72 | (0.79,3.74) | 0.17 |
| TCGA | Stage II | Gender (Male vs Female) | 1.24 | (0.60,2.59) | 0.56 |
| TCGA | Stage III/IV | E2F score (High vs Low) | 1.41 | (0.67,2.97) | 0.36 |
| TCGA | Stage III/IV | Gender (Male vs Female) | 0.88 | (0.47,1.63) | 0.69 |
| MCLA | All | E2F score (High vs Low) | 1.95 | (1.22,3.12) | 0.005 |
| MCLA | All | Stage (II vs I) | 3.18 | (1.90,5.34) | <0.001 |
| MCLA | All | Stage (III/IV vs I) | 5.98 | (3.41,10.51) | <0.001 |
| MCLA | All | Gender (Male vs Female) | 1.18 | (0.76,1.82) | 0.46 |
| MCLA | Stage I | E2F score (High vs Low) | 2.03 | (1.07,3.87) | 0.031 |
| MCLA | Stage I | Gender (Male vs Female) | 0.92 | (0.48,1.75) | 0.8 |
| MCLA | Stage II | E2F score (High vs Low) | 1.30 | (0.50,3.38) | 0.59 |
| MCLA | Stage II | Gender (Male vs Female) | 1.54 | (0.62,3.79) | 0.35 |
| MCLA | Stage III/IV | E2F score (High vs Low) | 3.51 | (1.06,11.58) | 0.039 |
| MCLA | Stage III/IV | Gender (Male vs Female) | 0.91 | (0.35,2.38) | 0.84 |
| LCBRN | All | E2F score (High vs Low) | 5.95 | (1.25,28.18) | 0.025 |
| LCBRN | All | Stage (II vs I) | 3.04 | (0.82,11.30) | 0.098 |
| LCBRN | All | Stage (III/IV vs I) | 3.91 | (0.74,20.66) | 0.11 |
| LCBRN | All | Gender (Male vs Female) | 20.13 | (2.52,160.99) | 0.005 |
| LCBRN | Stage I | E2F score (High vs Low) | 6.71 | (0.74,60.85) | 0.091 |
| LCBRN | Stage I | Gender (Male vs Female) | 7.79 | (0.86,70.70) | 0.068 |
| LCBRN | Stage II | E2F score (High vs Low) | 2.33 | (0.24,22.78) | 0.47 |
| LCBRN | Stage II | Gender (Male vs Female) | 3147405306.88 | (0.00,Inf) | 1 |
| LCBRN | Stage III/IV | E2F score (High vs Low) | 1179938740.20 | (0.00,Inf) | 1 |
| LCBRN | Stage III/IV | Gender (Male vs Female) | 771814889.34 | (0.00,Inf) | 1 |
| JBR10 NSCLC OS | All | E2F score (High vs Low) | 1.95 | (0.87,4.38) | 0.1 |
| JBR10 NSCLC OS | All | Stage (II vs I) | 2.15 | (1.02,4.55) | 0.044 |
| JBR10 NSCLC OS | All | Gender (Male vs Female) | 2.13 | (0.80,5.67) | 0.13 |
| JBR10 NSCLC OS | Stage I | E2F score (High vs Low) | 3.83 | (1.04,14.16) | 0.044 |
| JBR10 NSCLC OS | Stage I | Gender (Male vs Female) | 0.61 | (0.17,2.24) | 0.46 |
| JBR10 NSCLC OS | Stage II | E2F score (High vs Low) | 1.46 | (0.51,4.13) | 0.48 |
| JBR10 NSCLC OS | Stage II | Gender (Male vs Female) | 7.82 | (1.03,59.54) | 0.047 |
| JBR10 NSCLC DSS | All | E2F score (High vs Low) | 1.89 | (0.80,4.48) | 0.15 |
| JBR10 NSCLC DSS | All | Stage (II vs I) | 2.25 | (1.00,5.05) | 0.049 |
| JBR10 NSCLC DSS | All | Gender (Male vs Female) | 1.75 | (0.65,4.74) | 0.27 |
| JBR10 NSCLC DSS | Stage I | E2F score (High vs Low) | 2.79 | (0.71,10.90) | 0.14 |
| JBR10 NSCLC DSS | Stage I | Gender (Male vs Female) | 0.52 | (0.13,2.01) | 0.34 |
| JBR10 NSCLC DSS | Stage II | E2F score (High vs Low) | 1.64 | (0.52,5.16) | 0.4 |
| JBR10 NSCLC DSS | Stage II | Gender (Male vs Female) | 6.85 | (0.89,52.65) | 0.065 |
| JBR10 AD OS | All | E2F score (High vs Low) | 3.13 | (1.07,9.18) | 0.038 |
| JBR10 AD OS | All | Stage (II vs I) | 2.80 | (0.98,8.04) | 0.055 |
| JBR10 AD OS | All | Gender (Male vs Female) | 1.99 | (0.62,6.38) | 0.24 |
| JBR10 AD OS | Stage I | E2F score (High vs Low) | 4.02 | (0.76,21.20) | 0.1 |
| JBR10 AD OS | Stage I | Gender (Male vs Female) | 0.81 | (0.15,4.29) | 0.81 |
| JBR10 AD OS | Stage II | E2F score (High vs Low) | 3.00 | (0.68,13.20) | 0.15 |
| JBR10 AD OS | Stage II | Gender (Male vs Female) | 4.71 | (0.58,38.49) | 0.15 |
| JBR10 AD DSS | All | E2F score (High vs Low) | 3.13 | (1.07,9.18) | 0.038 |
| JBR10 AD DSS | All | Stage (II vs I) | 2.80 | (0.98,8.04) | 0.055 |
| JBR10 AD DSS | All | Gender (Male vs Female) | 1.99 | (0.62,6.38) | 0.24 |
| JBR10 AD DSS | Stage I | E2F score (High vs Low) | 4.02 | (0.76,21.20) | 0.1 |
| JBR10 AD DSS | Stage I | Gender (Male vs Female) | 0.81 | (0.15,4.29) | 0.81 |
| JBR10 AD DSS | Stage II | E2F score (High vs Low) | 3.00 | (0.68,13.20) | 0.15 |
| JBR10 AD DSS | Stage II | Gender (Male vs Female) | 4.71 | (0.58,38.49) | 0.15 |
| MLOS+TCGA+MCLA+LCBRN+JBR10.AD | All | E2F score (High vs Low) | 1.88 | (1.45,2.43) | <0.001 |
| MLOS+TCGA+MCLA+LCBRN+JBR10.AD | All | Stage (II vs I) | 2.76 | (2.05,3.71) | <0.001 |
| MLOS+TCGA+MCLA+LCBRN+JBR10.AD | All | Stage (III/IV vs I) | 4.11 | (3.07,5.50) | <0.001 |
| MLOS+TCGA+MCLA+LCBRN+JBR10.AD | All | Gender (Male vs Female) | 1.38 | (1.09,1.76) | 0.008 |
| MLOS+TCGA+MCLA+LCBRN+JBR10.AD | Stage I | E2F score (High vs Low) | 2.16 | (1.49,3.12) | <0.001 |
| MLOS+TCGA+MCLA+LCBRN+JBR10.AD | Stage I | Gender (Male vs Female) | 1.29 | (0.90,1.85) | 0.17 |
| MLOS+TCGA+MCLA+LCBRN+JBR10.AD | Stage II | E2F score (High vs Low) | 1.59 | (0.94,2.67) | 0.081 |
| MLOS+TCGA+MCLA+LCBRN+JBR10.AD | Stage II | Gender (Male vs Female) | 1.49 | (0.93,2.39) | 0.095 |
| MLOS+TCGA+MCLA+LCBRN+JBR10.AD | Stage III/IV | E2F score (High vs Low) | 1.59 | (0.95,2.65) | 0.078 |
| MLOS+TCGA+MCLA+LCBRN+JBR10.AD | Stage III/IV | Gender (Male vs Female) | 1.42 | (0.91,2.22) | 0.12 |
| MLCom OS | All | E2F score (High vs Low) | 1.59 | (0.85,2.98) | 0.14 |
| MLCom OS | All | Stage (II vs I) | 3.27 | (1.48,7.21) | 0.003 |
| MLCom OS | All | Stage (III/IV vs I) | 3.85 | (1.87,7.93) | <0.001 |
| MLCom OS | All | Gender (Male vs Female) | 1.33 | (0.72,2.47) | 0.37 |
| MLCom OS | Stage I | E2F score (High vs Low) | 1.43 | (0.60,3.39) | 0.42 |
| MLCom OS | Stage I | Gender (Male vs Female) | 1.86 | (0.75,4.66) | 0.18 |
| MLCom OS | Stage II | E2F score (High vs Low) | 5.02 | (0.85,29.69) | 0.075 |
| MLCom OS | Stage II | Gender (Male vs Female) | 1.35 | (0.31,5.98) | 0.69 |
| MLCom OS | Stage III/IV | E2F score (High vs Low) | 1.67 | (0.42,6.68) | 0.47 |
| MLCom OS | Stage III/IV | Gender (Male vs Female) | 0.76 | (0.16,3.62) | 0.73 |
| MLCom PFS | All | E2F score (High vs Low) | 1.65 | (0.94,2.90) | 0.082 |
| MLCom PFS | All | Stage (II vs I) | 3.03 | (1.46,6.29) | 0.003 |
| MLCom PFS | All | Stage (III/IV vs I) | 2.43 | (1.21,4.87) | 0.013 |
| MLCom PFS | All | Gender (Male vs Female) | 0.74 | (0.42,1.31) | 0.3 |
| MLCom PFS | Stage I | E2F score (High vs Low) | 1.61 | (0.76,3.39) | 0.21 |
| MLCom PFS | Stage I | Gender (Male vs Female) | 0.73 | (0.35,1.54) | 0.41 |
| MLCom PFS | Stage II | E2F score (High vs Low) | 4.15 | (0.82,21.09) | 0.086 |
| MLCom PFS | Stage II | Gender (Male vs Female) | 1.32 | (0.30,5.82) | 0.71 |
| MLCom PFS | Stage III/IV | E2F score (High vs Low) | 1.62 | (0.38,7.02) | 0.52 |
| MLCom PFS | Stage III/IV | Gender (Male vs Female) | 0.60 | (0.11,3.20) | 0.55 |
| NATCH OS | All | E2F score (High vs Low) | 2.94 | (1.06,8.16) | 0.039 |
| NATCH OS | All | Stage (II vs I) | 2.08 | (0.60,7.16) | 0.25 |
| NATCH OS | All | Stage (III/IV vs I) | 6.71 | (2.44,18.43) | <0.001 |
| NATCH OS | All | Gender (Male vs Female) | 3.27 | (1.08,9.88) | 0.036 |
| NATCH OS | Stage I | E2F score (High vs Low) | 5.12 | (1.00,26.34) | 0.051 |
| NATCH OS | Stage I | Gender (Male vs Female) | 6.52 | (0.77,55.15) | 0.085 |
| NATCH OS | Stage II | E2F score (High vs Low) | 786149084.39 | (0.00,Inf) | 1 |
| NATCH OS | Stage II | Gender (Male vs Female) | NA | ( NA, NA) | NA |
| NATCH OS | Stage III/IV | E2F score (High vs Low) | 1.73 | (0.40,7.45) | 0.46 |
| NATCH OS | Stage III/IV | Gender (Male vs Female) | 2.57 | (0.49,13.60) | 0.27 |
| NATCH PFS | All | E2F score (High vs Low) | 2.59 | (1.08,6.24) | 0.034 |
| NATCH PFS | All | Stage (II vs I) | 1.26 | (0.40,3.97) | 0.69 |
| NATCH PFS | All | Stage (III/IV vs I) | 8.00 | (2.88,22.22) | <0.001 |
| NATCH PFS | All | Gender (Male vs Female) | 2.50 | (0.93,6.77) | 0.071 |
| NATCH PFS | Stage I | E2F score (High vs Low) | 2.49 | (0.76,8.21) | 0.13 |
| NATCH PFS | Stage I | Gender (Male vs Female) | 3.59 | (0.78,16.46) | 0.1 |
| NATCH PFS | Stage II | E2F score (High vs Low) | 786149084.39 | (0.00,Inf) | 1 |
| NATCH PFS | Stage II | Gender (Male vs Female) | NA | ( NA, NA) | NA |
| NATCH PFS | Stage III/IV | E2F score (High vs Low) | 2.81 | (0.59,13.41) | 0.19 |
| NATCH PFS | Stage III/IV | Gender (Male vs Female) | 2.46 | (0.40,15.09) | 0.33 |
| MLCom+NATCH OS | All | E2F score (High vs Low) | 1.80 | (1.08,3.00) | 0.025 |
| MLCom+NATCH OS | All | Stage (II vs I) | 2.90 | (1.49,5.62) | 0.002 |
| MLCom+NATCH OS | All | Stage (III/IV vs I) | 4.80 | (2.74,8.43) | <0.001 |
| MLCom+NATCH OS | All | Gender (Male vs Female) | 1.65 | (1.00,2.74) | 0.05 |
| MLCom+NATCH OS | Stage I | E2F score (High vs Low) | 1.94 | (0.94,4.02) | 0.074 |
| MLCom+NATCH OS | Stage I | Gender (Male vs Female) | 2.19 | (0.97,4.94) | 0.058 |
| MLCom+NATCH OS | Stage II | E2F score (High vs Low) | 3.40 | (0.88,13.07) | 0.075 |
| MLCom+NATCH OS | Stage II | Gender (Male vs Female) | 1.28 | (0.38,4.29) | 0.69 |
| MLCom+NATCH OS | Stage III/IV | E2F score (High vs Low) | 1.33 | (0.54,3.28) | 0.53 |
| MLCom+NATCH OS | Stage III/IV | Gender (Male vs Female) | 1.50 | (0.64,3.49) | 0.35 |
| MLCom+NATCH PFS | All | E2F score (High vs Low) | 1.81 | (1.14,2.88) | 0.012 |
| MLCom+NATCH PFS | All | Stage (II vs I) | 2.25 | (1.22,4.13) | 0.009 |
| MLCom+NATCH PFS | All | Stage (III/IV vs I) | 3.52 | (2.08,5.96) | <0.001 |
| MLCom+NATCH PFS | All | Gender (Male vs Female) | 1.24 | (0.78,1.95) | 0.36 |
| MLCom+NATCH PFS | Stage I | E2F score (High vs Low) | 1.82 | (0.99,3.36) | 0.054 |
| MLCom+NATCH PFS | Stage I | Gender (Male vs Female) | 1.11 | (0.60,2.06) | 0.74 |
| MLCom+NATCH PFS | Stage II | E2F score (High vs Low) | 2.97 | (0.89,9.90) | 0.077 |
| MLCom+NATCH PFS | Stage II | Gender (Male vs Female) | 1.19 | (0.39,3.62) | 0.76 |
| MLCom+NATCH PFS | Stage III/IV | E2F score (High vs Low) | 1.26 | (0.51,3.10) | 0.62 |
| MLCom+NATCH PFS | Stage III/IV | Gender (Male vs Female) | 1.69 | (0.71,4.03) | 0.24 |
| JBR10 NSCLC+NATCH OS | All | E2F score (High vs Low) | 2.25 | (1.21,4.19) | 0.01 |
| JBR10 NSCLC+NATCH OS | All | Stage (II vs I) | 2.04 | (1.11,3.76) | 0.022 |
| JBR10 NSCLC+NATCH OS | All | Stage (III/IV vs I) | 6.36 | (2.96,13.67) | <0.001 |
| JBR10 NSCLC+NATCH OS | All | Gender (Male vs Female) | 2.53 | (1.23,5.22) | 0.012 |
| JBR10 NSCLC+NATCH OS | Stage I | E2F score (High vs Low) | 3.51 | (1.34,9.21) | 0.011 |
| JBR10 NSCLC+NATCH OS | Stage I | Gender (Male vs Female) | 1.40 | (0.51,3.89) | 0.51 |
| JBR10 NSCLC+NATCH OS | Stage II | E2F score (High vs Low) | 1.58 | (0.57,4.34) | 0.38 |
| JBR10 NSCLC+NATCH OS | Stage II | Gender (Male vs Female) | 9.01 | (1.18,68.64) | 0.034 |
| JBR10 NSCLC+NATCH OS | Stage III/IV | E2F score (High vs Low) | 1.73 | (0.40,7.45) | 0.46 |
| JBR10 NSCLC+NATCH OS | Stage III/IV | Gender (Male vs Female) | 2.57 | (0.49,13.60) | 0.27 |
| JBR10 AD+NATCH OS | All | E2F score (High vs Low) | 2.87 | (1.41,5.82) | 0.004 |
| JBR10 AD+NATCH OS | All | Stage (II vs I) | 2.64 | (1.23,5.64) | 0.013 |
| JBR10 AD+NATCH OS | All | Stage (III/IV vs I) | 5.88 | (2.53,13.66) | <0.001 |
| JBR10 AD+NATCH OS | All | Gender (Male vs Female) | 2.69 | (1.22,5.95) | 0.014 |
| JBR10 AD+NATCH OS | Stage I | E2F score (High vs Low) | 4.29 | (1.42,12.94) | 0.01 |
| JBR10 AD+NATCH OS | Stage I | Gender (Male vs Female) | 2.17 | (0.67,6.99) | 0.19 |
| JBR10 AD+NATCH OS | Stage II | E2F score (High vs Low) | 2.78 | (0.72,10.63) | 0.14 |
| JBR10 AD+NATCH OS | Stage II | Gender (Male vs Female) | 5.63 | (0.69,46.12) | 0.11 |
| JBR10 AD+NATCH OS | Stage III/IV | E2F score (High vs Low) | 1.73 | (0.40,7.45) | 0.46 |
| JBR10 AD+NATCH OS | Stage III/IV | Gender (Male vs Female) | 2.57 | (0.49,13.60) | 0.27 |

## Evaluation of prognostic effect for the combined cohorts from the two randomized clinical trials (JBR10.NSCLC + NATCH or JBR10.AD + NATCH) (Table S15-S16)

The combined cohorts (JBR10.NSCLC+NATCH) from the two randomized clinical trials showed statistically significant association with OS (with or without covariate adjustment) in all stages (HR=2.25-2.99; p=0.01 to <0.001) and stage I (HR=3.51-3.69; p=0.011 to 0.007) for Non-ACT patients (Table S15-S16). The combined cohorts (JBR10.AD+NATCH) also had a similar significant result in all stage non-ACT patients (HR=2.87-3.75; p=0.004 to <0.001), as well as stage I (HR=4.29-4.41; p=0.01 to 0.008) in (Table S15-S16).

Table S15: Univariable analysis of prognostic effect for the combined clinical trial cohorts

| Cohort | Stage | N1 | MST1 | N2 | MST2 | log-rank p | HR (95% CI) |
| --- | --- | --- | --- | --- | --- | --- | --- |
| J+N |  |  |  |  |  |  |  |
|  | All (N=102) | 41 | NR | 61 | 29.2 | <0.001 | 2.99 (1.62-5.50) |
|  | Stage I (N=56) | 28 | NR | 28 | 48.5 | 0.004 | 3.69 (1.42-9.57) |
|  | Stage II (N=34) | 10 | 73.2 | 24 | 24.1 | 0.133 | 2.12 (0.78-5.79) |
|  | Stage III/IV (N=12) | 3 | 22.8 | 9 | 17.6 | 0.804 | 1.20 (0.31-4.60) |
| J.AD+N |  |  |  |  |  |  |  |
|  | All (N=72) | 34 | NR | 38 | 25.1 | <0.001 | 3.75 (1.89-7.46) |
|  | Stage I (N=42) | 24 | NR | 18 | 48.5 | 0.004 | 4.41 (1.48-13.13) |
|  | Stage II (N=18) | 7 | 73.2 | 11 | 18 | 0.049 | 3.50 (0.93-13.15) |
|  | Stage III/IV (N=12) | 3 | 22.8 | 9 | 17.6 | 0.804 | 1.20 (0.31-4.60) |

Table S16: Multivariable analysis of prognostic effect for the combined clinical trial cohorts

|  | variable | HR | 95% CI | wald p-value |
| --- | --- | --- | --- | --- |
| JBR10 NSCLC + NATCH All stages | E2F score (High vs Low) | 2.25 | (1.21,4.19) | 0.01 |
|  | Stage (II vs I) | 2.04 | (1.11,3.76) | 0.022 |
|  | Stage (III/IV vs I) | 6.36 | (2.96,13.67) | <0.001 |
|  | Gender (Male vs Female) | 2.53 | (1.23,5.22) | 0.012 |
| JBR10 NSCLC + NATCH Stage I | E2F score (High vs Low) | 3.51 | (1.34,9.21) | 0.011 |
|  | Gender (Male vs Female) | 1.40 | (0.51,3.89) | 0.51 |
| JBR10 NSCLC + NATCH Stage II | E2F score (High vs Low) | 1.58 | (0.57,4.34) | 0.38 |
|  | Gender (Male vs Female) | 9.01 | (1.18,68.64) | 0.034 |
| JBR10 AD + NATCH All stages | E2F score (High vs Low) | 2.87 | (1.41,5.82) | 0.004 |
|  | Stage (II vs I) | 2.64 | (1.23,5.64) | 0.013 |
|  | Stage (III/IV vs I) | 5.88 | (2.53,13.66) | <0.001 |
|  | Gender (Male vs Female) | 2.69 | (1.22,5.95) | 0.014 |
| JBR10 AD + NATCH Stage I | E2F score (High vs Low) | 4.29 | (1.42,12.94) | 0.01 |
|  | Gender (Male vs Female) | 2.17 | (0.67,6.99) | 0.19 |
| JBR10 AD + NATCH Stage II | E2F score (High vs Low) | 2.78 | (0.72,10.63) | 0.14 |
|  | Gender (Male vs Female) | 5.63 | (0.69,46.12) | 0.11 |

## Evaluation of predictive effect for PFS in the NATCH cohort and DSS in the JBR10 cohort (Table S17)

Table S17 summarized the predictive results. Specifically, analysis of PFS in the NATCH cohort yielded a non-significant interaction effect in all stage patients and each stage. However, the high E2F score group showed a significant positive ACT treatment effect with a better survival in ACT patients in all stage patients (HR=0.50; p=0.042) and stage II patients (HR=0.14; p=0.045).
Analysis of DSS in the JBR10 cohort (JBR10.NSCLC and JBR10.AD) showed a significant interaction effect (p=0.02-0.047), as well as a significant treatment in the high E2F (HR=0.28-0.48; p=0.044-0.047), in all stage patients. For stage II patients, the JBR10.NSCLC cohort showed a borderline significant level of interaction effect (p=0.051), but a significant treatment in the high E2F (HR=0.39; p=0.049). The JBR10.AD cohort had a similar trend, but did not reach the significant level (interaction effect: p=0.1; treatment in the high E2F: HR=0.29; p=0.142).

Table S17: Predictive effects for PFS in the NATCH cohort and DSS in the JBR10 cohort

| Cohort | Stage | Group | N1 | MST1 | N2 | MST2 | log-rank p | HR (95% CI) | int-p | test PH |
| --- | --- | --- | --- | --- | --- | --- | --- | --- | --- | --- |
| J DSS |  |  |  |  |  |  |  |  |  |  |
|  | All | Low (N=58) | 28 | NR | 30 | 93.8 | 0.371 | 1.51 (0.61-3.77) | 0.047 | 0.79 |
|  |  | High (N=75) | 34 | 43.2 | 41 | NR | 0.042 | 0.48 (0.24-0.99) |  | 0.61 |
|  | Stage I | Low (N=37) | 19 | NR | 18 | NR | 0.833 | 1.16 (0.29-4.64) | 0.563 | 0.97 |
|  |  | High (N=36) | 15 | NR | 21 | NR | 0.512 | 0.69 (0.22-2.13) |  | 0.25 |
|  | Stage II | Low (N=21) | 9 | 73.2 | 12 | 63.2 | 0.447 | 1.62 (0.46-5.66) | 0.051 | 0.85 |
|  |  | High (N=39) | 19 | 29.2 | 20 | NR | 0.042 | 0.39 (0.15-1.00) |  | 0.86 |
| J.AD DSS |  |  |  |  |  |  |  |  |  |  |
|  | All | Low (N=48) | 21 | NR | 27 | 93.8 | 0.383 | 1.52 (0.59-3.94) | 0.02 | 0.79 |
|  |  | High (N=23) | 11 | 36.4 | 12 | NR | 0.033 | 0.28 (0.08-0.97) |  | 0.56 |
|  | Stage I | Low (N=30) | 15 | NR | 15 | NR | 0.578 | 1.53 (0.34-6.82) | 0.221 | 0.94 |
|  |  | High (N=12) | 5 | 37.2 | 7 | NR | 0.29 | 0.39 (0.06-2.37) |  | 0.12 |
|  | Stage II | Low (N=18) | 6 | 53.5 | 12 | 63.2 | 0.919 | 1.07 (0.31-3.71) | 0.1 | 0.96 |
|  |  | High (N=11) | 6 | 13.6 | 5 | NR | 0.12 | 0.29 (0.05-1.52) |  | 0.7 |
| N PFS |  |  |  |  |  |  |  |  |  |  |
|  | All | Low (N=23) | 13 | 30.9 | 10 | 44.2 | 0.623 | 0.75 (0.24-2.37) | 0.435 | 0.059 |
|  |  | High (N=51) | 27 | 7.6 | 24 | 47.9 | 0.042 | 0.50 (0.26-0.99) |  | 0.48 |
|  | Stage I | Low (N=16) | 9 | NR | 7 | NR | 0.578 | 0.62 (0.11-3.40) | 0.946 | 0.57 |
|  |  | High (N=22) | 13 | 17.9 | 9 | 79.6 | 0.347 | 0.59 (0.20-1.78) |  | 0.21 |
|  | Stage II | Low (N=2) | 1 | NR | 1 | 36 | 0.317 | 1615474836.54 (0.00-Inf) | 0.999 |  |
|  |  | High (N=11) | 5 | 9.8 | 6 | NR | 0.045 | 0.14 (0.02-1.29) |  | 0.84 |
|  | Stage III/IV | Low (N=5) | 3 | 9.2 | 2 | 30.7 | 0.063 | 0.00 (0.00-Inf) | 0.877 | 1 |
|  |  | High (N=18) | 9 | 5.5 | 9 | 8.5 | 0.111 | 0.43 (0.15-1.25) |  | 0.28 |

## Predictive effect (OS) after adjustment for tissue type, FF and FFPE (Table S18)

Table S18: Predictive effect (OS) after adjustment for tissue type, FF and FFPE

| Cohort | Stage | Group | N1 | rate1 (%) | N2 | rate2 (%) | Wald_test p | HR (95% CI) | int-p |
| --- | --- | --- | --- | --- | --- | --- | --- | --- | --- |
| J+N |  |  |  |  |  |  |  |  |  |
|  | All | Low (N=81) | 41 | 71 | 40 | 65 | 0.257 | 1.50 (0.74-3.03) | 0.014 |
|  |  | High (N=126) | 61 | 31 | 65 | 66 | 0.012 | 0.54 (0.33-0.87) |  |
|  | Stage I | Low (N=53) | 28 | 82 | 25 | 80 | 0.344 | 1.67 (0.58-4.84) | 0.217 |
|  |  | High (N=58) | 28 | 43 | 30 | 66 | 0.469 | 0.76 (0.36-1.60) |  |
|  | Stage IA | Low (N=4) | 2 | 100 | 2 | 50 | 1 | 1615474836.54 (0.00-Inf) | 0.999 |
|  |  | High (N=4) | 1 | 67 | 3 | NE | 0.529 | 0.41 (0.03-6.62) |  |
|  | Stage IB | Low (N=49) | 26 | 81 | 23 | 82 | 0.499 | 1.46 (0.49-4.36) | 0.344 |
|  |  | High (N=54) | 27 | 45 | 27 | 66 | 0.522 | 0.77 (0.34-1.72) |  |
|  | Stage II | Low (N=23) | 10 | 60 | 13 | 41 | 0.343 | 1.72 (0.56-5.23) | 0.01 |
|  |  | High (N=50) | 24 | 29 | 26 | 76 | 0.006 | 0.30 (0.13-0.71) |  |
|  | Stage III/IV | Low (N=5) | 3 | NE | 2 | NE | 0.467 | 0.43 (0.04-4.20) | 0.74 |
|  |  | High (N=18) | 9 | 33 | 9 | NE | 0.301 | 0.57 (0.20-1.65) |  |
| J.AD+N |  |  |  |  |  |  |  |  |  |
|  | All | Low (N=71) | 34 | 70 | 37 | 63 | 0.198 | 1.62 (0.78-3.39) | 0.017 |
|  |  | High (N=74) | 38 | 20 | 36 | 60 | 0.034 | 0.52 (0.29-0.95) |  |
|  | Stage I | Low (N=46) | 24 | 83 | 22 | 77 | 0.215 | 2.04 (0.66-6.28) | 0.183 |
|  |  | High (N=34) | 18 | 31 | 16 | 61 | 0.536 | 0.74 (0.28-1.92) |  |
|  | Stage IA | Low (N=4) | 2 | 100 | 2 | 50 | 1 | 1615474836.54 (0.00-Inf) | 0.999 |
|  |  | High (N=4) | 1 | 67 | 3 | NE | 0.529 | 0.41 (0.03-6.62) |  |
|  | Stage IB | Low (N=42) | 22 | 82 | 20 | 80 | 0.331 | 1.77 (0.56-5.62) | 0.327 |
|  |  | High (N=30) | 17 | 32 | 13 | 60 | 0.616 | 0.75 (0.25-2.28) |  |
|  | Stage II | Low (N=20) | 7 | 57 | 13 | 41 | 0.536 | 1.46 (0.44-4.88) | 0.016 |
|  |  | High (N=22) | 11 | 18 | 11 | 81 | 0.022 | 0.21 (0.06-0.80) |  |
|  | Stage III/IV | Low (N=5) | 3 | NE | 2 | NE | 0.467 | 0.43 (0.04-4.20) | 0.74 |
|  |  | High (N=18) | 9 | 33 | 9 | NE | 0.301 | 0.57 (0.20-1.65) |  |

**N1, rate1: no ACT group**
**N2, rate2: ACT group**

## Multivariate analysis of predictive effect (Table S19)

Table S19: Multivariate analysis of predictive effect

| Cohort | Stage | variable | HR | 95% CI | wald p-value |
| --- | --- | --- | --- | --- | --- |
| JBR10 OS | All | E2F score High vs. Low | 2.16 | (0.97,4.82) | 0.059 |
| JBR10 OS | All | Adjuvant chemotherapy ACT vs. No ACT | 1.67 | (0.72,3.86) | 0.23 |
| JBR10 OS | All | Gender Male vs. Female | 1.56 | (0.84,2.90) | 0.16 |
| JBR10 OS | All | Stage II vs. I | 1.66 | (0.99,2.80) | 0.055 |
| JBR10 OS | All | E2F/ACT interaction | 0.27 | (0.09,0.79) | 0.017 |
| JBR10 OS | All (Low E2F) | Adjuvant chemotherapy (ACT vs No ACT) | 1.67 | (0.72,3.87) | 0.24 |
| JBR10 OS | All (Low E2F) | Gender (Male vs Female) | 2.19 | (0.89,5.37) | 0.088 |
| JBR10 OS | All (Low E2F) | Stage (II vs I) | 3.08 | (1.33,7.15) | 0.009 |
| JBR10 OS | All (High E2F) | Adjuvant chemotherapy (ACT vs No ACT) | 0.48 | (0.25,0.92) | 0.027 |
| JBR10 OS | All (High E2F) | Gender (Male vs Female) | 1.12 | (0.49,2.56) | 0.78 |
| JBR10 OS | All (High E2F) | Stage (II vs I) | 1.14 | (0.60,2.18) | 0.69 |
| JBR10 OS | I | E2F score High vs. Low | 3.31 | (0.96,11.43) | 0.058 |
| JBR10 OS | I | Adjuvant chemotherapy ACT vs. No ACT | 1.72 | (0.49,6.11) | 0.4 |
| JBR10 OS | I | Gender Male vs. Female | 1.08 | (0.45,2.61) | 0.86 |
| JBR10 OS | I | E2F/ACT interaction | 0.40 | (0.08,1.96) | 0.26 |
| JBR10 OS | I (Low E2F) | Adjuvant chemotherapy (ACT vs No ACT) | 1.79 | (0.50,6.36) | 0.37 |
| JBR10 OS | I (Low E2F) | Gender (Male vs Female) | 1.58 | (0.44,5.63) | 0.48 |
| JBR10 OS | I (High E2F) | Adjuvant chemotherapy (ACT vs No ACT) | 0.65 | (0.25,1.73) | 0.39 |
| JBR10 OS | I (High E2F) | Gender (Male vs Female) | 0.78 | (0.25,2.45) | 0.67 |
| JBR10 OS | II | E2F score High vs. Low | 1.48 | (0.52,4.19) | 0.46 |
| JBR10 OS | II | Adjuvant chemotherapy ACT vs. No ACT | 1.52 | (0.49,4.67) | 0.46 |
| JBR10 OS | II | Gender Male vs. Female | 2.27 | (0.91,5.64) | 0.079 |
| JBR10 OS | II | E2F/ACT interaction | 0.20 | (0.05,0.86) | 0.03 |
| JBR10 OS | II (Low E2F) | Adjuvant chemotherapy (ACT vs No ACT) | 1.49 | (0.48,4.66) | 0.49 |
| JBR10 OS | II (Low E2F) | Gender (Male vs Female) | 4.25 | (0.92,19.69) | 0.064 |
| JBR10 OS | II (High E2F) | Adjuvant chemotherapy (ACT vs No ACT) | 0.35 | (0.14,0.88) | 0.026 |
| JBR10 OS | II (High E2F) | Gender (Male vs Female) | 1.68 | (0.49,5.78) | 0.41 |
| JBR10 DSS | All | E2F score High vs. Low | 2.00 | (0.85,4.69) | 0.11 |
| JBR10 DSS | All | Adjuvant chemotherapy ACT vs. No ACT | 1.44 | (0.58,3.60) | 0.43 |
| JBR10 DSS | All | Gender Male vs. Female | 1.47 | (0.75,2.89) | 0.26 |
| JBR10 DSS | All | Stage II vs. I | 2.05 | (1.15,3.65) | 0.015 |
| JBR10 DSS | All | E2F/ACT interaction | 0.31 | (0.10,0.99) | 0.047 |
| JBR10 DSS | All (Low E2F) | Adjuvant chemotherapy (ACT vs No ACT) | 1.46 | (0.58,3.67) | 0.42 |
| JBR10 DSS | All (Low E2F) | Gender (Male vs Female) | 2.13 | (0.80,5.69) | 0.13 |
| JBR10 DSS | All (Low E2F) | Stage (II vs I) | 3.39 | (1.34,8.59) | 0.01 |
| JBR10 DSS | All (High E2F) | Adjuvant chemotherapy (ACT vs No ACT) | 0.48 | (0.24,0.99) | 0.048 |
| JBR10 DSS | All (High E2F) | Gender (Male vs Female) | 1.10 | (0.45,2.69) | 0.83 |
| JBR10 DSS | All (High E2F) | Stage (II vs I) | 1.55 | (0.75,3.20) | 0.23 |
| JBR10 DSS | I | E2F score High vs. Low | 2.26 | (0.61,8.35) | 0.22 |
| JBR10 DSS | I | Adjuvant chemotherapy ACT vs. No ACT | 1.13 | (0.28,4.54) | 0.86 |
| JBR10 DSS | I | Gender Male vs. Female | 1.08 | (0.39,2.98) | 0.88 |
| JBR10 DSS | I | E2F/ACT interaction | 0.59 | (0.10,3.54) | 0.56 |
| JBR10 DSS | I (Low E2F) | Adjuvant chemotherapy (ACT vs No ACT) | 1.20 | (0.30,4.81) | 0.8 |
| JBR10 DSS | I (Low E2F) | Gender (Male vs Female) | 1.70 | (0.40,7.16) | 0.47 |
| JBR10 DSS | I (High E2F) | Adjuvant chemotherapy (ACT vs No ACT) | 0.67 | (0.21,2.09) | 0.49 |
| JBR10 DSS | I (High E2F) | Gender (Male vs Female) | 0.73 | (0.20,2.71) | 0.63 |
| JBR10 DSS | II | E2F score High vs. Low | 1.73 | (0.55,5.41) | 0.35 |
| JBR10 DSS | II | Adjuvant chemotherapy ACT vs. No ACT | 1.65 | (0.48,5.65) | 0.43 |
| JBR10 DSS | II | Gender Male vs. Female | 1.96 | (0.78,4.94) | 0.16 |
| JBR10 DSS | II | E2F/ACT interaction | 0.20 | (0.04,0.93) | 0.04 |
| JBR10 DSS | II (Low E2F) | Adjuvant chemotherapy (ACT vs No ACT) | 1.61 | (0.46,5.64) | 0.46 |
| JBR10 DSS | II (Low E2F) | Gender (Male vs Female) | 3.58 | (0.75,17.12) | 0.11 |
| JBR10 DSS | II (High E2F) | Adjuvant chemotherapy (ACT vs No ACT) | 0.38 | (0.15,0.97) | 0.044 |
| JBR10 DSS | II (High E2F) | Gender (Male vs Female) | 1.55 | (0.45,5.39) | 0.49 |
| JBR10.AD OS | All | E2F score High vs. Low | 3.84 | (1.33,11.08) | 0.013 |
| JBR10.AD OS | All | Adjuvant chemotherapy ACT vs. No ACT | 1.69 | (0.68,4.23) | 0.26 |
| JBR10.AD OS | All | Gender Male vs. Female | 1.84 | (0.91,3.74) | 0.091 |
| JBR10.AD OS | All | Stage II vs. I | 1.94 | (0.98,3.83) | 0.058 |
| JBR10.AD OS | All | E2F/ACT interaction | 0.21 | (0.05,0.89) | 0.034 |
| JBR10.AD OS | All (Low E2F) | Adjuvant chemotherapy (ACT vs No ACT) | 1.66 | (0.66,4.16) | 0.28 |
| JBR10.AD OS | All (Low E2F) | Gender (Male vs Female) | 2.04 | (0.81,5.12) | 0.13 |
| JBR10.AD OS | All (Low E2F) | Stage (II vs I) | 2.77 | (1.14,6.73) | 0.024 |
| JBR10.AD OS | All (High E2F) | Adjuvant chemotherapy (ACT vs No ACT) | 0.34 | (0.10,1.16) | 0.086 |
| JBR10.AD OS | All (High E2F) | Gender (Male vs Female) | 1.79 | (0.57,5.66) | 0.32 |
| JBR10.AD OS | All (High E2F) | Stage (II vs I) | 1.02 | (0.35,2.99) | 0.97 |
| JBR10.AD OS | I | E2F score High vs. Low | 5.25 | (0.98,28.17) | 0.053 |
| JBR10.AD OS | I | Adjuvant chemotherapy ACT vs. No ACT | 2.12 | (0.53,8.53) | 0.29 |
| JBR10.AD OS | I | Gender Male vs. Female | 1.70 | (0.60,4.79) | 0.32 |
| JBR10.AD OS | I | E2F/ACT interaction | 0.30 | (0.03,2.54) | 0.27 |
| JBR10.AD OS | I (Low E2F) | Adjuvant chemotherapy (ACT vs No ACT) | 2.17 | (0.54,8.71) | 0.28 |
| JBR10.AD OS | I (Low E2F) | Gender (Male vs Female) | 1.57 | (0.42,5.92) | 0.5 |
| JBR10.AD OS | I (High E2F) | Adjuvant chemotherapy (ACT vs No ACT) | 0.51 | (0.08,3.35) | 0.49 |
| JBR10.AD OS | I (High E2F) | Gender (Male vs Female) | 2.23 | (0.45,11.19) | 0.33 |
| JBR10.AD OS | II | E2F score High vs. Low | 2.71 | (0.70,10.57) | 0.15 |
| JBR10.AD OS | II | Adjuvant chemotherapy ACT vs. No ACT | 1.27 | (0.38,4.26) | 0.7 |
| JBR10.AD OS | II | Gender Male vs. Female | 2.49 | (0.85,7.31) | 0.097 |
| JBR10.AD OS | II | E2F/ACT interaction | 0.13 | (0.02,1.12) | 0.064 |
| JBR10.AD OS | II (Low E2F) | Adjuvant chemotherapy (ACT vs No ACT) | 1.29 | (0.38,4.37) | 0.68 |
| JBR10.AD OS | II (Low E2F) | Gender (Male vs Female) | 3.44 | (0.73,16.13) | 0.12 |
| JBR10.AD OS | II (High E2F) | Adjuvant chemotherapy (ACT vs No ACT) | 0.29 | (0.05,1.63) | 0.16 |
| JBR10.AD OS | II (High E2F) | Gender (Male vs Female) | 1.70 | (0.31,9.43) | 0.54 |
| JBR10.AD DSS | All | E2F score High vs. Low | 3.59 | (1.25,10.33) | 0.018 |
| JBR10.AD DSS | All | Adjuvant chemotherapy ACT vs. No ACT | 1.26 | (0.49,3.29) | 0.63 |
| JBR10.AD DSS | All | Gender Male vs. Female | 1.87 | (0.87,4.04) | 0.11 |
| JBR10.AD DSS | All | Stage II vs. I | 2.52 | (1.19,5.32) | 0.015 |
| JBR10.AD DSS | All | E2F/ACT interaction | 0.19 | (0.04,0.93) | 0.04 |
| JBR10.AD DSS | All (Low E2F) | Adjuvant chemotherapy (ACT vs No ACT) | 1.29 | (0.49,3.37) | 0.6 |
| JBR10.AD DSS | All (Low E2F) | Gender (Male vs Female) | 2.08 | (0.77,5.66) | 0.15 |
| JBR10.AD DSS | All (Low E2F) | Stage (II vs I) | 3.45 | (1.30,9.10) | 0.013 |
| JBR10.AD DSS | All (High E2F) | Adjuvant chemotherapy (ACT vs No ACT) | 0.32 | (0.09,1.15) | 0.08 |
| JBR10.AD DSS | All (High E2F) | Gender (Male vs Female) | 1.73 | (0.49,6.06) | 0.39 |
| JBR10.AD DSS | All (High E2F) | Stage (II vs I) | 1.44 | (0.45,4.61) | 0.54 |
| JBR10.AD DSS | I | E2F score High vs. Low | 4.26 | (0.80,22.67) | 0.089 |
| JBR10.AD DSS | I | Adjuvant chemotherapy ACT vs. No ACT | 1.40 | (0.31,6.27) | 0.66 |
| JBR10.AD DSS | I | Gender Male vs. Female | 1.62 | (0.49,5.40) | 0.43 |
| JBR10.AD DSS | I | E2F/ACT interaction | 0.29 | (0.03,3.28) | 0.32 |
| JBR10.AD DSS | I (Low E2F) | Adjuvant chemotherapy (ACT vs No ACT) | 1.45 | (0.32,6.52) | 0.63 |
| JBR10.AD DSS | I (Low E2F) | Gender (Male vs Female) | 1.71 | (0.38,7.77) | 0.49 |
| JBR10.AD DSS | I (High E2F) | Adjuvant chemotherapy (ACT vs No ACT) | 0.48 | (0.07,3.25) | 0.45 |
| JBR10.AD DSS | I (High E2F) | Gender (Male vs Female) | 1.81 | (0.27,12.31) | 0.54 |
| JBR10.AD DSS | II | E2F score High vs. Low | 2.88 | (0.74,11.26) | 0.13 |
| JBR10.AD DSS | II | Adjuvant chemotherapy ACT vs. No ACT | 1.12 | (0.32,3.87) | 0.86 |
| JBR10.AD DSS | II | Gender Male vs. Female | 2.39 | (0.80,7.12) | 0.12 |
| JBR10.AD DSS | II | E2F/ACT interaction | 0.14 | (0.02,1.21) | 0.074 |
| JBR10.AD DSS | II (Low E2F) | Adjuvant chemotherapy (ACT vs No ACT) | 1.15 | (0.33,4.01) | 0.83 |
| JBR10.AD DSS | II (Low E2F) | Gender (Male vs Female) | 3.14 | (0.66,14.98) | 0.15 |
| JBR10.AD DSS | II (High E2F) | Adjuvant chemotherapy (ACT vs No ACT) | 0.29 | (0.05,1.63) | 0.16 |
| JBR10.AD DSS | II (High E2F) | Gender (Male vs Female) | 1.70 | (0.31,9.43) | 0.54 |
| NATCH OS | All | E2F score High vs. Low | 3.03 | (1.11,8.25) | 0.03 |
| NATCH OS | All | Adjuvant chemotherapy ACT vs. No ACT | 0.97 | (0.26,3.63) | 0.96 |
| NATCH OS | All | Gender Male vs. Female | 2.15 | (1.01,4.58) | 0.048 |
| NATCH OS | All | Stage II vs. I | 1.30 | (0.49,3.43) | 0.6 |
| NATCH OS | All | Stage III/IV vs. I | 4.59 | (2.24,9.38) | <0.001 |
| NATCH OS | All | E2F/ACT interaction | 0.53 | (0.12,2.38) | 0.41 |
| NATCH OS | All (Low E2F) | Adjuvant chemotherapy (ACT vs No ACT) | 0.89 | (0.19,4.09) | 0.88 |
| NATCH OS | All (Low E2F) | Gender (Male vs Female) | 1.81 | (0.17,19.12) | 0.62 |
| NATCH OS | All (Low E2F) | Stage (II vs I) | 2.53 | (0.24,26.87) | 0.44 |
| NATCH OS | All (Low E2F) | Stage (III/IV vs I) | 12.03 | (2.07,69.88) | 0.006 |
| NATCH OS | All (High E2F) | Adjuvant chemotherapy (ACT vs No ACT) | 0.56 | (0.27,1.15) | 0.11 |
| NATCH OS | All (High E2F) | Gender (Male vs Female) | 1.91 | (0.84,4.36) | 0.12 |
| NATCH OS | All (High E2F) | Stage (II vs I) | 1.05 | (0.36,3.06) | 0.92 |
| NATCH OS | All (High E2F) | Stage (III/IV vs I) | 3.56 | (1.60,7.92) | 0.002 |
| NATCH OS | I | E2F score High vs. Low | 4.19 | (0.85,20.67) | 0.079 |
| NATCH OS | I | Adjuvant chemotherapy ACT vs. No ACT | 1.79 | (0.25,12.87) | 0.56 |
| NATCH OS | I | Gender Male vs. Female | 2.01 | (0.54,7.52) | 0.3 |
| NATCH OS | I | E2F/ACT interaction | 0.44 | (0.04,4.43) | 0.48 |
| NATCH OS | I (Low E2F) | Adjuvant chemotherapy (ACT vs No ACT) | 3.67 | (0.33,40.74) | 0.29 |
| NATCH OS | I (Low E2F) | Gender (Male vs Female) | 0.99 | (0.09,11.01) | 0.99 |
| NATCH OS | I (High E2F) | Adjuvant chemotherapy (ACT vs No ACT) | 0.77 | (0.23,2.56) | 0.67 |
| NATCH OS | I (High E2F) | Gender (Male vs Female) | 2.23 | (0.45,11.06) | 0.33 |
| NATCH OS | II | E2F score High vs. Low | 6.49 | (0.00,Inf) | 1 |
| NATCH OS | II | Adjuvant chemotherapy ACT vs. No ACT | 3.26 | (0.00,Inf) | 1 |
| NATCH OS | II | Gender Male vs. Female | 143403262.97 | (0.00,Inf) | 1 |
| NATCH OS | II | E2F/ACT interaction | 0.05 | (0.00,Inf) | 1 |
| NATCH OS | II (Low E2F) | Adjuvant chemotherapy (ACT vs No ACT) | 1615474836.54 | (0.00,Inf) | 1 |
| NATCH OS | II (Low E2F) | Gender (Male vs Female) | 1.00 | (1.00,1.00) | NA |
| NATCH OS | II (High E2F) | Adjuvant chemotherapy (ACT vs No ACT) | 0.14 | (0.01,1.31) | 0.084 |
| NATCH OS | II (High E2F) | Gender (Male vs Female) | 113378622.66 | (0.00,Inf) | 1 |
| NATCH OS | III/IV | E2F score High vs. Low | 1.63 | (0.41,6.41) | 0.49 |
| NATCH OS | III/IV | Adjuvant chemotherapy ACT vs. No ACT | 0.35 | (0.04,3.43) | 0.37 |
| NATCH OS | III/IV | Gender Male vs. Female | 1.77 | (0.60,5.21) | 0.3 |
| NATCH OS | III/IV | E2F/ACT interaction | 1.69 | (0.14,20.66) | 0.68 |
| NATCH OS | III/IV (Low E2F) | Adjuvant chemotherapy (ACT vs No ACT) | 0.43 | (0.04,4.20) | 0.47 |
| NATCH OS | III/IV (Low E2F) | Gender (Male vs Female) | NA | ( NA, NA) | NA |
| NATCH OS | III/IV (High E2F) | Adjuvant chemotherapy (ACT vs No ACT) | 0.61 | (0.21,1.81) | 0.37 |
| NATCH OS | III/IV (High E2F) | Gender (Male vs Female) | 1.72 | (0.58,5.07) | 0.33 |
| NATCH PFS | All | E2F score High vs. Low | 2.59 | (1.09,6.16) | 0.031 |
| NATCH PFS | All | Adjuvant chemotherapy ACT vs. No ACT | 0.54 | (0.16,1.74) | 0.3 |
| NATCH PFS | All | Gender Male vs. Female | 2.00 | (0.98,4.06) | 0.056 |
| NATCH PFS | All | Stage II vs. I | 0.99 | (0.39,2.52) | 0.99 |
| NATCH PFS | All | Stage III/IV vs. I | 5.65 | (2.84,11.23) | <0.001 |
| NATCH PFS | All | E2F/ACT interaction | 0.60 | (0.16,2.32) | 0.46 |
| NATCH PFS | All (Low E2F) | Adjuvant chemotherapy (ACT vs No ACT) | 0.37 | (0.10,1.45) | 0.16 |
| NATCH PFS | All (Low E2F) | Gender (Male vs Female) | 2.00 | (0.35,11.54) | 0.44 |
| NATCH PFS | All (Low E2F) | Stage (II vs I) | 1.40 | (0.15,13.11) | 0.77 |
| NATCH PFS | All (Low E2F) | Stage (III/IV vs I) | 7.84 | (1.83,33.66) | 0.006 |
| NATCH PFS | All (High E2F) | Adjuvant chemotherapy (ACT vs No ACT) | 0.38 | (0.19,0.79) | 0.009 |
| NATCH PFS | All (High E2F) | Gender (Male vs Female) | 1.99 | (0.88,4.47) | 0.096 |
| NATCH PFS | All (High E2F) | Stage (II vs I) | 0.92 | (0.32,2.59) | 0.87 |
| NATCH PFS | All (High E2F) | Stage (III/IV vs I) | 4.87 | (2.19,10.83) | <0.001 |
| NATCH PFS | I | E2F score High vs. Low | 2.43 | (0.74,7.98) | 0.14 |
| NATCH PFS | I | Adjuvant chemotherapy ACT vs. No ACT | 0.59 | (0.11,3.21) | 0.54 |
| NATCH PFS | I | Gender Male vs. Female | 2.04 | (0.66,6.31) | 0.21 |
| NATCH PFS | I | E2F/ACT interaction | 0.81 | (0.11,6.19) | 0.84 |
| NATCH PFS | I (Low E2F) | Adjuvant chemotherapy (ACT vs No ACT) | 0.60 | (0.11,3.34) | 0.56 |
| NATCH PFS | I (Low E2F) | Gender (Male vs Female) | 1.29 | (0.23,7.18) | 0.77 |
| NATCH PFS | I (High E2F) | Adjuvant chemotherapy (ACT vs No ACT) | 0.47 | (0.15,1.47) | 0.19 |
| NATCH PFS | I (High E2F) | Gender (Male vs Female) | 3.00 | (0.64,14.07) | 0.16 |
| NATCH PFS | II | E2F score High vs. Low | 5.45 | (0.00,Inf) | 1 |
| NATCH PFS | II | Adjuvant chemotherapy ACT vs. No ACT | 3.32 | (0.00,Inf) | 1 |
| NATCH PFS | II | Gender Male vs. Female | 134534150.71 | (0.00,Inf) | 1 |
| NATCH PFS | II | E2F/ACT interaction | 0.06 | (0.00,Inf) | 1 |
| NATCH PFS | II (Low E2F) | Adjuvant chemotherapy (ACT vs No ACT) | 1615474836.54 | (0.00,Inf) | 1 |
| NATCH PFS | II (Low E2F) | Gender (Male vs Female) | 1.00 | (1.00,1.00) | NA |
| NATCH PFS | II (High E2F) | Adjuvant chemotherapy (ACT vs No ACT) | 0.17 | (0.02,1.58) | 0.12 |
| NATCH PFS | II (High E2F) | Gender (Male vs Female) | 106870470.97 | (0.00,Inf) | 1 |
| NATCH PFS | III/IV | E2F score High vs. Low | 2.31 | (0.57,9.37) | 0.24 |
| NATCH PFS | III/IV | Adjuvant chemotherapy ACT vs. No ACT | 0.32 | (0.05,2.19) | 0.24 |
| NATCH PFS | III/IV | Gender Male vs. Female | 1.97 | (0.65,5.94) | 0.23 |
| NATCH PFS | III/IV | E2F/ACT interaction | 0.97 | (0.12,7.83) | 0.98 |
| NATCH PFS | III/IV (Low E2F) | Adjuvant chemotherapy (ACT vs No ACT) | 0.00 | (0.00,Inf) | 1 |
| NATCH PFS | III/IV (Low E2F) | Gender (Male vs Female) | 1.00 | (1.00,1.00) | NA |
| NATCH PFS | III/IV (High E2F) | Adjuvant chemotherapy (ACT vs No ACT) | 0.44 | (0.15,1.31) | 0.14 |
| NATCH PFS | III/IV (High E2F) | Gender (Male vs Female) | 1.56 | (0.53,4.56) | 0.41 |
| JBR10+NATCH OS | All | E2F score High vs. Low | 2.41 | (1.30,4.47) | 0.005 |
| JBR10+NATCH OS | All | Adjuvant chemotherapy ACT vs. No ACT | 1.42 | (0.71,2.87) | 0.32 |
| JBR10+NATCH OS | All | Gender Male vs. Female | 1.61 | (1.01,2.56) | 0.045 |
| JBR10+NATCH OS | All | Stage II vs. I | 1.52 | (0.98,2.37) | 0.063 |
| JBR10+NATCH OS | All | Stage III/IV vs. I | 4.67 | (2.68,8.15) | <0.001 |
| JBR10+NATCH OS | All | E2F/ACT interaction | 0.34 | (0.14,0.79) | 0.012 |
| JBR10+NATCH OS | All (Low E2F) | Adjuvant chemotherapy (ACT vs No ACT) | 1.35 | (0.67,2.74) | 0.4 |
| JBR10+NATCH OS | All (Low E2F) | Gender (Male vs Female) | 2.23 | (0.97,5.12) | 0.059 |
| JBR10+NATCH OS | All (Low E2F) | Stage (II vs I) | 3.19 | (1.50,6.79) | 0.003 |
| JBR10+NATCH OS | All (Low E2F) | Stage (III/IV vs I) | 6.14 | (1.87,20.09) | 0.003 |
| JBR10+NATCH OS | All (High E2F) | Adjuvant chemotherapy (ACT vs No ACT) | 0.51 | (0.32,0.83) | 0.007 |
| JBR10+NATCH OS | All (High E2F) | Gender (Male vs Female) | 1.44 | (0.81,2.59) | 0.22 |
| JBR10+NATCH OS | All (High E2F) | Stage (II vs I) | 1.09 | (0.64,1.87) | 0.75 |
| JBR10+NATCH OS | All (High E2F) | Stage (III/IV vs I) | 3.75 | (1.97,7.16) | <0.001 |
| JBR10+NATCH OS | I | E2F score High vs. Low | 3.53 | (1.36,9.18) | 0.01 |
| JBR10+NATCH OS | I | Adjuvant chemotherapy ACT vs. No ACT | 1.69 | (0.58,4.86) | 0.33 |
| JBR10+NATCH OS | I | Gender Male vs. Female | 1.30 | (0.64,2.62) | 0.47 |
| JBR10+NATCH OS | I | E2F/ACT interaction | 0.42 | (0.12,1.53) | 0.19 |
| JBR10+NATCH OS | I (Low E2F) | Adjuvant chemotherapy (ACT vs No ACT) | 1.72 | (0.59,4.97) | 0.32 |
| JBR10+NATCH OS | I (Low E2F) | Gender (Male vs Female) | 1.49 | (0.50,4.48) | 0.47 |
| JBR10+NATCH OS | I (High E2F) | Adjuvant chemotherapy (ACT vs No ACT) | 0.72 | (0.34,1.49) | 0.37 |
| JBR10+NATCH OS | I (High E2F) | Gender (Male vs Female) | 1.17 | (0.48,2.89) | 0.73 |
| JBR10+NATCH OS | II | E2F score High vs. Low | 1.68 | (0.61,4.63) | 0.32 |
| JBR10+NATCH OS | II | Adjuvant chemotherapy ACT vs. No ACT | 1.61 | (0.54,4.85) | 0.39 |
| JBR10+NATCH OS | II | Gender Male vs. Female | 2.53 | (1.03,6.22) | 0.043 |
| JBR10+NATCH OS | II | E2F/ACT interaction | 0.16 | (0.04,0.66) | 0.011 |
| JBR10+NATCH OS | II (Low E2F) | Adjuvant chemotherapy (ACT vs No ACT) | 1.50 | (0.49,4.61) | 0.48 |
| JBR10+NATCH OS | II (Low E2F) | Gender (Male vs Female) | 4.89 | (1.06,22.56) | 0.042 |
| JBR10+NATCH OS | II (High E2F) | Adjuvant chemotherapy (ACT vs No ACT) | 0.30 | (0.13,0.70) | 0.005 |
| JBR10+NATCH OS | II (High E2F) | Gender (Male vs Female) | 1.88 | (0.56,6.32) | 0.31 |
| JBR10+NATCH OS | III/IV | E2F score High vs. Low | 1.63 | (0.41,6.41) | 0.49 |
| JBR10+NATCH OS | III/IV | Adjuvant chemotherapy ACT vs. No ACT | 0.35 | (0.04,3.43) | 0.37 |
| JBR10+NATCH OS | III/IV | Gender Male vs. Female | 1.77 | (0.60,5.21) | 0.3 |
| JBR10+NATCH OS | III/IV | E2F/ACT interaction | 1.69 | (0.14,20.66) | 0.68 |
| JBR10+NATCH OS | III/IV (Low E2F) | Adjuvant chemotherapy (ACT vs No ACT) | 0.43 | (0.04,4.20) | 0.47 |
| JBR10+NATCH OS | III/IV (Low E2F) | Gender (Male vs Female) | NA | ( NA, NA) | NA |
| JBR10+NATCH OS | III/IV (High E2F) | Adjuvant chemotherapy (ACT vs No ACT) | 0.61 | (0.21,1.81) | 0.37 |
| JBR10+NATCH OS | III/IV (High E2F) | Gender (Male vs Female) | 1.72 | (0.58,5.07) | 0.33 |
| JBR10.AD+NATCH OS | All | E2F score High vs. Low | 3.07 | (1.53,6.17) | 0.002 |
| JBR10.AD+NATCH OS | All | Adjuvant chemotherapy ACT vs. No ACT | 1.45 | (0.69,3.02) | 0.32 |
| JBR10.AD+NATCH OS | All | Gender Male vs. Female | 1.86 | (1.12,3.09) | 0.016 |
| JBR10.AD+NATCH OS | All | Stage II vs. I | 1.82 | (1.07,3.10) | 0.028 |
| JBR10.AD+NATCH OS | All | Stage III/IV vs. I | 4.30 | (2.36,7.84) | <0.001 |
| JBR10.AD+NATCH OS | All | E2F/ACT interaction | 0.33 | (0.13,0.84) | 0.021 |
| JBR10.AD+NATCH OS | All (Low E2F) | Adjuvant chemotherapy (ACT vs No ACT) | 1.37 | (0.65,2.88) | 0.41 |
| JBR10.AD+NATCH OS | All (Low E2F) | Gender (Male vs Female) | 2.07 | (0.89,4.82) | 0.092 |
| JBR10.AD+NATCH OS | All (Low E2F) | Stage (II vs I) | 2.98 | (1.36,6.54) | 0.007 |
| JBR10.AD+NATCH OS | All (Low E2F) | Stage (III/IV vs I) | 5.84 | (1.75,19.53) | 0.004 |
| JBR10.AD+NATCH OS | All (High E2F) | Adjuvant chemotherapy (ACT vs No ACT) | 0.51 | (0.28,0.93) | 0.028 |
| JBR10.AD+NATCH OS | All (High E2F) | Gender (Male vs Female) | 1.82 | (0.94,3.51) | 0.075 |
| JBR10.AD+NATCH OS | All (High E2F) | Stage (II vs I) | 1.19 | (0.57,2.48) | 0.64 |
| JBR10.AD+NATCH OS | All (High E2F) | Stage (III/IV vs I) | 3.35 | (1.67,6.71) | <0.001 |
| JBR10.AD+NATCH OS | I | E2F score High vs. Low | 4.43 | (1.49,13.12) | 0.007 |
| JBR10.AD+NATCH OS | I | Adjuvant chemotherapy ACT vs. No ACT | 2.02 | (0.66,6.18) | 0.22 |
| JBR10.AD+NATCH OS | I | Gender Male vs. Female | 1.78 | (0.83,3.79) | 0.14 |
| JBR10.AD+NATCH OS | I | E2F/ACT interaction | 0.38 | (0.09,1.61) | 0.19 |
| JBR10.AD+NATCH OS | I (Low E2F) | Adjuvant chemotherapy (ACT vs No ACT) | 2.04 | (0.66,6.26) | 0.21 |
| JBR10.AD+NATCH OS | I (Low E2F) | Gender (Male vs Female) | 1.46 | (0.47,4.49) | 0.51 |
| JBR10.AD+NATCH OS | I (High E2F) | Adjuvant chemotherapy (ACT vs No ACT) | 0.73 | (0.29,1.84) | 0.51 |
| JBR10.AD+NATCH OS | I (High E2F) | Gender (Male vs Female) | 2.12 | (0.75,6.00) | 0.16 |
| JBR10.AD+NATCH OS | II | E2F score High vs. Low | 2.60 | (0.76,8.89) | 0.13 |
| JBR10.AD+NATCH OS | II | Adjuvant chemotherapy ACT vs. No ACT | 1.47 | (0.45,4.83) | 0.53 |
| JBR10.AD+NATCH OS | II | Gender Male vs. Female | 2.63 | (0.93,7.44) | 0.069 |
| JBR10.AD+NATCH OS | II | E2F/ACT interaction | 0.12 | (0.02,0.72) | 0.02 |
| JBR10.AD+NATCH OS | II (Low E2F) | Adjuvant chemotherapy (ACT vs No ACT) | 1.38 | (0.42,4.58) | 0.6 |
| JBR10.AD+NATCH OS | II (Low E2F) | Gender (Male vs Female) | 4.12 | (0.89,19.05) | 0.07 |
| JBR10.AD+NATCH OS | II (High E2F) | Adjuvant chemotherapy (ACT vs No ACT) | 0.22 | (0.06,0.85) | 0.028 |
| JBR10.AD+NATCH OS | II (High E2F) | Gender (Male vs Female) | 1.59 | (0.33,7.60) | 0.56 |
| JBR10.AD+NATCH OS | III/IV | E2F score High vs. Low | 1.63 | (0.41,6.41) | 0.49 |
| JBR10.AD+NATCH OS | III/IV | Adjuvant chemotherapy ACT vs. No ACT | 0.35 | (0.04,3.43) | 0.37 |
| JBR10.AD+NATCH OS | III/IV | Gender Male vs. Female | 1.77 | (0.60,5.21) | 0.3 |
| JBR10.AD+NATCH OS | III/IV | E2F/ACT interaction | 1.69 | (0.14,20.66) | 0.68 |
| JBR10.AD+NATCH OS | III/IV (Low E2F) | Adjuvant chemotherapy (ACT vs No ACT) | 0.43 | (0.04,4.20) | 0.47 |
| JBR10.AD+NATCH OS | III/IV (Low E2F) | Gender (Male vs Female) | NA | ( NA, NA) | NA |
| JBR10.AD+NATCH OS | III/IV (High E2F) | Adjuvant chemotherapy (ACT vs No ACT) | 0.61 | (0.21,1.81) | 0.37 |
| JBR10.AD+NATCH OS | III/IV (High E2F) | Gender (Male vs Female) | 1.72 | (0.58,5.07) | 0.33 |

## Proportional hazard assumption test (Table S20)

Table S20: Proportional hazard assumption test

| effect | cohort | Stage | pvalue_PH | FDR_PH |
| --- | --- | --- | --- | --- |
| Univariable prognostic | MLOS | All (N=300) | 0.7297296 | 1.000 |
| Univariable prognostic | MLOS | Stage I (N=228) | 0.7092857 | 1.000 |
| Univariable prognostic | MLOS | Stage II (N=35) | 0.0304290 | 0.524 |
| Univariable prognostic | MLOS | Stage III/IV (N=32) | 0.8836981 | 1.000 |
| Univariable prognostic | MCLA | All (N=233) | 0.0864463 | 0.524 |
| Univariable prognostic | MCLA | Stage I (N=164) | 0.1143327 | 0.600 |
| Univariable prognostic | MCLA | Stage II (N=42) | 0.7150278 | 1.000 |
| Univariable prognostic | MCLA | Stage III/IV (N=25) | 0.9491921 | 1.000 |
| Univariable prognostic | TCGA | All (N=436) | 0.0805845 | 0.524 |
| Univariable prognostic | TCGA | Stage I (N=235) | 0.5370177 | 1.000 |
| Univariable prognostic | TCGA | Stage II (N=103) | 0.0408029 | 0.524 |
| Univariable prognostic | TCGA | Stage III/IV (N=97) | 0.1945097 | 0.645 |
| Univariable prognostic | LCBRN | All (N=64) | 0.0735160 | 0.524 |
| Univariable prognostic | LCBRN | Stage I (N=49) | 0.2420593 | 0.699 |
| Univariable prognostic | LCBRN | Stage II (N=10) | 0.1101565 | 0.600 |
| Univariable prognostic | LCBRN | Stage III/IV (N=5) | 0.9999944 | 1.000 |
| Univariable prognostic | MLOS+MCLA+TCGA+LCBRN+JBR10.AD | All (N=1065) | 0.0490229 | 0.524 |
| Univariable prognostic | MLOS+MCLA+TCGA+LCBRN+JBR10.AD | Stage I (N=696) | 0.7429014 | 1.000 |
| Univariable prognostic | MLOS+MCLA+TCGA+LCBRN+JBR10.AD | Stage II (N=202) | 0.1407923 | 0.639 |
| Univariable prognostic | MLOS+MCLA+TCGA+LCBRN+JBR10.AD | Stage III/IV (N=159) | 0.4276642 | 0.929 |
| Univariable prognostic | MLCom OS | All (N=101) | 0.0873112 | 0.524 |
| Univariable prognostic | MLCom OS | Stage I (N=72) | 0.3152686 | 0.791 |
| Univariable prognostic | MLCom OS | Stage II (N=13) | 0.2093046 | 0.662 |
| Univariable prognostic | MLCom OS | Stage III/IV (N=16) | 0.9632195 | 1.000 |
| Univariable prognostic | NATCH OS | All (N=40) | 0.3583511 | 0.806 |
| Univariable prognostic | NATCH OS | Stage I (N=22) | 0.1695419 | 0.639 |
| Univariable prognostic | NATCH OS | Stage II (N=6) | 0.9999890 | 1.000 |
| Univariable prognostic | NATCH OS | Stage III/IV (N=12) | 0.6186896 | 1.000 |
| Univariable prognostic | MLCom+NATCH OS | All (N=141) | 0.0622634 | 0.524 |
| Univariable prognostic | MLCom+NATCH OS | Stage I (N=94) | 0.2492937 | 0.699 |
| Univariable prognostic | MLCom+NATCH OS | Stage II (N=19) | 0.0562446 | 0.524 |
| Univariable prognostic | MLCom+NATCH OS | Stage III/IV (N=28) | 0.6002706 | 1.000 |
| Univariable prognostic | MLCom PFS | All (N=98) | 0.0033824 | 0.213 |
| Univariable prognostic | MLCom PFS | Stage I (N=71) | 0.0465955 | 0.524 |
| Univariable prognostic | MLCom PFS | Stage II (N=12) | 0.1084701 | 0.600 |
| Univariable prognostic | MLCom PFS | Stage III/IV (N=15) | 0.7322514 | 1.000 |
| Univariable prognostic | NATCH PFS | All (N=40) | 0.1750262 | 0.639 |
| Univariable prognostic | NATCH PFS | Stage I (N=22) | 0.1775522 | 0.639 |
| Univariable prognostic | NATCH PFS | Stage II (N=6) | 0.9999890 | 1.000 |
| Univariable prognostic | NATCH PFS | Stage III/IV (N=12) | 0.1609423 | 0.639 |
| Univariable prognostic | MLCom+NATCH PFS | All (N=138) | 0.0008650 | 0.109 |
| Univariable prognostic | MLCom+NATCH PFS | Stage I (N=93) | 0.0127571 | 0.524 |
| Univariable prognostic | MLCom+NATCH PFS | Stage II (N=18) | 0.0300932 | 0.524 |
| Univariable prognostic | MLCom+NATCH PFS | Stage III/IV (N=27) | 0.4855583 | 0.971 |
| Univariable prognostic | JBR10 OS | All (N=62) | 0.4660138 | 0.971 |
| Univariable prognostic | JBR10 OS | Stage I (N=34) | 0.4071942 | 0.900 |
| Univariable prognostic | JBR10 OS | Stage II (N=28) | 0.0788155 | 0.524 |
| Univariable prognostic | JBR10 AD | All (N=32) | 0.6571584 | 1.000 |
| Univariable prognostic | JBR10 AD | Stage I (N=20) | 0.3446465 | 0.804 |
| Univariable prognostic | JBR10 AD | Stage II (N=12) | 0.2607507 | 0.699 |
| Univariable prognostic | NATCH OS | All (N=40) | 0.3583511 | 0.806 |
| Univariable prognostic | NATCH OS | Stage I (N=22) | 0.1695419 | 0.639 |
| Univariable prognostic | NATCH OS | Stage II (N=6) | 0.9999890 | 1.000 |
| Univariable prognostic | NATCH OS | Stage III/IV (N=12) | 0.6186896 | 1.000 |
| Univariable prognostic | JBR10+NATCH OS | All (N=102) | 0.1937710 | 0.645 |
| Univariable prognostic | JBR10+NATCH OS | Stage I (N=56) | 0.7000626 | 1.000 |
| Univariable prognostic | JBR10+NATCH OS | Stage II (N=34) | 0.0862656 | 0.524 |
| Univariable prognostic | JBR10+NATCH OS | Stage III/IV (N=12) | 0.6186896 | 1.000 |
| Univariable prognostic | JBR10 AD+NATCH OS | All (N=72) | 0.2416035 | 0.699 |
| Univariable prognostic | JBR10 AD+NATCH OS | Stage I (N=42) | 0.6991082 | 1.000 |
| Univariable prognostic | JBR10 AD+NATCH OS | Stage II (N=18) | 0.1872941 | 0.645 |
| Univariable prognostic | JBR10 AD+NATCH OS | Stage III/IV (N=12) | 0.6186896 | 1.000 |
| Univariable prognostic | JBR10 DSS | All (N=62) | 0.1762174 | 0.639 |
| Univariable prognostic | JBR10 DSS | Stage I (N=34) | 0.9223927 | 1.000 |
| Univariable prognostic | JBR10 DSS | Stage II (N=28) | 0.0793689 | 0.524 |
| Univariable prognostic | JBR10 AD DSS | All (N=32) | 0.6571584 | 1.000 |
| Univariable prognostic | JBR10 AD DSS | Stage I (N=20) | 0.3446465 | 0.804 |
| Univariable prognostic | JBR10 AD DSS | Stage II (N=12) | 0.2607507 | 0.699 |
| Univariable prognostic | NATCH PFS | All (N=40) | 0.1750262 | 0.639 |
| Univariable prognostic | NATCH PFS | Stage I (N=22) | 0.1775522 | 0.639 |
| Univariable prognostic | NATCH PFS | Stage II (N=6) | 0.9999890 | 1.000 |
| Univariable prognostic | NATCH PFS | Stage III/IV (N=12) | 0.1609423 | 0.639 |
| Univariable predictive | JBR10 OS | All Low (N=58) | 0.9900000 | 1.000 |
| Univariable predictive | JBR10 OS | NA High (N=75) | 0.8000000 | 1.000 |
| Univariable predictive | JBR10 OS | Stage I Low (N=37) | 0.9100000 | 1.000 |
| Univariable predictive | JBR10 OS | NA High (N=36) | 0.4800000 | 0.971 |
| Univariable predictive | JBR10 OS | Stage II Low (N=21) | 0.9200000 | 1.000 |
| Univariable predictive | JBR10 OS | NA High (N=39) | 1.0000000 | 1.000 |
| Univariable predictive | JBR10 AD | All Low (N=48) | 0.9900000 | 1.000 |
| Univariable predictive | JBR10 AD | NA High (N=23) | 0.8600000 | 1.000 |
| Univariable predictive | JBR10 AD | Stage I Low (N=30) | 0.8800000 | 1.000 |
| Univariable predictive | JBR10 AD | NA High (N=12) | 0.3000000 | 0.771 |
| Univariable predictive | JBR10 AD | Stage II Low (N=18) | 0.8000000 | 1.000 |
| Univariable predictive | JBR10 AD | NA High (N=11) | 0.7000000 | 1.000 |
| Univariable predictive | NATCH OS | All Low (N=23) | 0.3300000 | 0.800 |
| Univariable predictive | NATCH OS | NA High (N=51) | 0.8400000 | 1.000 |
| Univariable predictive | NATCH OS | Stage I Low (N=16) | 0.3200000 | 0.791 |
| Univariable predictive | NATCH OS | NA High (N=22) | 0.6800000 | 1.000 |
| Univariable predictive | NATCH OS | Stage II Low (N=2) | NA | NA |
| Univariable predictive | NATCH OS | NA High (N=11) | 0.2300000 | 0.699 |
| Univariable predictive | NATCH OS | Stage III/IV Low (N=5) | 0.5600000 | 1.000 |
| Univariable predictive | NATCH OS | NA High (N=18) | 0.0520000 | 0.524 |
| Univariable predictive | JBR10+NATCH OS | All Low (N=81) | 0.7400000 | 1.000 |
| Univariable predictive | JBR10+NATCH OS | NA High (N=126) | 0.6300000 | 1.000 |
| Univariable predictive | JBR10+NATCH OS | Stage I Low (N=53) | 0.9700000 | 1.000 |
| Univariable predictive | JBR10+NATCH OS | NA High (N=58) | 0.9200000 | 1.000 |
| Univariable predictive | JBR10+NATCH OS | Stage II Low (N=23) | 0.8200000 | 1.000 |
| Univariable predictive | JBR10+NATCH OS | NA High (N=50) | 0.8500000 | 1.000 |
| Univariable predictive | JBR10+NATCH OS | Stage III/IV Low (N=5) | 0.5600000 | 1.000 |
| Univariable predictive | JBR10+NATCH OS | NA High (N=18) | 0.0520000 | 0.524 |
| Univariable predictive | JBR10 AD+NATCH OS | All Low (N=71) | 0.7900000 | 1.000 |
| Univariable predictive | JBR10 AD+NATCH OS | NA High (N=74) | 0.8600000 | 1.000 |
| Univariable predictive | JBR10 AD+NATCH OS | Stage I Low (N=46) | 0.8800000 | 1.000 |
| Univariable predictive | JBR10 AD+NATCH OS | NA High (N=34) | 0.6600000 | 1.000 |
| Univariable predictive | JBR10 AD+NATCH OS | Stage II Low (N=20) | 0.9600000 | 1.000 |
| Univariable predictive | JBR10 AD+NATCH OS | NA High (N=22) | 0.4500000 | 0.961 |
| Univariable predictive | JBR10 AD+NATCH OS | Stage III/IV Low (N=5) | 0.5600000 | 1.000 |
| Univariable predictive | JBR10 AD+NATCH OS | NA High (N=18) | 0.0520000 | 0.524 |
| Univariable predictive | JBR10 DSS | All Low (N=58) | 0.7900000 | 1.000 |
| Univariable predictive | JBR10 DSS | NA High (N=75) | 0.6100000 | 1.000 |
| Univariable predictive | JBR10 DSS | Stage I Low (N=37) | 0.9700000 | 1.000 |
| Univariable predictive | JBR10 DSS | NA High (N=36) | 0.2500000 | 0.699 |
| Univariable predictive | JBR10 DSS | Stage II Low (N=21) | 0.8500000 | 1.000 |
| Univariable predictive | JBR10 DSS | NA High (N=39) | 0.8600000 | 1.000 |
| Univariable predictive | JBR10 AD DSS | All Low (N=48) | 0.7900000 | 1.000 |
| Univariable predictive | JBR10 AD DSS | NA High (N=23) | 0.5600000 | 1.000 |
| Univariable predictive | JBR10 AD DSS | Stage I Low (N=30) | 0.9400000 | 1.000 |
| Univariable predictive | JBR10 AD DSS | NA High (N=12) | 0.1200000 | 0.605 |
| Univariable predictive | JBR10 AD DSS | Stage II Low (N=18) | 0.9600000 | 1.000 |
| Univariable predictive | JBR10 AD DSS | NA High (N=11) | 0.7000000 | 1.000 |
| Univariable predictive | NATCH PFS | All Low (N=23) | 0.0590000 | 0.524 |
| Univariable predictive | NATCH PFS | NA High (N=51) | 0.4800000 | 0.971 |
| Univariable predictive | NATCH PFS | Stage I Low (N=16) | 0.5700000 | 1.000 |
| Univariable predictive | NATCH PFS | NA High (N=22) | 0.2100000 | 0.662 |
| Univariable predictive | NATCH PFS | Stage II Low (N=2) | NA | NA |
| Univariable predictive | NATCH PFS | NA High (N=11) | 0.8400000 | 1.000 |
| Univariable predictive | NATCH PFS | Stage III/IV Low (N=5) | 1.0000000 | 1.000 |
| Univariable predictive | NATCH PFS | NA High (N=18) | 0.2800000 | 0.735 |

## ROC curves showing added value of E2F gene signature (Table S21)

Table S21: Added value of E2F signature

|  | Cohort | Outcome | Predictors | AUC (median) | 95% CI of AUC |
| --- | --- | --- | --- | --- | --- |
| 3 | MLOS+TCGA+MCLA+LCBRN+JBR10.AD | OS | stage | 0.620 | 0.597-0.643 |
| 4 | MLOS+TCGA+MCLA+LCBRN+JBR10.AD | OS | stage, PC1 | 0.653 | 0.628-0.677 |
| 7 | MLCom+NATCH | OS | stage | 0.602 | 0.56-0.648 |
| 8 | MLCom+NATCH | OS | stage, PC1 | 0.656 | 0.609-0.704 |
| 11 | MLCom+NATCH | PFS | stage | 0.591 | 0.551-0.632 |
| 12 | MLCom+NATCH | PFS | stage, PC1 | 0.633 | 0.589-0.68 |

## Median survival time of published JBR10 and NATCH trials

### 1). JBR10 trial

The 5-year survival rates were 69% and 54% in the adjuvant chemotherapy arm and control arm. In the subset of cohort used in our study, the 5-year survival rates were 71.7% and 53.9% in the adjuvant chemotherapy arm and control arm.

The median survival time in the JBR10 trial was 94 months in the adjuvant chemotherapy arm and 73 months in the non-adjuvant chemotherapy arm (HR=0.65, 95% CI: 0.52-0.91) for all stages. In subset of cohort used in our study, the median survival time was not reached in the adjuvant chemotherapy group and 74 months in the non-adjuvant chemotherapy group (HR=0.8, 95% CI: 0.48-1.32). For subgroup analysis, the median survival time was 80 months in the ACT group and 41 months in the non-ACT group in stage II patients (HR=0.59, 95% CI: 0.42-0.85). In our cohort, the median survival time was 94 months in the ACT group and 32 months in the non-ACT group in stage II patients (HR=0.59, 95% CI: 0.3-1.18).

### 2). NATCH trial

The 5-year survival rate in the NATCH trial was 44% in the surgery arm and 45.5% in the adjuvant chemotherapy arm for all stages. The hazard ratio was 1.01 (95% CI: 0.62-1.65, ACT vs. no ACT). For stage II patients, the 5-year survival rate was 34.5% and 36.6%, in the surgery arm and the adjuvant chemotherapy arm respectively. However, the complete cohort consists of mixed histology types, including adenocarcinoma, squamous cell carcinoma, and others. Only adenocarcinoma samples were used in our study. The 5-year survival rate in our study was 37.3% in the surgery group and 50.3% in the adjuvant chemotherapy group for all stages and the hazard ratio was 0.75 (95% CI: 0.4-1.39) . For stage II patients, the 5-year survival rate was 33.3% and 55.6%, in the surgery arm and the adjuvant chemotherapy arm, respectively.

## References:

1. Sanchez-Palencia A, Gomez-Morales M, Gomez-Capilla JA, Pedraza V, Boyero L, Rosell R, Fárez-Vidal ME. Gene expression profiling reveals novel biomarkers in nonsmall cell lung cancer. Int J Cancer. 2011; 129: 355-64.

2. Hou J, Aerts J, den Hamer B, van Ijcken W, den Bakker M, Riegman P, van der Leest C, van der Spek P, Foekens JA, Hoogsteden HC, Grosveld F, Philipsen S. Gene expression-based classification of non-small cell lung carcinomas and survival prediction. PLoS ONE. 2010; 5: e10312.

3. Felip E, Rosell R, Maestre JA, Rodríguez-Paniagua JM, Morán T, Astudillo J, Alonso G, Borro JM, González-Larriba JL, Torres A, Camps C, Guijarro R, Isla D, et al. Preoperative chemotherapy plus surgery versus surgery plus adjuvant chemotherapy versus surgery alone in early-stage non-small-cell lung cancer. J Clin Oncol. 2010; 28: 3138-45.

4. Schabath MB, Welsh EA, Fulp WJ, Chen L, Teer JK, Thompson ZJ, Engel BE, Xie M, Berglund AE, Creelan BC, Antonia SJ, Gray JE, Eschrich SA, et al. Differential association of STK11 and TP53 with KRAS mutation-associated gene expression, proliferation and immune surveillance in lung adenocarcinoma. Oncogene. 2015; doi: 10.1038/onc.2015.375.

5. Director's Challenge Consortium for the Molecular Classification of Lung Adenocarcinoma, Shedden K, Taylor JM, Enkemann SA, Tsao MS, Yeatman TJ, Gerald WL, Eschrich S, Jurisica I, Giordano TJ, Misek DE, Chang AC, Zhu CQ, et al. Gene expression-based survival prediction in lung adenocarcinoma: a multi-site, blinded validation study. Nat Med. 2008; 14: 822-7.

6. Cancer Genome Atlas Research Network. Comprehensive genomic characterization of squamous cell lung cancers. Nature. 2012; 489: 519-25.

7. TCGA. Comprehensive molecular profiling of lung adenocarcinoma. Nature. 2014; 511: 543-50.

8. Zhu CQ1, Ding K, Strumpf D, Weir BA, Meyerson M, Pennell N, Thomas RK, Naoki K, Ladd-Acosta C, Liu N, Pintilie M, Der S, Seymour L, Jurisica I, et al. Prognostic and predictive gene signature for adjuvant chemotherapy in resected non-small-cell lung cancer. J Clin Oncol. 2010; 28: 4417-24.

9. Welsh EA, Eschrich SA, Berglund AE, Fenstermacher DA. Iterative rank-order normalization of gene expression microarray data. BMC Bioinformatics. 2013; 14: 153.

10. Johnson WE, Li C, Rabinovic A. Adjusting batch effects in microarray expression data using empirical Bayes methods. Biostatistics. 2007; 8: 118-27.

11. Waggott D, Chu K, Yin S, Wouters BG, Liu FF, Boutros PC. NanoStringNorm: An Extensible R Package For the Pre-Processing of NanoString mRNA and miRNA Data. Bioinformatics. 2012.

12. Ma Y, Croxton R, Moorer RL, Jr., Cress WD. Identification of novel E2F1-regulated genes by microarray. Arch Biochem Biophys. 2002; 399: 212-24.
